# Generation of divergent uroplakin tetraspanins and their partners during vertebrate evolution: identification of novel uroplakins

**DOI:** 10.1186/1471-2148-14-13

**Published:** 2014-01-23

**Authors:** Rob DeSalle, Javier U Chicote, Tung-Tien Sun, Antonio Garcia-España

**Affiliations:** 1 Sackler Institute for Comparative Genomics, American Museum of Natural History, New York, New York, USA; 2 Unitat de Recerca, Hospital Joan XXIII, Institut de Investigacio Sanitaria Pere Virgili (IISPV), Universitat Rovira i Virgili, Tarragona, Spain; 3 Department of Cell Biology, New York University School of Medicine, New York, New York, USA; 4 Department of Dermatology, New York University School of Medicine, New York, New York, USA; 5 Department of Biochemistry and Molecular Pharmacology, New York University School of Medicine, New York, New York, USA; 6 Department of Urology, New York University School of Medicine, New York, New York, USA

## Abstract

**Background:**

The recent availability of sequenced genomes from a broad array of chordates (cephalochordates, urochordates and vertebrates) has allowed us to systematically analyze the evolution of uroplakins: tetraspanins (UPK1a and UPK1b families) and their respective partner proteins (UPK2 and UPK3 families).

**Results:**

We report here: (1) the origin of uroplakins in the common ancestor of vertebrates, (2) the appearance of several residues that have statistically significantly positive dN/dS ratios in the duplicated paralogs of uroplakin genes, and (3) the existence of strong coevolutionary relationships between UPK1a/1b tetraspanins and their respective UPK2/UPK3-related partner proteins. Moreover, we report the existence of three new UPK2/3 family members we named UPK2b, 3c and 3d, which will help clarify the evolutionary relationships between fish, amphibian and mammalian uroplakins that may perform divergent functions specific to these different and physiologically distinct groups of vertebrates.

**Conclusions:**

Since our analyses cover species of all major chordate groups this work provides an extremely clear overall picture of how the uroplakin families and their partner proteins have evolved in parallel. We also highlight several novel features of uroplakin evolution including the appearance of UPK2b and 3d in fish and UPK3c in the common ancestor of reptiles and mammals. Additional studies of these novel uroplakins should lead to new insights into uroplakin structure and function.

## Background

Uroplakins (UP’s) are the protein subunits of the urothelial plaques that cover the apical surface of mammalian bladder epithelium (urothelium). There are four major mammalian uroplakins, i.e., the 27-kDa UPIa, 28-kDa UPIb, 15-kDa UPII and the 47-kDa UPIIIa; [1-3]. UPK3b is a minor isoform of UPIIIa [4]. These plaques form the so-called asymmetric unit membrane (AUM), and contribute to the permeability barrier function and mechanical stability of the urothelium. Uroplakin defects underlie some urinary tract anomalies, and one of the uroplakins, UPIa, can serve as the receptor for the uropathogenic E. coli that causes over 85% of urinary tract infections [5].

Uroplakins (UPK) can be divided into two types. The first type comprises UPK1a and 1b, which belong to the tetraspanin family (containing CD9, CD63, CD81 and CD151 proteins); tetraspanin proteins span the membrane four times and play important functions in fertilization, immunity and cell:cell interaction [6-11]. The second type comprises UPK2 and UPK3 that span the membrane only once; these uroplakins share a stretch of ~12 amino acid residues on the extracellular side of their single transmembrane domain (TMD) [12,13].

The fact that uroplakins 1a and 1b (UPK1a and UPK1b) interact specifically with uroplakins 2 and 3a (UPK2 and UPK3a), respectively, make them an attractive system for studying the co-evolution of interacting membrane protein pairs [14-16]. While mammalian uroplakins form 2D crystals of urothelial plaques on urothelial apical surface, uroplakins of the non-mammals including amphibians (which have the complete assortment of UPK1a, 1b, 2 and 3a, as well the minor UPK3b) do not form such plaques [17-19]. In *Xenopus* oocytes, UPK3a and its binding partner UPK1b play a key role in sperm-egg fertilization [19-21]. In addition, a UPK3-related gene product in zebrafish [22] was recently found to play a role in epithelial polarization and morphogenesis of pronephric tubules [20]. The evolutionary relationship among these fish, amphibian and mammalian uroplakins, that seem to be functionally divergent, remains unclear.

To better understand the evolution of uroplakins and to decipher how the tetraspanin uroplakins coevolve with their binding partners, we analyzed the uroplakin-related sequences in a wide range of whole-genome-sequenced vertebrate species including mammals, birds, amphibians, bony fish and ancient cartilaginous fish [22]. Previously we showed the existence of a strong co- evolutionary relationship between UPK1a and UPK1b and their partner’s UPK2 and UPK3a/3b proteins, respectively [22]. The recent availability of additional genome-sequences from a broad array of chordates (cephalochordates, urochordates and vertebrates), including “living fossils” such as lampreys, spotted gars and coelacanths, allowed us to re-examine more systematically the evolution and possible neofunctionalization of uroplakins. For convenience and consistency, in this communication we will refer to the individual ortholog groups such as UPK1a, UPK1b, UPK2 and UPK3a as families, while the UPK1a/1b tetraspanins and the UPK2/UPK3-related proteins as two separate *super*families.

In this paper, we pinpoint the origin of uroplakins in the common ancestor of vertebrates, track the appearance of skewed dn/dS ratios in the nucleotide sequences of the gene families and point to possible neofunctionalization in the duplication of paralog uroplakin genes. We also analyze the patterns of coevolution between UPK1a/1b tetraspanins and the UPK2/UPK3-related proteins. Finally, we report the existence of three new UPK members belonging to the UPK2/3 superfamily, i.e., UPK2b, 3c and 3d. Since our analyses are based on a broad array of species covering all major chordate groups this work presents an overall picture of the uroplakin families existing in nature.

## Methods

### Sequences and matrix construction

All protein and DNA sequences used in this study (tetraspanin UPK’s, i.e., UPK1a and UPK1b and single membrane spanning UPK’s, i.e., UPK2 and UPK3) are listed in Additional file 1: Figure S1 and Additional file 2: Figure S2, in which exons 2–5 are represented in alternate colours in the protein sequences. Blast searches with the Blast-T program were performed as described [22-24] with multiple starting queries using various genome-sequencing projects including the NCBI (http://www.ncbi.nlm.nih.gov; http://www.ncbi.nlm.nih.gov/sutils/genom_table.cgi?organism=euk), Ensembl (http://www.ensembl.org), http://www.ambystoma.org/ servers and ESTs databases. Intron-exon borders were determined as in [17] using the “align two sequences” option of the NCBI BLAST program (http://www.ncbi.nlm.nih.gov). Splice consensus signals were then manually annotated.

### Cloning and sequencing of UPK 3c

Total human normal bladder mRNA (1100564 F, Asterand, Detroit, MI) was used to synthesize cDNA using Transcriptor Fisrt Strand cDNA Synthesis Kit (Roche, Germany) with Random Hexamer Primers. The human normal bladder cDNA of upk3c was isolated by RT-PCR using primers based on the hypothetical uroplakin 3BL sequence annotated in NCBI (NM_001114403.2). The primer sequences used for full-length ORF amplification were sense 5′- GACGGACGGACAGACAGATGGACA-3′ and antisense 5′-GCCCCTCTGGAACCCCTCAG-3′. The cDNA product was cloned into pCR®II- TOPO vector and sequenced.

### Alignments

Fasta sequences were aligned using the web based alignment tool TranslatorX [25] that utilizes amino acid alignments to generate DNA sequence alignments. Phylogenetic matrices in PHYLIP and NEXUS format were then generated using Mesquite [26] for both protein sequences and DNA sequences. We explored the different phylogenetic signal inherent in amino acid data and nucleotide data, by analysing the protein and DNA sequence matrices separately. In addition, we elided the DNA data matrix with the amino acid matrix for an analysis where the amino acid data weight the DNA sequence data [27]. PHYLIP matrices were then used in subsequent analysis for natural selection (web based DataMonkey analyses and desktop HYPHY analyses). In addition to the two differently formatted matrices (PHYLIP versus NEXUS), we also generated two kinds of matrices. The first kind of matrix used the genes in the two gene families as terminals. One matrix for the UPK1 genes (UPK1a and UPK1b) was constructed and a second matrix for the UPK2/UPK3 families was also constructed. The second kind of matrix we constructed used the several vertebrate species that have UPKs in their genomes as terminals with partitions representing the seven paralog groups for these genes.

### Phylogenetic analysis

**Figure 1:**
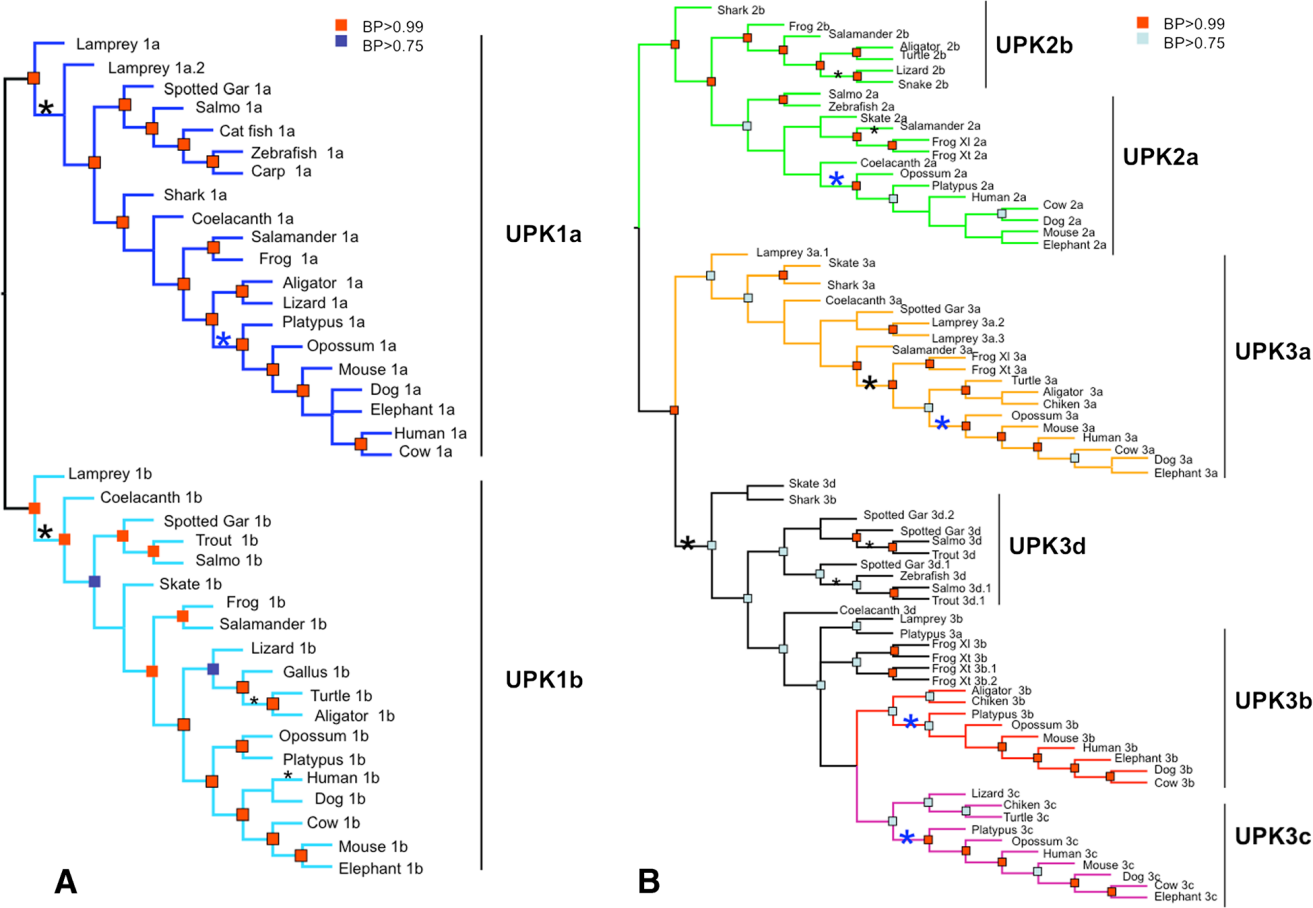
**Clustering of new UPK members.** Clustering of UPK2b, UPK3c and UPK3d in mixed DNA/protein trees showing the evolutionary relationships between UPKs. **(A)** Tetraspanin uroplakins, and **(B)** UPK2/3 uroplakins. The major groups of uroplakins are indicated by vertical lines in the figure. Separate protein and DNA parsimony, maximum likelihood and Bayesian phylogenetic trees are included in Additional file 3: Figure S3 and Additional file 4: Figure S4. Red rectangles BP (Bayesian posterior) >0.99; Blue rectangles BP > 0.75. Asterisks represent statistically significant (p < 0.05) branches where dN/dS ratios are significantly greater than 1.0. The large asterisks mark deep branches and the small asterisks mark more terminal branches. The blue asterisks refer to branches where dN/dS > 1.0 in the common ancestor of mammals.

Three kinds of tree building approaches were used to generate phylogenetic hypotheses for the gene families in this study. Parsimony analysis was accomplished in PAUP. Maximum Likelihood analysis was accomplished using the RaxML BlackBox webserver [28]. Bootstraps in both PAUP and RaxML were generated using 100 replicates of bootstrapping. Bayesian analysis was accomplished in MrBayes [29,30]. For each of the gene family trees, two million generations of MCMC simulation were used along with a burnin of 200,000 generations to generate the Baysian posteriors for the two gene family trees. This number of MCMC generations allowed for convergence of simulation chains and reduction of split frequencies to an acceptable level. Here we report the Bayesian phylogenetic inference results (Figure 1). Phylogenetic trees are stored in Additional file 3: Figure S3 and Additional file 4: Figure S4.

### Detection of dN/dS skew

Two tests were used to detect the patterns of sequence change using dN/dS ratios in the gene families of this study. The first test examines branch specific departure from neutrality (or a dN/dS = 1.0). The Branch-site REL test in the HYPHY package was used on the two gene families (*UPK1* and *UPK2*/*UPK3*) separately. The default settings and the Bayesian tree topology were used with in these tests. The second test is the MEME (Mixed Effects Model of Evolution) test that uses mixed model approaches to detect departures from neutrality at individual codons [31]. This latter test was performed individually on each of the following seven genes – *UPK1a, UPK1b, UPK2, UPK2a, UPK3a, UPK3b,* and *UPK3c*.

### Analysis of gene by gene phylogenetic interaction

The congruence of the UPK interacting pairs was examined using the Shimodaira Hasegawa test [32]. This test examines the congruence of phylogenetic information in two partitions of data using a likelihood ratio test. Each of the seven genes (*UPK1a, UPK1b, UPK2a, UPK2b, UPK3a, UPK3b, UPK3c*) that are found in more than four species were tested pairwise for congruence with each other.

## Results and discussion

### Vertebrate origin and evolution of uroplakins

In our earlier work [22], we suggested that uroplakins first appeared in the common ancestor of vertebrates because the oldest uroplakin sequences we detected were of cartilagenous fish [22]. With the availability of greatly expanded genomic databases of chordates (Vertebrates, Cephalochordates and Urochordates), we have found UPK-related sequences in lampreys (extant jawless basal vertebrates called agnathans) but not in Cephalochordates (*Amphioxus*), Urochordates (*Ciona*) or lower organisms. This finding suggests that UPKs originated in the common ancestor of vertebrates over 500 mya when vertebrates radiated from cephalochordates and urochordates and most likely underwent two rounds of whole genome duplication (WGD) [33-36].

We used the elided matrix described in the materials and methods to generate the Bayesian trees for UPK1a/1b superfamily (Figure 1A) and UPK2/3 superfamily (Figure 1B), which represent different paralogs. Separate protein and DNA phylogenetic trees for these two gene superfamilies based on parsimony, maximum likelihood and Bayesian approaches are included in Additional file 3: Figure S3 and Additional file 4: Figure S4. Previous phylogenetic analyses showed that tetraspanin UPK1a’s and 1b’s form a tight clade within the broad superfamily of eukaryotic tetraspanins [24,37-39]. The analysis of tetraspanin UPK1a and 1b (Figure 1A) shows that their genealogy agrees with animal phylogeny except for UPK1a and UPK1b from cartilaginous fish that are closer to tetrapods than to bony fish. This deviation probably reflects the well known high diversification and faster evolving rates of bony fish in comparison with tetrapod and cartilaginous fish [40].

**Figure 2:**
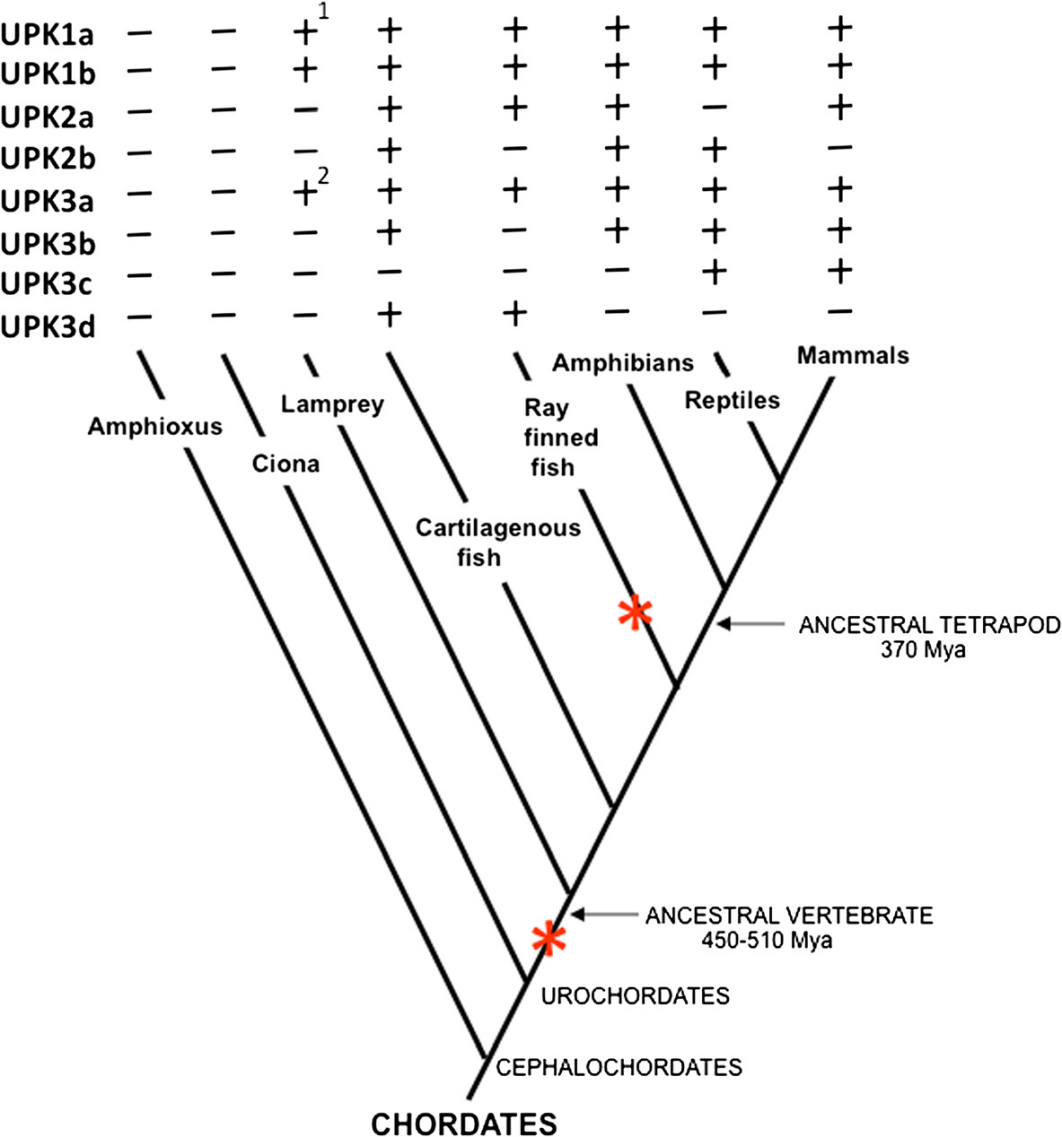
**Presence (+) and absence (−) of UPKs represented in a simplified phylogenetic tree of chordates.** Extinct lineages, hagfish, lungfish and coelacanth lineages have been omitted for simplicity. Reptiles represent Reptiles plus Aves (Sauropsidae). Red asterisks are position in the phylogeny where whole genome duplication events occurred Mya,=1.0 million years. Footnote 1 indicates that there are two forms of this family member, and footnote 2 indicates that there are three members of this family member.

The analyses of the evolutionary relationships among the members of the UPK2 and UPK3 superfamilies using several different phylogenetic approaches yielded consistent results, which revealed the existence of three new UPK paralogs (Figures 1B and 2; see also Additional file 1: Figure S1 and Additional file 2: Figure S2). First, we found a new paralog group that we named UPK3c, which was formed through a duplication of UPK3b in the common ancestors of Sauropsidae (reptiles and birds) and Mammals (data not shown). Of the three newly found uroplakin paralogs, UPK3c is the only one present in reptiles, birds and mammals including humans (Figure 2). We confirmed the existence of this new class of UPIIIc in humans by isolating and sequencing its full length cDNA by RT-PCR using total bladder RNA as the Template. A comparison of UPK3c and UPK3b protein sequences is shown in Additional file 5: Figure S5. The identity between human UPK3b and 3c protein sequences is 37%, which is similar to the identity between 3b and 3a (34%; [4]). Although chicken UPK3c still possesses a motif that weakly resembles the conserved ~12 amino acid stretch characteristic of UPK3a and 3b [4], this motif is missing in mammalian UPK3c’s (Additional file 5: Figure S5). Furthermore the cytosolic tail of UPK3c is 17 amino acids shorter than human UPK3b. Second, we found another new and relatively primitive paralog within the UPK3 family, that we named UPK3d. UPK3d’s are closely related to UPK3b’s but exist only in fishes (Figure 2). This uroplakin may correspond to the so-called UPK3-like protein in zebrafish recently reported to play a role in the development of zebrafish pronephric tubule cell function, polarization and morphogenesis [41]. Finally, we identified a new and also relatively primitive paralog group of UPK2-related genes that exist only in shark, frog, salamander and reptiles, but not in mammals. We named this group UPK2b, to distinguish it from the original UPK2 that we now call UPK2a and is present in all vertebrates.

The genealogy of UPK2/3, like that of UPK1a/1b (Figure 1B), is consistent with the organismal histories, with a few exceptions. For example, the lamprey UPK2a, as well as platypus UPK3a, coelacanth 3a, and lamprey 3a.1 and 3a.2 have highly divergent sequences that did not cluster with their respective groups (Figure 1B). These incongruence might be caused by long branch attraction; an analysis artifact in which rapidly evolving sequences cluster together regardless of their correct relationships [42]. Alternatively, these proteins may be converging in function.

### Gene duplication and hypotheses of neofunctionalation of uroplakins

To examine further the patterns of sequence change in the uroplakin genes, we established where in the phylogeny of vertebrates branch specific changes in intensity and direction of skew in dN/dS ratios occurred. We also determined which residues in the uroplakin proteins where statistically significant departure from dN/dS = 1.0 occur. There are three outcomes of using dN/dS as an indicator of sequence change. The first is when the ratio is significantly less than 1.0 (often times in the literature equated to purifying selection). Another case is where the gene sequences will accumulate silent (synonymous) and replacement (non-synonymous) substitutions in its DNA sequence in equal proportion, and hence have a dN/dS = 1.0 (often times equated in the literature with neutrality). The final and more rare possibility is that the site will or branch will have a statistically significant dN/dS ratio greater than 1.0 (often times in the literature referred to as positive Darwinian selection). Since the validity of equating the skew in these ratios has recently been called into question [43,44] we prefer here to simply point out a pattern of departure form the neutral expectation (dN/dS = 1.0) when we observe a statistically significant result. Whether or not natural selection is at work in molding the skewed ratios is dependent on functional experiments and validation. We suggest however that significantly skewed branch or residues show the potential for evolutionarily important events and reporting the location of these skewed residues and branches will be useful to subsequent researchers working on the function and evolution of these proteins.

We thus identified the branches that have experienced statistically significant departure from neutrality in their dN/dS ratios in the uroplakin genealogies (Figure 1A and B). These analyses led to two major findings. First, in almost every uroplakin paralog group (UPK1a, 1b, 2a, 3a, 3b and 3c) a strong pattern of significant skew toward dN/dS > 1.0 accompanies the duplication that produced the paralog group (asterisks in Figure 1). Second, the divergence of mammal species is also accompanied by significant skew in sequence change (blue asterisks in Figure 1). The single exception to this pattern is for the mammalian UPK1b group.

**Figure 3:**
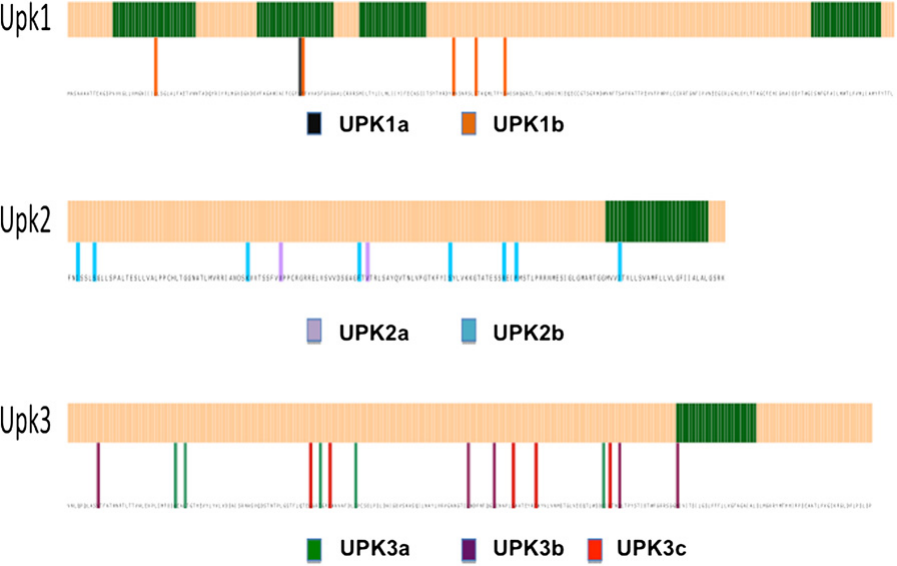
**Positions in the UPK genes where departure from neutrality occurs.** MEME analysis was performed on individual codons in each of the following seven genes UPK1a, UPK1b, UPK2, UPK2a, UPK3a, UPK3b, and UPK3c to generate this figure.

To examine the evolution of individual UPK paralog groups in more detail, we calculated the Omega values (Dn/Ds) of each amino acid residue. This analysis identified many amino acid residues that are significantly greater than 1.0 for dN/dS ratio in uroplakin genes (Figure 3). Interestingly, most of these highly selected residues, which could be involved in the evolution of novel function for these proteins which are located in non-transmembrane regions. This finding is consistent with earlier reports that the TM domains of tetraspanins interact closely with one another and with those of the their partner proteins [45,46] and with our own finding that the integrity of the TM domains of UPK1b was crucially important for the protein to be able to exit from the ER [16].

Another interesting finding is that some uroplakin paralogs have higher levels dN/dS skew than others. For instance, while UPK1a has only a single codon with dN/dS > 1.0, UPK1b has five. UPK2a has two codons with dN/dS > 1.0, while UPK2b has eight. The UPK3 paralogs (UPK3a, UPK3b and UPK3c) however show similar levels of dN/dS > 1.0 (five codons in each).

These results are relevant to establishing hypotheses about the function and possible neofuntionalization of the uroplakin gene families. It is possible that after a gene family is duplicated the branch with more residues that are changing disproprortinately is the paralog that has gained novel function. Purifying natural selection often relaxes after the duplication of a gene family allowing for the neofunctionalization of the newly duplicated paralog [33-36]. In order for neofunctionalization to occur the variation in nonsynonymous sites would need to be present and residues with dN/dS > 1.0 that are statistically significant would be good candidates for such neofunctionalization. Our results would then indicate that of the UPK1 paralogs (UPK1a and UPK1b), it is UPK1b that has the potential to be neofunctionalized. In this hypothesis, UPK1a would then have retained the ancestral protein function, while UPK1b would have evolved a new but related function to UPK1a.

Likewise, there are two points in the evolution of the gene family where we can hypothesize neofunctionalization events in the UPK2/UPK3 subfamilies. UPK2a has the lowest number of codons with positively skewed codons of the UPK2/UPK3 uroplakins, making it the more conserved in sequence and hence more than likely the more conserved in function. UPK2b and all of the UPK3’s (UPK3a, UPK3b and UPK3c) on the other hand have the potential to have been neofunctionalized producing newer and more derived functions and hence have more codons with dN/dS > 1.0.

These patterns of sequence divergence patterns for the tetraspanin UPKs and the UPK2/UPK3 proteins fit nicely with what we know about their coevolution and cofunctionality (see below). Since UPK1a physically interacts with UPK2a, then the genes for these two proteins should have similar patterns of sequence change (as manifest in dN/dS ratios). Likewise if UPK1b and UPK3 are physically interacting then we should also see similar patterns of sequence change for the genes for those proteins. Indeed, UPK1a and UPK2a show the largest number of positively skewed dN/dS ratios and hence show a hypothesized ancestral function, while UPK1b, UPK2b and UPK3a, b and c show potential patterns of neofunctionalization.

### Uroplakin evolution and diversification of major vertebrete groups

While the formation of tetraspanin UPK’s, i.e., UPK1a and UPK1b, can be easily explained by a single duplication event in the common ancestor of vertebrates, the evolution of the UPK2/UPK3 families is more complex requiring several rounds of duplication events to explain the distribution of genes in the animal taxa where they exist (Figure 2; [47-52]). We hypothesize a major duplication event that likely coincides with the first major whole genome duplication event in the common ancestor of vertebrates [35] that produced the UPK2 and UPK3 split. Within UPK2 genes another duplication event occurred to produce UPK2a and UPK2b. This duplication could have occurred in the common ancestor of cartilaginous and bony fish since we found UPK2b first appeared in cartilaginous fish. Alternatively, since in lower vertebrates we have only the genome of lampreys, we could not rule out the possibility that UPK2a was duplicated in the common ancestor of vertebrates followed by the subsequent loss of UPK2b in lampreys (Figure 2).

Since UPK3 has evolved into several gene families the duplication history of this group of genes is even more complex. The appearance of UPK3c could be explained by a duplication of UPK3b that took place in the common ancestor of reptiles and mammals (Figure 2). We hypothesize a duplication either in the common ancestor of vertebrates or in the common ancestor of cartilaginous and bony fish to produce the protoUPK3b and the fish UPK3d genes. Also, some phylum specific upk3b duplication occurred in amphibians (*Xenopus* UPK3b.1 and 3b.2) and in lampreys (UPK3a.1, 3a.3). Overall, we conclude that the evolution of UPK3 family of genes requires at least 4 rounds of duplication to explain the current distribution of genes in the genomes of vertebrates.

### Using phylogenetic congruence to unravel the patterns of coevolution of uroplakin tetraspanin (UPK1a and UPK1b) and the UPK2/UPK3 superfamilies

Phylogenetic analysis of interacting proteins provides a powerful means to unravel the patterns of their coevolution [53-57]. Most studies of coevolution of proteins (thus their genes) take either a tree-based or a distance-based approach [53-55]. The basic idea with these studies is that if two proteins are coevolving and one incurs a mutational change in amino acid sequence, then the other will compensate with mutational change in sites that interact with the initial change. Such changes result in correlated evolutionary patterns both in distances and in phylogenetic relationships. In this study, we take a tree-based approach that compares the likelihood of the topologies of each interacting protein in the pairs of uroplakins. The Shimodara Hasegawa (SH) test allows for such comparison using a likelihood ratio test and enables us to show whether two proteins are indeed sharing strong phylogenetic signal. We suggest that strong congruence of phylogenetic signal is reasonable evidence of the coevolution of two uroplakins. More importantly, the lack of phylogenetic congruence of two uroplakins is strong evidence that they are not coevolving.

**Figure 4:**
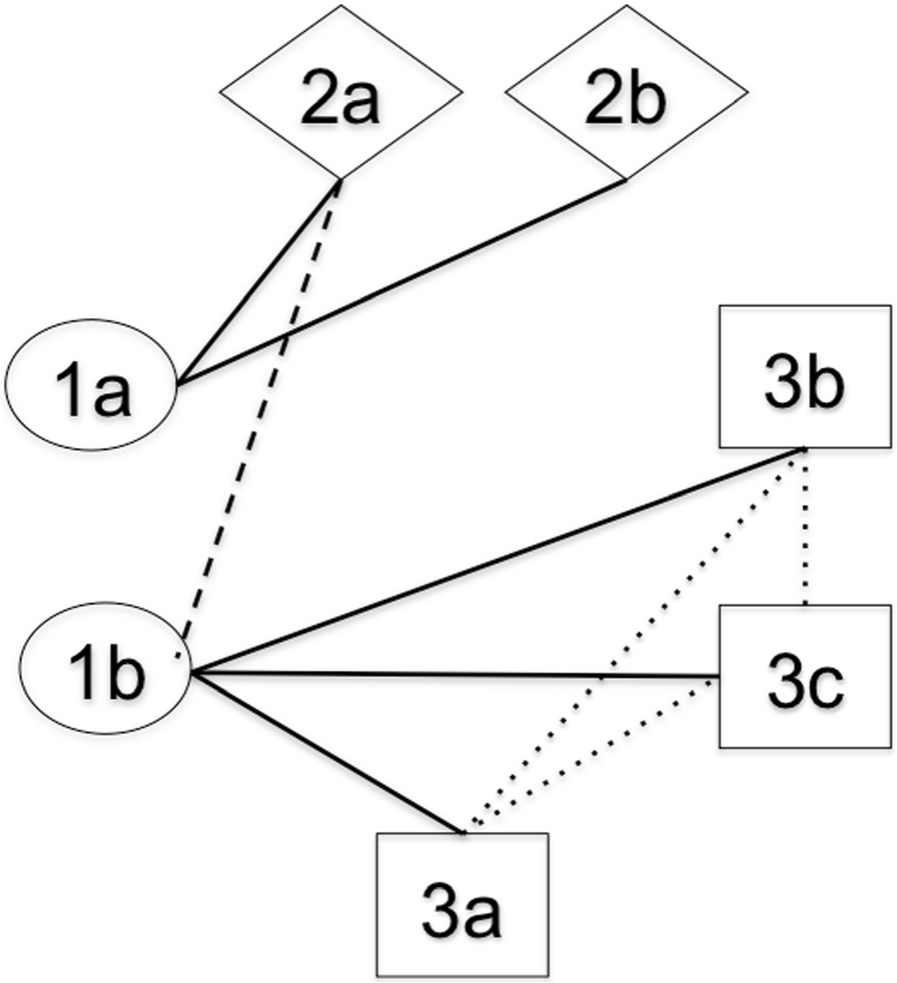
**Uroplakin coevolution.** The figure depicts the results of the SH congruence tests described in the text. A solid line indicates a statistically significant level of congruence (p < 0.01). A dashed line indicates a significant level of congruence (p < 0.05) that is anomalous and discussed in the text. The dotted lines indicate significant results for the SH test (P < 0.05) but these can be attributed to sequence similarity as a result of recent divergence. Abbreviations: 2a = *UPK2a*; 2b = *UPK2b*; 1a = *UPK1a*; 1b = *UPK1b*;3a = *UPK3a*; 3b = *UPK3b*; 3c = *UPK3c*.

Figure 4 shows the results of doing pairwise SH tests on all possible pairs of uroplakins. The figure demonstrates strong congruence of UPK1a with UPK2a, and of UPK1b with all three UPK3’s. These results indicate a pattern of coevolution of UPK1a with UPK2’s and UPK1b with the UPK3’s. The only departure from this general pattern is the unexpected detection of congruence of UPK1b with UPK2a. As we point out before while UPK1b has experienced neofunctionalization in the common ancestor of mammals, it has not been extreme and this fact may influence its freedom to interact with other proteins other than its most frequently observed partner (UPK3). Our phylogenetic congruence test results confirm the structural and molecular experimental data where a strong association of UPK1a with UPK2a and a strong association of UPK1b with UPK3 exists [14,16,58].

## Conclusions

We studied the evolution of genes encoding the two major types of uroplakins, i.e., the UPK1a/1b tetraspanin type and the UPK2/3 tetraspanin-associated type. The tetraspanin UPKs show a clear pattern of duplication in the common ancestor of vertebrates more than likely commensurate with the major genome duplication event that has been hypothesized in this ancestor [34]. Once the duplication occurred in the common ancestor of vertebrates, both UPK1a and UPK1b diverged dramatically as is evident by the different patterns of dN/dS ratios for these two paralog groups. On the other hand, the UPK2/UPK3 group of uroplakins experienced more complex and lineage-specific rounds of duplication to produce the existing genes in these two groups of UPKs. We suggest that UPK2 retained the ancestral function while the UPK3 paralogs neofunctionalized. Again the patterns of skewed dN/dS ratios for these paralog groups support this interpretation.

Moreover, we found that UPK1a and UPK2a show strong congruence with respect to evolutionary history. Likewise UPK1b and UPK3 paralogs show strong congruence, commensurate with their known interactions. Our current work identifies three new UPK families (ortholog groups - UPK2b, UPK3c and UPK3d) all belonging to the UPK2/3 superfamily. Our systematic analysis of uroplakin-related genes pinpoints the appearance of uroplakins to the earliest vertebrates, links the structural diversification and skew in dN/dS ratios with major gene duplication events, and nearly exhaustively identifies all the existing uroplakin families including several novel ones.

## Competing interests

The authors declare no competing interests and are solely responsible for the writing of the paper.

## Authors’ contributions

All four authors conceived the study. RD, JC and AEG collected data and compiled matrices. RD performed the phylogenetic analysis and dN/dS analyses. RD, AEG and T-TS wrote the paper. All authors read and approved the final manuscript.

## Supplementary Material

Additional file 1: Figure S1 List of all uroplakin protein sequences and their accession numbers used in this study. **(A)** Tetraspanin uroplakins (UPK1a and UPK1b) protein sequences. **(B)** UPK2/3 uroplakins (UPK2a, UPK2b, UPK3a, UPK3b, Upk3c and UPK3d) protein sequences exons 2–5. Exons are represented with alternate colors. Aminoacids in red means they are split between two exons (intron phases 1 and 2). For UPK2/3 intron phases are 1,1, 2, 1, 2. http://www.biomedcentral.com/imedia/4692821591035356/supp1.pdf. Click here for file

Additional file 2: Figure S2 List of all uroplakin DNA sequences and their accession numbers used in this study. **(A)** Tetraspanin uroplakins (UPK1a and UPK1b) DNA sequences. **(B)** UPK2/3 uroplakins (UPK2a, UPK2b, UPK3a, UPK3b, Upk3c and UPK3d) DNA sequences exons 2–5. http://www.biomedcentral.com/imedia/1744512341035356/supp2.pdf. Click here for file

Additional file 3: Figure S3 Phylogenetic trees of UPK2/3 and UPK1a/1b DNA and protein sequences generated using parsimony analysis. http://www.biomedcentral.com/imedia/1794860451103535/supp3.pdf. Click here for file

Additional file 4: Figure S4 Phylogenetic trees of UPK2/3 and UPK1a/1b DNA and protein sequences generated using Bayesian analysis. http://www.biomedcentral.com/imedia/9084203271035356/supp4.pdf. Click here for file

Additional file 5: Figure S5. Alignment of UPK3c and UPK3b full protein sequences. Exons are represented with alternate colors. Amino acids in red means they are split between two exons (intron phases 1 and 2). Intron phases are 1,1, 2, 1, 2. Asterisks indicate identical residues, dots indicate CLUSTALW conserved and semi-conserved substitutions. Highlighted in green transmembrane domains. Red box shared ~12 amino acid stretch between UPK3 uroplakins. Human UPK3c GenBank accession number KF150200. http://www.biomedcentral.com/imedia/1013243786103535/supp5.pdf. Click here for file
