# Supplementary material for: Generation of divergent uroplakin tetraspanins and their partners during vertebrate evolution: identification of novel uroplakins
Source: BMC Evol Biol. 2014 Jan 23;14:13. doi: 10.1186/1471-2148-14-13 (PMC3922775; doi:10.1186/1471-2148-14-13)
Supplement: Additional file 1: Figure S1 — List of all uroplakin protein sequences and their accession numbers used in this study. (A) Tetraspanin uroplakins (UPK1a and UPK1b) protein sequences. (B) UPK2/3 uroplakins (UPK2a, UPK2b, UPK3a, UPK3b, Upk3c and UPK3d) protein sequences exons 2–5. Exons are represented with alternate colors. Aminoacids in red means they are split between two exons (intron phases 1 and 2). For UPK2/3 intron phases are 1,1, 2, 1, 2. http://www.biomedcentral.com/imedia/4692821591035356/supp1.pdf. [file 1471-2148-14-13-S1.pdf]

A.Teraspanin uroplakins (UPK1a and UPK1b)

>UPK1a.human. NP_008931.1
MASAAAAEAEKGSPVVVGLLVVGNIIILLSGLSLFAETIWVTADQYRVYPLMGVSGKDDVFAGAW
IAIFCGFSFFMVASFGVGAALCRRRSMVLTYLVLMLIVYIFECASCITSYTHRDYMVSNPSLITK
QMLTFYSADTDQGQELTRLWDRVMIEQECCGTSGPMDWVNFTSAFRAATPEVVFPWPPLCCRRTG
NFIPLNEEGCRLGHMDYLFTKGCFEHIGHAIDSYTWGISWFGFAILMWTLPVMLIAMYFYTML

>UPK1a.mouse NP_081091.1
MASAATEGEKGSPVVVGLLVVGNIIILLSGLALFAETVWVTADQYRVYPLMGVSGKDDVFAGAWI
AIFCGFSFFVVASFGVGAALCRRRYMILTYLLLMLIVYIFECASCITSYTHRDYMVSNPSLITKQ
MLTYYSADTDQGQELTRLWDRIMIEQECCGTSGPMDWVNYTSAFRAATPEVVFPWPPLCCRRTGN
FIPINEDGCRVGHMDYLFTKGCFEHIGHAIDSYTWGISWFGFAILMWTLPVMLIAMYFYTTL

>UPK1a.cow NP_788784.1
MASAAAATTEKGSPVVVGLLVMGNIIILLSGLALFAETVWVTADQYRIYPLMGVSGKDDVFAGAW
IAIFCGFSFFVVASFGVGAALCRRRSMILTYLILMLIIYIFECASCITSYTHRDYMVSNPSLITK
QMLTFYSADSNQGRELTRLWDRIMIEQECCGTSGPMDWVNFTSAFRATTPEVVFPWPPLCCRRTG
NFIPVNEEGCRLGHLDYLFTKGCFEHIGHAIDSYTWGISWFGFAILMWTLPVMLIAMYFYTTL

>UPK1a.opossum XP_001370098.1
MAGEKGSPTVVGLLVLGNIIILMSGLALFAETVWVTADQYRVYPLLGVSGKDDVFAGAWIAIFTG
FSFFVVASFGIGAVLCHSRGMLLTYLLLMLIVYVFECASSITSYTHRDYVVSNPALITKQMLTYY
NAPTSQGQELTRFWDRVMIEQQCCGTSGPLDWVNFTSAFREATPEVVFPWPPLCCRRDANFNPLN
EDGCRLGHQDYLFTKGCFEHIGHAIDSYTWGISWFGFAILMWTLPVMLLAMYYYTIL

>UPK1a.aligator profiling AKHW01101299.1| AKHW01101300.1
scaffold-12507_11
MAEKGNPLVVGLLILGNLIILLAGLALYAETIWVTADQYKVYPILGVSGKDDVYAGAWIAIFCGF
AFFCLGVLGIIALARGSRPLLMVYLALMVIVYIFESASCITSYTHRDFVVSNPRLVTKQMLSFYA
APSRQGRELTRMWDRIMMEQQCCGTSGPLDWVNNTSAFRARFPEVAAPWPFLCCRRDPNFVILNQ
EGCRVGHVDYVNTKGCFEHIENAVNSYTWGISWFGFAILMLACPVMLLGMYHYTTL

>UPK1a.lizard XP_003225012 gi|294743809|gb |AAWZ02022979.1|
cont2.22978
MAAKGSTLVVTLLVMGNVIIMLAGLALYAESIWVTADPYKVYPIMGVSGKDDVYAGAWISIFTGF
SFFCVCVFGIISLVNGNRLMVLLYLVLILVVYLFECASCITSYTHRDFVVSNSRLITKQMLSYYS
EDSYQGRELTRMWNRFMMEQQCCGTNDPMDWVNYTSVFRRRYPEIVAPWPFFCCKRDRNFIIINE
EGCRLGHVDYINTQGCFEHIKHAVQSYAWGVSWFGFAILMLTVCPVVLLAIYHYTTM

>UPK1a.salamander JK981846.1
MADKGSPGLVVLLILGNVVITMSGLALFAETIWATTDPFKVYPILGVTGKDDVFAGGWIAIFCGF
SFFMLGIYGVLATLRGSRTMVMTYLVLMLIVYIFECASCITSFTHRDYMINSNVVQKQMLQYYTD
SSPQGRELTLLWNRMMMEKQCCGVKGPADWIQYTSTFRNQYTEFVAPWPFFCCIRDANFLPINQE
GCRVGAEGYIYTQGCWDHISNAINSYTWGISWFGFAILMWTLLVMLAEMFYYTKL

>UPK1a.frog profiling gi|268046463|gb|AAMC01106311.1| |267885090|
gb|AAMC01156946.1|
MAEKGSSGMVACIVFGNIVILLSGLALFAETIWATTDPYKVYPILGVTGKDDVFAGGWIAIFCGF
SFFILGVFGILAVQRGSRTMVLTYLVLMMIVYIFECASCITSFTHRDYMINSNVIKGQMLTYYSD
SSTPQGRQITSVWLRMMLEKNCCGVDGPLDWVDYYSYFRQSYNETTAPWPLWCCQRDGNFQILNQ
QGCIVGLSSYVYQQGCAHISNAINSYTWGISWFGFAILMWTMFVMLATMYHYTKM

>UPK1a.coelacanth Ensemble ENSLACP00000006022;
ENSLACT00000006074
MADGKGGSSVGALLIFGNLIILMAGLALFAETIWVNTDEFKVYTFLGVSGKDDV

FAGAWIAIFCGFCFFLLGTFGIFAVLKQSRTMVMTYLILMLIVYIFETASCITSFTHRDYVVSNP
NFLKKQMLQLYTSNTSQGIELTEAWNRVMLEEQCCGVEGPMDWISFSSTYQNSISSPSQTSWPLY
CCKRDKNFIMLSELACLIGHKDFVFSNGCWDFLSFSVNKYTWGVSWFGFAILMWTFVVMCLKMYF
YTII

>UPK1a.zebrafish BE200967; AI396779; EO017774
MGAVTCLMVTVVGLNAIAAAAGLALSAVAIWVAVDGYKLYPISGVSGKDDIFAGAWIAIFTGFAF
FLTCIFGIFAALKRSRALMIYLIIMFIIFLFESASAITSATNRDYLVGNSNLVKKQMLQYYADSS
TQGQQITMTWNNVMTQVQCCGADGPTDWIQYNSTYRQLFGAASLWPLGCCKRQSSNFEVVDPIGC
KAGVTSSMFTQGCFQYIESVLSRYTWAVSWYGFSVLMLVFFTLVIAMIYYTQL

>UPK1a.salmo profiling gi|354428581|gbAGKD01030455.1
Contig_030458.
MANGKGFLILGNIFGAAAGLALCALAIWVAVEQYKLYPIAGVSGKDDIFAGAWIAIFTGFAFFCI
CVFGILAATKKSRALMLTYLILMLIIYIFECASCITAVTHRDLVGNSNLVKKQMLKYYAAEGDSG
SRITLTWNKVMNEVECCGTDGPVDWIQYNSTFREKFGTDYPWPIHCCKRKNNYQVVNVKACKNGQ
NTTMFTKGCFNHIESVFSLYTWAISWYGFAVLMFM

>UPK1A.CatFish. CK412483
MVVVVILNVVAAAAGLALCALAIWVAVDPYKVYPISAVSGKDDIFAAAWIAIFTGFAYFCTAIFG
IYAALKRKRSLVLLYLILMFIIFIFECASCITAVTNRDYLIGNSNLVKNQMLKYYAQDSNQGRQI
TGTWNKVMNDAQCCGTDSPMDWIEYNSTFKQTYGSTYTWPLNCCQRQNSFDPADPVGCMFGQTSA
VFSKGCFNYIQTVLNRYTWSVSWYGFAVQMFVFFLLLITIVYFLLLE

>UPK1A.Carp EC392385.1, AU301724
MGAGALTCLMFVVVALNAIAAAAGLALFAVAIWVAVDGYKLYPISGVSGKDDIFAAAWIAIFTGF
AFFLTCIFGIFAALKRSRALMMVYLIIMFIIFLFECASAITAATNRDYLVGNSNLVKKQMVQYYG
QDSIQGKQITQTWNRVMEQVECCGADSPEDWILYNSTFKQIYRTYNWPLSCCKRLSSFELEDPAG
CKVGLTSPVFTKGWFNYIESVLSRYTWAVSR

>UPK1a.spottedgar profiling gi|363782398| gb|AHAT01019516.1|
contig019516,
MSEGRGSTLMMALLVAGNLFLALCGLALYAVAIWVATDGYRLYPLSAVSGKDDIFAGSWIAIFTG
FAFFCAAVYGVFAALRESRAMMLLYLVLMLVIYIFEAASAITAATHRDYLVGNSNLIKKQMLTYY
ADDSDPGRQVTTTWNRVNCCGTDGPLDWISYNSTFRSKFPTQEYPWPLNCCKRKDNFEVLNLDAC
RIGDWNYMNYKGCFDHIEFVFNQYAWAICWYGFAVLMFVLPLMLLAMVYYLKL

>UPK1a.shark EB688237.1; ES415544.1; CV889144.1|
MGETSASPVLKSVLIFGKLVLMLAGIALFAETIWVVTDQFQVYPVLGASGKDDVFAGAWIAIFVG
FAFFCLAVFGILAVLSESRTMVITYLALMLVVYIFECASCITSITHRDYLTSNPKFIKKQMLQFY
GDTSSNGGRDLTTMWNHVMPKEQCCGSTGPADWIQYTSFFRTMYNETFAPWPFQCCKRNANSQNH
QAACAVGHKDFL

>UPK1a.lamprey EE740596; UPK1aTRACE name:PMAH-aab87f10.g1;
Ensemble scaffold: Pmarinus_7.0:GL480986:-1269:28785:
MAKESNAGCVQWLVIIGNVIILLCGITLTAETIWVVTDGYKVYPILGMAHNDDVFAGAWIAIFTG
MAFFLLGFVGIVAALRMTRKLLLGYIVCMLIVFAFESASCITSFTQRDYLVGDQNFLLKQMLTEY
PSDTYPAYTDTWNMFMREQKCCGSNGPTDWLVYTSKFSEAHNNDDVNFPWPMHCCVLSKDGPPLN
ITYCRLGMDGYVNTAGCFDYFSAAVNRYTWGVAWSDSHLCFTFFVLSGAMYLYTVA

>UPK1a2.lamprey EG022567, profiling Ensemble
ENSPMAT00000009881;scaffold:Pmarinus_7.0:GL476495:371924:395954:-
1
LCGMALMATAIWVVTDPYKIYPFLAADNNTDIFAAAWIAIFCGFAFFLLGIFGMYAVWKMQRSAL
LAYFILMLIVFIFEAASCIVIFTHRDYLVGTKNLMLKHMLRDYGEIPSFTDNWKDLMQKCCGVNG
PEDWISYTSSFSIYHKEDDADSPWPTKCCVLNENDDYLYGRMGCILGKPDAIFQKGCYGQFSSAV
DGYTFPLAWFGFSILVLFFVEIGTLYLYTVS

>UPK1b.human. NP_008883.2
MAKDNSTVRCFQGLLIFGNVIIGCCGIALTAECIFFVSDQHSLYPLLEATDNDDIYGAAWIGIFV
GICLFCLSVLGIVGIMKSSRKILLAYFILMFIVYAFEVASCITAATQQDFFTPNLFLKQMLERYQ

NNSPPNNDDQWKNNGVTKTWDRLMLQDNCCGVNGPSDWQKYTSAFRTENNDADYPWPRQCCVMNN
LKEPLNLEACKLGVPGFYHNQGCYELISGPMNRHAWGVAWFGFAILCWTFWVLLGTMFYWSRIEY

>UPK1b.mouse. NM_178924
MAKDDSTVRCFQGLLIFGNVIIGMCSIALMAECIFFVSDQNSLYPLLEATNNDDIYAAAWIGMSV
GICLFCLSVLGIVGIMKSNRKILLVYFILMFIVYAFEVASCITAATQRDFFTPNLFLKQMLERYQ
NNSPPNNDDQWKNNGVTKTWDRLMLQDNCCGVNGPSDWQKYTSAFRTENSDADYPWPRQCCVMNS
LKEPLNLDACKLGVPGYYHSHGCYELISGPMNRHAWGVAWFGFAILCWTFWVLLGTMFYWSRIDY

>UPK1b.cow NP_776907.2
MAKDDSTVRCFQGLLIFGNVIIGMCSIALMAECIFFVSDQNSLYPLLEATNNDDIYAAAWIGMFV
GICLFCLSVLGIVGIMKSNRKILLVYFILMFIVYAFEVASCITAATQRDFFTPNLFLKQMLERYQ
NNSPPNNDDQWKNNGVTKTWDRLMLQDNCCGVNGPSDWQKYTSAFRTENSDADYPWPRQCCVMNS
LKEPLNLDACKLGVPGYYHSQGCYELISGPMNRHAWGVAWFGFAILCWTFWVLLGTMFYWSRIDY

>UPK1b.opossum XP_001363056.1
MAKGDSTVRCFQGLLIFGNVIIGMCGIALTAECIFFVSDQHSLYPLLEATDNDDIYGAAWIGLFV
GICLFCLSVLGIVGIMKSSRKILLAYFILMFIVYAFEVASSITAAVQRDFFTTNLFLRQMLERYQ
NKSQSSNDDIWKNMAVTETWDRLMLQEKCCGVNGPSDWQKYKSAFRDENNDADYPWPHQCCVMDN
LYKPLNLDACKLGVSGYYHSKGCYKLISGPMNRHAWGVAWFGFAILCWTFCVLLGTMFYWSRIEY

>UPK1b.gallus XP_416567.3; DR426684.1; BU224014.1
MAKTDNGIRICQGLLILGNVVIGMCGIALTAECIFFVSDPHGLYPLLEATENNDIYAAAWIGIFV
GFALLALSILGIVGVMKSSKTLLLVYIILMLITYAFEMASCITAATHRDFLTPNLFLKQMLERYM
KSDTDNNNDKQMTEGVTKTWDNLMLQNQCCGVHGPLDWQEYTSAFRMTHNDADYPWPHNCCVMNT
RSEPINLDGCKLGVPGFYNSNGCYDAISGPLNRQAWGVAWFGFAILCWTFCVLLGTMFYWSRIAY

>UPK1b.lizard FG709884, FG750007; gb|AAWZ02001347.1|este exones 3-7; gb|AAWZ02001348.1| FIRST EXON chromosome 3 cont2.1347,
MVKGDTGVRCFQGLLIMGVLGCCGLALMAECIYFVSDQHSLYPLLEATDNDDIFGAAWIGIFTGF
CFFCLSVLGIVGVVKSNRTMLLVYILLMMVVFAFEVASSITAVVHRDFLTPNLFLKQMLEKYQNP
NPINNDDRWKSDGITRTWNRLMVLDDCCGVTGPRDWQNYTSVFRTVSNDADFPWPRQCCVIDVLG
KPTNLDGCKLGVSGYYHEMGCYDTISGPMNRHAWGVAWYGFAVLCWATWVLLCTMFYWSRIEY

>UPK1b.turtle Ensemple. ENSPSIP00000002711; gb| AGCU01013879.1| scaffold639_322,
MAKGDNGVRFFQALLILGNVIIGMCGIALTAECIFFVSDQQTLYPLLEATNNDDIYGAAWIGIFV
GFALFNLSVLGIVGVIKSNRTMLLAYIILMLITYGFEVASCITAATHRDFFTPNLFLRQMLEKYQ
NPEPASNDDKWMSEGVTRTWDRLMLQNQCCGVNGPSDWQNYMSVFRKGNSDSEFPWPRQCCVMDV
QGIPINLDGCKLGVAGYYNNKGCYDIISGPMNRHAWGVAWFGFAILCWTFWVLICTMFYWSRIEY

>UPK1b.aligator profiling 397239340|gb|AKHW01070659.1| scaffold-8202_6,
MAKGDNGVRCFQGLLVLGNVIIGLCGIALTAECIFFVSDQQTLYPLLEATNNDDIYGAAWIGIFV
GFALFNLAVLGIVGVIKSNRAMLLAYIILMLITYAFETASCITAATHRDFFTRNLFLKQMLEKYQ
NPEPLNNDDKWMSEGVTRTWDRLMLQHECCGVNGPSDWQNYMSKFRNINSDSEFPWPRQCCVMNV
QGIPVNLDGCKLGVSGYYNDKGCYELIAGPMDRHAWGVAWFGFAILCWTFWVLLCTMFYWSRIEY

>UPK1b.frog. NP_001037968.1|
MKEDSGVRCYQSIIIFGNVVLGLCGVALTAECIFFVSDQSGIYPLLEATNNADIFAAAWIGIFTG
FCFFILSIVGIIGIMKSNRRMLMVYLILMFVVYAFEVASAITAATQENFFIPNLFLKQMLDFYQN
PNPTNNDNLWKINGVTNTWNRFMLLNGCCGVNGPQDWQTYTSVFRQSNSDSAYPWPQQCCAMNSL
GQPVNLDACKLGVSPYVNLNGCYDQMAGPMTRHAWGVAWFGFSILCWTFWVLLGSMLYWTRIEY

>UPK1b.salamander JK975385 CN045036
MAKDAGVQFFQGLLIFGNVVIAMCGIALTAECIFFVSDQSSLYPLLEATDNDDIFGAAWIGIFTG
FCLFVLSIVGIVGVMKSNRRMLLVYLILMFIVYCFEVASSITAATQRDFFIPNLFLRQMLELYQN
PNPVNNDQAWKINGVTATWNRLMLLNNCCGVNGPTDWQAYASSFRTVNSDSDFPWPRQCCVMNDL
GQPENIDGCKLGVSGHYYNTGCYDLIAGPLMRHAWGVAWFGFAILCWTFWVLLGSMFY

>UPK1b.coelacanth Ensemble ENSLACP00000016648;
ENSLACT00000016762MALKGSSPVRCFQGLLIFGNVVIALTGLTLFAACIFFLSDDNRYYRL
LDASENDDIFAAAWISLFVGFSFFLLSILGIIGVMKSNRTMLLVYIILMLVVYCFEVASCITAIT
HRDFFIPNMFLKTLLEKYQKPKARDLDEQGKIDGVTTIWNRIMPQHKCCGVNGPMDWQEYNSSFR
MQNSDADFPWPRQCCVRAINGEPQNLDACKLGIVGSYYAQGCFEFIAGPLNRHAWGVAWYGFAIL
CWVFWVLAVTMFYFSRIDSTE

>UPK1b.trout FP322998, CU068699
MTPDLDTKDRCFRALLIQGNLVIACCGIALMAMCIFFISDRAGLYVLVYATGNDSIWRGAWIGLF
TGFALFCTSIFGMHAIVVSKRNILLAYILLMVIIYAFEVASAITAATHKDWFVPELFLKQMLQNY
NKPLPDNLPSTQDQIYVINGITEAWNRFMTESKCCGVYGPEDWVKYESHFRQQNTDADNPWPRQC
CQQDAMGAISHLEACKIGVSPFLHAQGCYDYIAGPLITHGFGVSWFGFAILCWTFFVILGVIFHY
TQLDI

>UPK1b.salmo NP_001134735.1; EG792520; EG874850; gi|354302482
|gb|AGKD01156046.1| Contig_156071; gi|354418425
|gb|AGKD01040611.1
Contig_040615,MTPDLDTKDRCFRALIIWGNLVIACCGIALMAMCIFFISDRGGLYVLVYAT
GNDSIWRGAWIGLFTGFALFCTSILGMHAIVVSKRNILLAYILLMVIIYAFEVASAITAATHKDW
FVPDLFLKQMLQNYNKPLPDNLPSTQDQIYVINRVTETWNRFMTESKCCGVYGPEDWVKYESHFR
QQNTDADYPWPRQCCQQDAMGAISHLDACKIGVSPFLHAQGCYDYIAGPLIRHGFGVSWFGFAIL
CWTFFVILGVIFHYTQLDF

>UPK1b.spottedgar profiling gi|363798646| gb|AHAT01003268.1|
contig003268,
MVAKSDIGVRCFQAFLAWGNFCCGLALMAMCIFFIFDRDHLYVLVYATGNDSIWRAAWIGLFTGF
ALFCTAVFGMYAVLKSKRGLLLVYILLMIFIFAFEVASAITAATHKDWFVPNLFLKQMLENYNKP
LPQNLPSTQDEIYKNSGITSTWNMIMTEYQCCGVYGPQDWLQYNSQFRQQNSDSEYPWPRQCCLQ
DGTGGIVNVNACKIGVEPYLFTQGCYDYIAGPLIRQGLGVSWFGFAILCWTFFVVIGVMFYYTQL
DSZ

>UPK1B.skate CV547311;.CO049535; CO050503; AESE010043837 exons 1-
5 gi|363355253|gb|.1| LER_WGS_1_CONTIG_4389;
gi|363324303|gb|AESE010074792.1| CONTING EXON 6
LER_WGS_1_CONTIG_74854.
MSKSTGVRVVQGLLIFGNVVIMLSGLALTAECIFHVSDQHNLWPFLAAADNSDIFAAAWIGLFAG
FCLFCLSIVGIFGIMKSAKKVILTYLILMLIVYIFEVASCITAATQRDFFVPNFFLKQMLQLYGN
PNPLTEAEIRNTFGVTNAWNRVMPDNDCCGVNGPRDWVQHNSAFRVVNSDSRFPWPRQCCTTDQF
GRMLNVTGCKLGIPGYVYE

>UPK1b.Lamprey FD710561; EB084275 1220567368gi; TRACE gnl|ti
|1470569568 reverse exon4 name:PMAC-bnw68a06.g1 mate: 1470753301;
gnl|ti|1423504665 name:PMAC-axy03h10.g1
7MRDVSSGVRCVQILLIVGNVVLLGCGLALFAVSVFVVADPARLWPLLSAFNNTDIFAGAWISIF
TGFTFFLLAMLGIYAVMRLARGLLLAYILLTLFVYIFEVASCITAITHRDFLTKNAYLQQMLQLY
QNPNWVDENSRFNYEGVTAVWDRTMFNYQCCGVNGPQDWQAYGSAFREAHPEGDFPWPLQCCSLD
PASGLIRDLDACRLGLAGYLNTPGCYDYVSGPLHRAAWGVAWFGFAILCWEFWVLLGTMYLYNIS

>UPK1a.platypus exons 2-6 but exon 5 coo intron Boundary CT instead of GT; missing exons 1 and 7; profiling AAPN01309122.1 plus AAPN01141210.1 Cont44002.2,
LSGLALFAESVWVASDPYRVYPTLGVSGKDDVFAGAWISIFTGFAFFLVGSLGLVALLRRSRPMV
LTYLVLMLIVYVFESASSITAHTHRDYMVSDPSLITKEMLTHYGAPSRQGRELTRLWDSIMVEKE
CCGTLGPLDWVHYTSTFRTSIPEAVFPWPPPCCRRDPNFIPLSEEGCRVGHRDYIFTKGCFEHIG
HAIDSYAWGVSWFGFAILMWT

>UPK1b.platypus Exon 3 and 6 missing 24 and 28 aa; profiling
AAPN01272338.1 reverse exons 1,2 | AAPN01106002.1|exon 3
AAPN01297767.1 exon4 AAPN01302484.1 exon 5 AAPN01104274.1 exon
Cont11876.1.

MAKDDGSVRCLQGLLVFGNVVIAMSGLALMAECIFFVTDPWRLYGLLEATDNDDVFAAAWIGIFT
GFSLFCLAALGVVAVLRAGRKLLLAXXXXXXXXXXXXXXXXXXXXXXXXXFTPNLFLRQMLERYQ
NQSGPTNDDQYKNKKVTDTWDRLMLQEKCCGVNGPSDWQDYHSAFRRTHLDADFPWPHQCCARDG
QYRPLSLDGCKLGVADYYHEEXXXXXXXXXXXXXXXXXXXXXXXXXXXXFCVLLGVMFYWSRIEP

>UPK1a.dog XP_541693.1
MASAATEAEKGSPVVVGLLVVGNIIILLSGLALFAETVWVTADQYRVYPLMGVSGKDDVFAGAWI
AIFCGFSFFVVASLGVGAALCRRRSMIVTYLVLMLIVYIFECASCITSYTHRDYMVSNPSLITKQ
MLTFYSADTDQGQELTRLWDRIMIEQECCGTSGPMDWVNFTSAFRTATPEVVFPWPPLCCRRNGN
FIPLNEEGCRLGHTDYLFTEGCFEHIGHAIDSYTWGISWFGFAILMWTLPVMLIAMYFYTTL

>UPK1b.dog XP_850140.2
MAKDDSTVRCFQGLLIFGNVIVGMCGIALMAECIFFVSDQHSLYPLLEATDNDDIYGAAWIGMFV
GICLFCLSVLGIVGIMKSSRKILLAYFILMFIVYGFEVASCITAATQRDFFTPNLFLKQMLERYQ
NSSPPNNDDQWKNNGVTKTWDRLMLQDHCCGVNGPSDWQKYTSAFRTENNDADYPWPRQCCVMNN
LQEPLNLEACKLGVPGYYHKQGCYELISGPMNRHAWGVAWFGFAILCWTFWVLLGTMFYWSRIEY

>UPK1a.elephant XP_003420803.1
MASAAAEVKKGSPVVVGLLVVGNIIILLSGLALFAETVWVTADQYHVYPLMGVSGKDDVFAGAWI
AIFCGFSFFVVASFGVGAALCRRRSMILTYLVLMLTVYVFECASCITSYTHRDFMVSTPSLITKQ
MLTFYGADTDQGQELTRLWDRIMIEQECCGNSGPMDWVNFTSTFRKATPEVVFPWPPLCCRRNGN
FIPLSEEGCRVGHVDYLFTKGCFEHIGHAINSYTWGISWFGFAILMWTLPVMLIAMYFYTTL

>UPK1b.elephant XP_003413030.1
MAKDNSTVRCFQGVLIFGNVIIGMCSIALMAECIFFVSDQHSLYPLLEATNNDDIYGAAWIGMFV
GICLFCLSILGIVGIMKSNRKILLAYFILMFIAYGFEVASCITAATQRDFFTTNLFLKQMLERYQ
NNSPPNNDDKWKNNGVTKTWDRLMLQDNCCGVNGPSDWQKYTSAFRIENNDADYPWPRQCCVTNN
LKEPLNVEACKLGVPGYYHSQGCYELISGPMDRHAWGVAWFGFAILCWTFWVLLGAMFYWSRIEY

B. UPK2/3 uroplakins (UPK2a, UPK2b, UPK3a, UPK3b, UPK3c AND UPK3d). Exons 2-5

>UPK3a.human NP_008884.1
AVNLQPQLASVTFATNNPTLTTVALEKPLCMFDSKEALTGTHEVYLYVLVDSAISRNASVQDSTNTPL
GSTFLQTEGGRTGPYKAVAFDLIPCSDLPSLDAIGDVSKASQILNAYLVRVGANGTCLWDPNFQGLCN
APLSAATEYRFKYVLVNMSTGLVEDQTLWSDPIRTNQLTPYSTIDTWPGRRSGGMIVITSILGSLPFF
LLVGFAGAIALSLV

>UPK3a.mouse NP_075967.2
TVNLQPQLASVTFATNNPTLTTVALEKPLCMFDSSEPLSGSYEVYLYAMVDSAMSRNVSVQDSAGVPL
STTFRQTQGGRSGPYKAAAFDLTPCGDLPSLDAVGDVTQASEILNAYLVRVGNNGTCFWDPNFQGLCN
PPLTAATEYRFKYVLVNMSTGLVQDQTLWSDPIWTNRPIPYSAIDTWPGRRSGGMIVITSILGSLPFF
LLVGFAGAIILSFV

>UPK3a.cow NP_777134.1
GVNLQPQLASVTFATNNPTLTTVALEKPLCMFDSSAALHGTYEVYLYVLVDSASFRNASVQDSTKTPL
SSTFQQTQGGRTGPYKAAAFDLTPCSDSPSLDAVRDVSRASEILNAYLIRVGTNGTCLLDPNFQGLCN
PPLSAATEYRFKYVLVNMSSGLVQDQTLWSDPIRTDRLTLYSAIDTWPGRRSGGMIVITSILGSLPFF
LLIGFAGAIVLSLV

>UPK3a.elephant XP_003420946.1

AVNLQPQLASVTFTTNNPTLTTVALEKPLCMFESNTALVGTYEVYLYVLAESASLRNASIQDHTSAPL
SSTFQQTEGGRTGPYKAAAFDLQPCSDLPSLDAVEDVSRASEILSTYLVRVGANGTCLSDPNFQGLCN
PPLSVATEYRFKYVLVNMSTGLVQDQTLWSDPIRTNRLTPYSAIDTWPGRRSGGMIVITAILGSLPFF
LLLGFAGAVLLSFV

>UPK3a.dog XP_851414.1
GVNLQPQLASVTFATNNPTLTTVALEKPLCMFDSAATLNGTYEIYLYVLVNLASSRNASVQDGARAPL
SSTVQQTEGGRTGPYKAVAFGLIPCSDLPSLDAVGDVARASEILNAYLVRVGANGTCLSDPNFGGLCN
APLSAATEYRFKYVLVNMSTGLVQDQTLWSDPIRTNRPTPYAAIDTWPGRRSGGMIVITSILGSLPFF
LLVAFAGAVVLSLL

>UPK3a.opossum XP_001378435.2
AVDLEPQMASITFATNNPTLTTITLEKPFCMFNASNLGNVSYEVNLYVMENSGSVRYAVIKNNRSFPI
NSTFQETAGGQRAPYKAASFILPQCGDLPNLDEAGDVSKVAEILSAYLVRVGDNGYCSLNPNFKGTCN
PPLTRATEYRFKYVLINPSSGFVEDQTLWSQPIRTNQISPYLEIDTWPGRRSGAMIVITSILSTLVFF
LLVGFAAAVIFSFV

>UPK3a.platypus profilinf AAPN01155619.1 PLUS
AAPN01135149.1Cont1295.23,
AQIKPQIAGLAFATNNPTLTTIALEKPFCMFDGAGPAQLPSEIYLYVMMDSAADAHPVLDNGSRPLRTT
FQQAAGGRRGPYWAATFSVPRCEDLPQLWDAGDPARAPQILDAYLFRVGGDTACMWDPDFSGACNPPL
AGETGYRFKYVLVNTTSGSVVDQSLWSDPIRTKRTLSWSRVDTWPGRRSGSMIVITSILSSLMFVLLV
GLAAAVTCRV

>UPK3a.chiken BU410839, BU464912 and profiling NW_001471513.1
Gga1_WGA14_2:27108888-27154218
DQSMKPQLAAPELATNNPTLTTVALEKPFCMFDSSLHPNKSYAIYLYVMKSSANTISSVVTDSSSKPL
DSTFQQTHGGHLGPYKAASFDVPNCVSPPRLADAGDINKVSDVLKQYLFRVGDDGTCLYDPNFLDVCN
PPLAPDTTYRFKYVLVDNTEGIVKDQTLWSDPIKTRKAKLPMKIDIWPGRRSGSMIVITSILSVSVFL
LLAGLLASVFSALV

>UPK3a.aligator profiling gb|AKHW01039965.1| scaffold-4401_2,
AQSLRPQIASPQLATNNPTFTTIALEKPFCVFDGSLSPGKSYEVYLYAMMDSMSMISSAVTDNSSKPL
DSTFQEVNGGQLGPYKAAVLNVPDCASPPKLADIRNVKKASDVLKQYLFRVGDDVSCLYDPNFLGVCN
PPLASDTTYRFKYILVDETLGIMKDETLWSDPIKTNSVKKSSTIDTWPGRRSGGMIVITSILSVLIFL
LLAGLFASVFFAVM

>UPK3a.turtle profiling gi|350386162|gb|AGCU01117018.1|
scaffold678_371,
ALNLRPQIANPKLATSNPTLTTIALEKPFCMFDDSLSPGSSYEVYLYAMADSESTVSSAVTDNSSKPL
NTTFQDTNGGQLGPYRAALFNVPNCASPPMLADVVNVKKVSDVLKQYLFRVGDDVTCLYDPNFPGACN
PPLAQDTTYRFKYLLVDVNAGVVKDQTLWSDPMKTRRVKQSSTIDTWPGRRSGGMIVITSILSTLMFI
LVAGFLASLYFIVM

>UPK3a.Xtropicalis BX737880 CX441249 ENSEBLE
scaffold:JGI_4.2:GL173822.1:32034:48169:1
AGADMPLLANSDFFSLNPTQTTIALEQPICMFKSAVNVYLIGIVAGAPNTPLYDGNKKVNASTYSGTQ
GGKTGPYIVAKLPNQQCINIQALSNMADPTQVQSILSKYVVRVGADVTCLTNPNFVGYCNAPLQGNTQ
YSFKYLFTDSGDIVQSETSWSLGITTVNGKASSTIDTWPGRRSGGMIVLTSILSTLMFFVFIAYVIGF
AYSIA

>UPK3a.Xlaevis DC114372, DT404162
ARSAVPLLANSDFFSLNPTQTTITLERPFCMYKDAINVYLFAIVKGATNIQVADAAKKVIASNYTGTQ
GGLLGPYQVAKLDNPKCENIQASNIMADPNKYIVRVGGDVNCLTDPNFKGICNPPLQNNLQYRFTYVF
TIGDVVQYQTDWSPPISTVNVKSSGTIDTWPGRRSGGMIVLTSILSTLMFFVFFAYIVGFAYSIL

>UPK3a.salamander JK978030, JK980634, DT404162.
TFAVTPQVAGPALVPNTPTLNTVALQKPVCLFDSQTGGDPANYQVELFAMAASAPSSTPLASGNTFRN
TSGGTTGPYVAGKFGVPNCTLVGSLTPSQDDFLRQYIFRVGDNPTCLTDPNFSGICNPPLANSTAY**R**F
LYSLVHNNGSRVANTHWSDRISTKNVKTPDTLDTWPGKRSGGMIVITSILSTLLFFLLSGFVAAANVL

>UPK3a.coelacanth Ensemble
scaffold:LatCha1:JH126738.1:234530:268960:-1; gbAFYH01050375
gi|346164461|gb|.1| contig050375.
AQFPKPEIANPQFVAGNPTLTTISLEKPFCVFDRTISASSGDFTVAVFAVKSTVAVAENINDFTQTYQ
SSREGTTAPYKAASFAVPNCDSPLVLSNPVNVNVIRSLLNQYLIRIGDDTMCSNMPGICNGPLTPNTA
YRFKFVLLNGNRPAAQTPWSSMISTRKSKPFEDIDTWPGKRTGGQVVVTTILVILLFLLLCGFVTTLV
ASI

>UPK3a.spottedgar genomic profiling AHAT01033516.1 contig033516
EALKVKPEAVSPRLLRFNPTQSTVSLAKPLCVFDSVKPTNEMMVDVYVVHSLSATLTFETGKTYKETN
GGTETPYKATSFGIPNCTSPPNPADLSVPQRIDKTLDEYLVRIGSNPTCVGEPEAEAFCNAPLSDGTS
YRFKYLLVNGTTTIAETEWSESILTRKALSPDEIDTWIGKRSGGGIVVTVILSLLLFLLLGAAIFMGV
LDVI

>UPK3a.skate EE989195; GH546922; AESE010876980.1WGS_1_CONTIG_884036,
AESE010997029.1;WGS_1_CONTIG_1005178;AESE012684067.1WGS_1_CONTIG_284
8843
PLSFKPEVSSIALTLGLRTSTTVTLAKPICVFSSGDVVEVFGVQTTADSIPIEIGNRTILTYQQTDGG
ARGPYRAARFMTPICTSLPFVPSRDPAVIRVQIEQYLFRVGDDDQCLNQAPFVSGNCNAPLKENVAYR
FKYAVLNSSTNIILNETSWSDPIPLLRVADFALIDTWPGARTGGMVVITTLLVILLSLLLCGYGALLV
YACC

>UPK3a.Shark JK928319; AAVX01399128.1|PLUS AAVX01255679.1
ILQFKPEMPSVSTIDGLRTARTVTLSKPLCVLPTGHMIELLVVQGNVTPIATNLDGTYQATKGGATGP
YRAARFANPECTSSSFTPSTDPTKIQTLIDRYFIRVGSDTQCLSGGTAQSVPCNAPLNENVNYRFKYV
ARDPITFILRDETVWSKPITLLQVQDPARIITWPGARTGGMVVLTTLLVIILFLLLCAFVAFL

>lamprey3a.3 profiling Ensemble scaffold:Pmarinus_7.0:GL480097.
GERYGNLFPSTSSTRCANQFQSFVSSAHSSSLLRLYDTTTERSAPGYSFSRVLGAVGSRVATGAGDRG
PYLAGYFPVPPCAQPPPPSVVVGTGGSSVALALLGMVIFRLGGDPACATGIADDPAALVCNGPLWNGT
AYRVKFVLLARSSLLSPPAMETPWSDPISTKPVVSVEQIHVWPGKRTGGMVVVTSVLCSILFVLLFAL
TAALLAPS

>lamprey3a.1 FD718834.1 FD706071 FD704820; profiling Ensemble
scaffold: Pmarinus_7.0:GL477389.
GSGTKPQLVSSTAVPYNPTETTIVWSKPFCVFQKPVPTTQYVVDVYASITNNSYAFDNSIGAVLSSYW
TNAVSPSPYLAATFKVPDCASQPSIYDAMAVKTNATFRLGGDTACVNSIGPSTSVCNGPLVPGMKYRV
KYTLSEESPQFPRTIVDQTPWSDPVSTKKSPAASTINTWPGKRTGGMVVVTAVLSTLLFLLLAALLLV
VIFKACS

>lamprey3a.2 FD709144 prolfiling Ensemble scaffold:_7.0:GL483426.
VSQVVPTVVNPNLLGAVTQTTVALQAPFCSALDAEVVALSSVSADLRLFVMATAQRNVSNEMITSSST
IGLDKGYAGSGAGTSSWYLALGGRPLQNCTISTPSLSTPTSYYRVGADSKCSVAVTCNGPLNAGTIYW
FKYIIGTVATHGAIDRSYLESSWSKPIRLNKAGELNAIGVTPGPXSGGMVVVTVILVVLLFIAVDRAR
PALGQ

>UPK3b.human NP_872625.1
ELVPYTPQITAWDLEGKVTATTFSLEQPRCVFDGLASASDTVWLVVAFSNASRGFQNPETLADIPASP
QLLTDGHYMTLPLSPDQLPCGDPMAGSGGAPVLRVGHDHGCHQQPFCNAPLPGPGPYRVKFLLMDTRG
SPRAETKWSDPITLHQGKTPGSIDTWPGRRSGSMIVITSILSSLAGLLLLAFLAASTMRF

>UPK3b.cow NP_001012690.1
DLIPYTPRITSWDLEGKVTATTFSLEQPRCVLDRHSSAADTVWLVVAFSNASRVFQNPQTLAEIPASP
RLLTDGHYMTLPLTMDQLPCEDPADGSGRAPVLRVGNDAGCLADLHQPRYCNAPLPGPGPYRVKFLLT
NSRGSPQAETRWSDLIALRQGKSPGSIDTWPGRRSGDMIIITSILSSLAGLLLLAFLAASSVRF

>UPK3b.mouse NP_780518.1|
DLIAYVPQITAWDLEGKITATTFSLEQPRCVFDEHVSTKDTIWLVVAFSNASRDFQNPQTAAKIPTFP
QLLTDGHYMTLPLSLDQLPCEDLTGGSGGVPVLRVGNDFGCYQRPYCNAPLPSQGPYSVKFLVMDAAG
PPKAETKWSNPIYLHQGKNPNSIDTWPGRRSGCMIVITSILSALAGLLLLAFLAASTTRF

```
>UPK3b.elephant XP_003416614.1
DPLHATDNGLGGKVTATTFSLEQPRCVLDGHARATDTVWLVVAFSNASKDFQKPKTQAEIPTFAQLLT
DGHCMTLPPSPVQLPCTDSVGGSGSALLLRVGNDARCLADLQQSPYCNAPLPSPGPYSVKFLLMDTEG
SPKAETRWSDSIALHQGRAPGSIDTWPGRRSGGMIVITAVLSSLAGLLLLAFLAASTVHF

>UPK3b.dog XP_849277.2
ELIPYTPQITAWDLEGKVTATTFSLEQPRCVLDGPASVASTVWLVVTFSNASKDFHNPQTLAEIPAFP
RLLTDGYYMTLPLSLDQLPCEDPEGGGRSIPLLRVGNDPGCLADFYEPPYCNNPLPSPGPYRVKFLLM
DARGSPQAETRWSDPITLHQGKAPGSIDTWPGRRSGDMIIITSILSSLAGLLLLAFLAASTVHF

>UPK3b.platypus profiling gi|125702340|gb|DS191120.1| Scfld14595
LMSYKPQVSAHPLEGKVTASTFTLDQPRCVFDGQVVSTDTIWLVVAFSNASRDFQNPTTAASIPAYPR
LLTDYYYMTLKVSPDLYPCADAGGLSVLRVGTDPGCLRDPGREYCNAPLPAPGPYRTKFLVMDEASHP
KAETQWSDPITLKQGRDPSSVDTWPGRRGGSMVVIASILSVLAGLLLLALLTAAAGACTV

>UPK3b.opossum XP_001378885.2
DQIPYTPQISALALEGKVTAATFSLEQPRCIFSELAAPADAVWLVVAFSNATEDFQNPKTAAEIPSYT
ELSSSFYYMTLKLSPDLYPCEEEDIAVLRVGSDTNCLRNLSQEYCNAPLLAPGPYRVKFLVMDNNGQP
KAETWWSDPITLNQGKDPRSIDTWPGRRSGCMIVITSILSTFAGLLVIAFLIASTVQF

>UPK3b.Xtropicalis EL866947; Ensemble scaffold:JGI_4.2:
GL172708.1:135056:150415:-1
DITSYVPQLTLSPIVGTVTSTTFVLDKPQCVFGNTGNQVWLLVARSNVSANVVLTPPSMYSSFATKGY
YHVPFGTESLYHCSNTAEYIRVGDTAQCNDNTNCNGPLPDPGPYRVKYLVMNNNALVSQSLWSQQITL
LTGKSSSQLDTWPGRRSGGMIVLTSILSVLMGILTLCLFAAFFVGC

>UPK3b.Xlaevis BP703707 BJ034026
DITTYVPQLTLMPIQGSVTSTTFTLDKPQCIFGSRTNQVWLLVARSNVSVSITNAMLKPPSMYSSFPT
QGYYHVPLGTEASYPCSNTADYIRVGDTVYCTDNTYCNAPLPDSGPYRVKFVVMNNNALVSSSLWSGL
ITLRTGKNPSTIDTWPGRRSGGMIVLTSILSLLMGILTLCLIAAFFVGC

>UPK3b1.Xtropicalis profiling Ensemble scaffold:JGI_4.2:
GL172728.1:2871266:2881345:1
IPYYVPQITTKPILGKLTSSSFVLEQPQCIFQQYKTSLVWLVVALNRVIPQLSYTQLSNPANISSFET
NGFYHTLPVFGGDYPCADTSGQLSAMIYVGSDVNCSNPLFCNGPVPSRGTYRVRFVVLNGTVMETGTR
WSEVITMHIAINSSTIKTEPKRPSSGMIVIITILASLLFILLVCLIAAVSLGS

>UPK3b2.Xtropicalis XP_002934406.1; EL802701; Ensemble scaffold:
JGI_4.2:GL172728.1:2884693: 2898749:1
DVGSYVPKITTSILGNLTFSTFVLEQPQCIFSSNYPTQDVWLVVALDTVEPFLTDTNLSTPVTYSSFT
TNKFYHTLRVRGADYPCFNESAMSLALLQVGADEKCNESFCNGPLTSPGPYRVRFVVLNNTGMVAKTN
RSDLIRLPIGINYTTIDTWPLSRSGSMIVITTILSILLAVLLACLLAALCSE

>UPK3b.chiken one extra cysteine; profiling chromosome:
WASHUC2:19:4127260:4157406:-1
ALLPYVPRVAPGAMPGKVTATTFVLERPRCIFDPFANASDAVWLAVAFADA
SAAFKNPTSSAEVPPYEGLPTARAYMTLQMAAAAYGCSAPGAAVLRVGGDTACHGRAPCNGPLPSPGP
YRVKFLLMGCGGPKAETKWSDPILLRRARSLSTIDPTPARRSSTAVVIAAILASLGAALAMAVLGAVG

>UPK3b.aligator profiling gi|397229275|gb|AKHW01080724.1 scaffold-
9559_4
ELVPYTPQITGNSLEGKLTASTFSLDQPICIFDQYVNATDDIWLVVAFANATSSLKNPTSRTDIPPYQ
RLSTALHYXTLRTTIXFFPCPRSTNTSVLRVGSDAFCRNDNSQQHCNGPLPNPGPYRVKFLILDSNGA
KAETRWSERITLKQGHRSSTIDTWPGRRSGTMVVITVILSSLVGILTIAFLCTSAYD

>UPK3d.coelacanth profiling AFYH01004438 gi|346210398|gb|.1|
contig004438.
DVPYLPEVAFNNVAGSVTATTFTLQQPRCIFKDVFSSCPLCQLWVAVATEKGANNLITTVGRPVTFAG
YQKLSQRGYYFTMRSSKDLYKCVENDDKVRVLRLGADTSCPQSIGESDCNGPLPDSGSYRARFLIVDS
QSGNMLKAQSQWSKSIQLKRPKDPASIDTWPGKRTGGMVVVTTILVILLAILLLLFIVALITAC
```

>UPK3d.1.spottedGar genomic profiling AHAT01005644.1 contig005644
VTSVEYTPEITAYNMAGRVTGTTMVMKQPRCYFDNQLLLPCTPDKCEIWLVAAVGSAGIQNFDADKGK
STILSESPYPTAFTGSPPKNYYLTKVGRQQVFPCQQSHGIVYFRVGDEGNCTSANCNGILPAGSTVRV
KYVLVDPASRNVTTETYWSQNITLYSSTDPVVIFDGIRQRSAGMIVITSILSVLLFLLLLLLIAGLIY
AK

>UPK3d.salmo BG936628; AGKD01191862.1| Contig_191888
IVSLDHIPEILPYEASGRVTSTTIVLGQPLCYFNTLTQLKCSQSTCQVWAAIASGPGINNFDIDKLVA
VQIVSASPYPIAFSSQTNRMYFVTKLGRPKDFPCGQLPGIKYFRVGAEGNCTNTNCNGILPPGSTVRV
KYILIDPVSRGVVSESKWSYPISLTSTRSWSSIDEWLGKRSGGMVVITVISSCLLAVLLLLLGAVLLL
DG

>UPK3d.trout CX140558, CA369219, BX073041, CX140559.
IVSLDHIPEILPYEASGRVTSTTIVLGQPLCYFNTLTQLKCSQSTCQVWAAIASGPGINNFDIDKLVA
VQIVSASPYPIAFSSQTNRMYFVTKLGRPKDFPCGQLPGIKYFRVGAEGNCTNTNCNGILPPGSTVRV
KYILIDPVSRGVVSESKWSYPISLTSTRSWSSIDEWIGKHSGGMVVITVISSCLLAVLLLLLGAVLLL
DG

>UPK3d.zebrafish A2CE76; DT080652.
GQIFQPQLAPANFLGRITSNTVILQQPYCVFTQTCPGCEIWLVAALSTGTGNFNALVNISSPISLSVS
PYPTAFLPSSAQFFLTRVGPLANFPCNTAPAFPYFTVGADGICTGINCNGVLPVGSIVSFRYLLIDPS
NYTVVNMTNWGGPFNLTTLLSYQTINDGLSARSGAMVVITTLLCVAVALLLLVFFIMLCVSC

>UPK3b.skate EE991990; AESE010072473.1| _WGS_1_CONTIG_72535;
AESE012558708 WGS_1_CONTIG_2721782; |AESE011082469.1| Leucoraja
erinacea LER_WGS_1_CONTIG_1092019; AESE012622692.1
_WGS_1_CONTIG_2785766; AESE010900346.1 _WGS_1_CONTIG_907428,
AESE012509864.1|WGS_1_CONTIG_2672938
ADEADFVPEIFKDNVIGGVSQTTFALQQPQCVFDRFQPACTLCEIWLVVDNPANVSTFDTNMNTLTPS
AATYKDFLLNGFYLTVKTARTDYACPQTMGQVYTLRVGDEDPCTTPNCNAPLNAGSLVRVRYVMINPL
ATTNNVIAVTKWSNPIQLENAVDPNIIDTSTRRSGAMVVITTILSILLFLLLVLFIVMLAA

>UPK3b.Shark AAVX01026120.1| PLUS TRACE gnl|ti|1573944991 PLUS
AAVX01044278.1|Callorhinchus milii scaf_1099306886782, whole genome
shotgun sequence
VKNYTPEVTNANLAGRITATTITFQQPLCQFDSEINATPGLIFWLVVANSQRGQNTFDAITFVQPSLM
THQTASTADYYLTLRTLAERYLCPPTSSSNGEMYFVRVGQETTCRTETCNVPLNNSKSYSVKFILINP
SLQNSPNVIAQTNWSSFISLKTVVEPRNIDPSPRGRSAGMIVITAILSVLLFLLLAFFVAMLLMVC

>UPK3d1.salmo profiling AGKD01066640.1| Contig_066647.
IPVNNTPEITPSSLAAKLTSNSVILTSPSCYFNGLGNLSCNSTTTCEIWLVSAKDTGVSNYDADNKMP
YIDTRSPYPTAFSSNTSKKYFLTKLGLQKAYPCPIVAGTGYFRVGSDGNCSTPNCNGILPVGSTARFK
YVLINPANKTVVAESLWSNNITLYSLKDPEKIDNGFAGRSAAMIVITAILCSFLALLLLLLLIMLIYV
L

>UPK3d1.trout CX145617, CX153372.
PVNNTPAINPSSLAAKLTINSVILTSPSCYFNSLANLPCNSTTTCELWLVSAIDTGVSNYDADKNRPF
IDTLSPYPTAFSSNTSKKYFLTKLGFQKDYPCPIVAGTDYFRVGSDGSCSTPNCNGILPVGSTARFRY
VLINPENKTVVAESLWSNNITLYPLKDLESIDHGFAGRSASMIVITAILCSFLALLLLLLLIMLIYVL
CC

>UPK3d.Spotted Gar genomic profiling contig005644, Sequence ID:
gb|AHAT01005644.1|
TVPSVDYVPEIIPMKLAGRLTGTTFLLRQPLCYFSNQQGLNCSLSTCEIWLAVAREAGVNNFDTDKVQ
PSFDIVSASPYPEAFQNKNYYVTRLGVQNNFLCAELPGIRYFRVGAEGNCSTPTCNGILPAGSTASCA
VLGLAVVSQLQDQLVKGALLWAQQGLVVVDVMPSSSIDDWTWKRSGGMVVITVITSCLLAILLLLVAA
LLLGG

>UPK3d.2 Spotted Gar genomic profiling |AHAT01005643.1 contig005643

VPYAPEVTPHNLLGRVTSTTLTLQQPVCFFNNLSGLPCAPDNCEIWLVIARGVPKFEAIKGNTSVLMS
SPYPDAFRNNSSPNYFLTKLGVQNAFPCAESTGFRFFRVGDEGLCSTSNCNGILPAGSTVRAKYVLLD
PGSKQVVSESQWSSPISLIALSDSAGIDEWIGKRSGGMIVVTSILSSLLAILLLFLLTAFVLRS

>UPK3c.human NP_001107875
AAPEHISYVPQLSNDTLAGRLTLSTFTLEQPLGQFSSHNISDLDTIWLVVALSNATQSFTAPRTNQDI
PAPANFSQRGYYLTLRANRVLYQTRGQLHVLRVGNDTHCQPTKIGCNHPLPGPGPYRVKFLVMNDEGP
VAETKWSSDTRLQQAQALRAVPGPQSPGTVVIIAILSILLAVLLTVLLAVLIYTC

>UPK3c.mouse XP_485688.3
ESINYAPQLLGATLEGRLTQSTFTLEQPLGQFKNVNLSDPDPIWLVVAHSNAAQNFTAPRKVEDRHAP
ANFDRNGYYLTLRANRVHYKGGQPDSQLRVLRVGNDNNCSLESQGCNSPLPGAGPYRVKFLAMSAEGP
VAETLWSEEIYLQQAQTFREAPGSQGKGTVVIIAFLSILLAILLVVFLVLVISAC

>UPK3c.dog XP_850037.1
EHISYVPQLSNRSLAGTLTQSTFTLEQPRGQFSHRSISDSDAIWLVVAHSNATQNFSAPQRVEDIPVP
EDFTRRGYYLTLMANRLLYPGNQPGNQLRVLRVGNDTSCSPTKRGCNHPLPGPGPYRVKFLVMSDKGP
VAETEWSNETHLQRAERLQAAPGPQSTGTVVIIAILSVLLAVLLTALLALLIYTC

>UPK3c.cow gb|AAI49699.1
ERISYVPQLSSATLAGRLTQSTFTLEQPRGQFSHPSISDSDAIWLVVAHSNATQKFTAPQKVEDTPVP
ADFPQRGYYLTLRASRALYPGGPPSNQLRVLRVGNDTRCSPRTRGCNRPLPGPGPYRVKFLVMSDRGP
MAETEWSSETRLQQAEVLQAAPGPQTAGTVVIIAILSVLLAVLLAALLALLIFTW

>UPK3c.elephant profiling gi|253616662|gb |AAGU03064090.1
cont3.64089.
ELISYVPRLSNATLAGKLTQSTFTLQQPRGRFSHKISDFDAIWLVVAHSNATQSFIAPQRVKDSPAPA
DLPLKGYYLTLRASRALYPGDQAGNQLQVLRVGNDTRCSLTTKGCNHPLPGPGPYRVKFLVLSDDRGL
VAETEWSGETHLQRAEVLQASPGPQTAGTVVIITFLSVLLVVLLTALLALLIYIC

>UPK3c.opossum XP_001378846.2
EPINYTPAITREPLEGSITSSTFTLDQPNDQFNGSGISDLDDIWLVVAFSNASQSFEPPQSAQDIPYA
ATFLDKKYYLTIRASRDLYSSKRGSQGISVLRVGNETNCTRSDCNKPLPGPGPYRVKFLVMNTNGPVA
GTNWSEDITLRKPVEFSESRPPSKSAGTIVIIAILSILLSLLFLALVALLVYTC

>UPK3c.platypus profiling gi|125692247|gb|DS201210.1| Scfld26655
KIAYTPVITKLPMEGKITLSTFTLDQPLGQFNSSAVDDLDDIWLVVAYSNATDNFSNPVKPDEEYNLS
ELSKKQYYMTMRATRDLYPGGNNSSFLHVLRVGQEANCTSKSCNGYLPGPGPYRVKFLVMNERGPVAE
TDWSDNIALREAKDPGDPNPTRRSTFMIVIVTILSVLFALLLGALTGVLIQAC

>UPK3c.Lyzard profiling gb|AAWZ01050405.1| cont1.50404; whole genome shotgun sequence REVERSE
VNYTPRLASENLGGKVTASTFTLDQPRCVFNDVVNATDGIWLLVARSDAARNFTRPGSPSELPFQDLE
KNGLYLTLNTAPASYPCPEPGAAGGPLTVLRVGNEVQCASNRARPDCNGPLPRPGPYRVKFLAINPDG
VTAESEWSEEIALVQA
QSPETIDVSPGRRSASAIAIASLLSILCAVLLAALIAALVYKYT

>UPK3c.chiken profiling ref|NW_001471508.1|Gga19_WGA256_2
DKLSYKPTLVGGNVEGRMTGSTFVLEQPRCVFDSYSTANIWLVVATRAGMNAFNDSAQPGMPEWSFQR
FPTNTSAYLTLGAMQYHYGCPKPDRELTVLRVGSETGCADNISVPNCNGPLPGPGPYWVKFLALNGSE
PTATTEWSGPITLKTAREPQSIPGMGGARSGAMIAITAILSVLLAILLAALLATLCS

>UPK3c.turtle profiling Ensemble scaffold:PelSin_1.0:JH207163.1:
SIDYTPVITARELEGKITSSTFVLEQPRCVFNDSVSNTDEIWLVVALSNGTSISTFTNPTSLQSLPAF
QKFPGSPHYMTMGTSSLNYPCEKSSGQITVLRVGNETGCVSDTTRPDCNGPLPGLGPYRVKFLAMSPV
TGPTAETRWSDPILLKAGKDPATIDTWPGKRSAGMIVITTILSILLAILLACFIAALTYRC

>UPK2a.human. NP_006751.1

DFNISSLSGLLSPALTESLLVALPPCHLTGGNATLMVRRANDSKVVTSSFVVPPCRGRRELVSVVDSG
AGFTVTRLSAYQVTNLVPGTKFYISYLVKKGTATESSREIPMSTLPRRNMESIGLGMARTGGMVVITV
LLSVAMFLLVLGFIIALALGSRK

>UPK2a.mouse. P38575.1
DFNISSLSGLLSPALTESLLIALPPCHLTGGNATLMVRRANDSKVVKSDFVVPPCRGRRELVSVVDSG
SGYTVTRLSAYQVTNLTPGTKYYISYRVQKGTSTESSPETPMSTLPRKNMESIGLGMARTGGMVVITV
LLSVAMFLLVVGLIVALHWDARK

>UPK2a.dog XP_853307.1
DFNISSVSGLLSPALTESLLVALPPCHLTGGNATLMVRRANDSKVVKSSFVVPSCRGRRELVSVVDSG
AGFTVTRLSAYQVTNLVPGTKYYISYLVRKGSSTESSREIPMSTLPRRQAESIGLGMARTGGMVVITV
LLSVAMFLLVLGLIIALALGARK

>UPK2a.cow NP_776639.1
ADFNISSLSGLLSPVMTESLLVALPPCHLTGGNATLTVRRANDSKVVRSSFVVPPCRGRRELVSVVDS
GSGFTVTRLSAYQVTNLAPGTKYYISYLVTKGASTESSREIPMSTFPRRKAESIGLAMARTGGMVVIT
VLLSVAMFLLVLGLIIALALGARK

>UPK2a.elephant XP_003418225.1
DFHISSLSGVLSPALTESLLVALAPCRLTGGNATLMVRRANDSKVVKSSFMVPPCRGHRELVSVVDSG
AGFTVTRLSAYQVTNLVPGTKYYISYLVKKGTSTESSKESAMSTLPRRKMESTGLGMARTGGMVVITV
LLSVAMFLLVVGFITALALGAQK

>UPK2a.opossum XP_001380648.2
EFNISSLSGLLSPALAESLLVALPPCHLTGGKASLTVRRVNESA**G**MTHNFTVPPCRARRDLVSVVYNS
GSFSITRLSAYQVTNLIPGTKY**F**VYYSVEKGTAVESSNKVQMATLP**R**RKVETLGLGMARTGGMIVITV
LLSVAMFLLVVGLIVALALGVHK

>UPK2a.Platypus profiling AAPN01121652.1 exon2 AAPN01340066.1 exon3
| AAPN01066803.1 exon4; AAPN01269979.1, exon5.
FNISSLSGLLTPTLAESLLVALPPCHLTGGNATLKVQGVNGSS**V**LQQRFVVPPCRGRRELVSVVDSSA
GFATTRLDAYQITGLRPATTY**V**XXXXXXXXXXXXXXXXXXXXXXXXQKAELLVVGMARTGGMVVITVL
LSVAMFLLLLGFIVALVLGSHD

>UPK2b.snake profiling gb|AEQU010390653.1| contig26931230;
gb|AEQU010390653.1| Python molurus contig26931230;
gb|AEQU010042415.1| Python molurus contig26234771,
MDFFRNNSMLLTTFTGTTFIVNIPKCVSSSKFSPATVRIAIAQLPDASSLP**G**ITDTDQIENLRRTPQA
LVYFADEFKDISCRVARDLLVLDLDDSQYELITVVGYQVGGEFCRQTKGPFCNQALKPSTFY**R**VNFFF
LDDKSVIRAHTDWSTAIQTRN**V**TNYESADVMFEGRAGGMIVITILVSVGGAVLLVALIVAVALSSKK

>UPK2b.turtle profiling |AGCU01117019.1| Pelodiscus sinensis
scaffold678_372,**T**LDFFPNASLILAARMSTSFIVNIPKCISSSQFTPTTIRPAVAIVGDKVAMP
AVTDTNQIQSLHSSKFKSVSCRIARDLVSMDIDDGNYKLTTVVGYQVGVEVCEKTKGPFCNQALQPSS
VY**R**VNFFILDEKAVIRAHTGWSDVIQTNN**V**TTFMAYDGSFIGRAGGMIVITVLLSVAMFVLVVGLIVA
AALGGKKS

>UPK2b.lyzard XP_003221456.1; FG735771.1   gb|AAWZ02009508.1|
cont2.9507,
ELDFFPNNSMLLTSFMSTYFIVNVPKCVSPKDFSPVKIRLAVAQVPDTSTLP**G**VIDTDDIANLRRTPQ
ATIYFAGEFDSVSCRVTRDLLVMDMDDSQFELITVLGYQVGAEFCRQTKGPYCNQALKPSTYY**R**VNFF
FLDNKSVIRAHTDWSTALQTRN**V**SDHESADVMFGGRAGGMIVITILVSVGGAVLAIALIVAVALSNKK

>UPK2b.aligator profiling gi|397270035|gb|AKHW01039964.1| scaffold-
4401_1,
TLNFFPNMSNILAARLSSSFIVNIPKCISSSHYTPTTIRPAIAVLGETVGLP**A**VTDTNQIRSLRNFTD
APIYYAGEFASVSCRMARALVKMDIDDENYKLTTIVGYQVGAEVCENTKGPFCNRVLKPSSFY**R**VNFF
VLDENAVVRAHTDWSDPIQTNN**V**TSFSAYDGSFEGRAGGMIVITVLLSVGMFLVVVGLIVAAALGGRK
S

>UPK2b.salamander JK979875, JK978999, JK977433, CO783569
PAFSFFGNQDNIIGNRMGYSFITNIPSCISGAGYTPASILLAVSTSAAVPGVSNTDAIKSLLNDSRAQ
VVYYAGQFNTVPCRVSRDVVQVSSKAADNSFTLTTVLGYQVGSEVCTSVKGLYCNQVLEPGTPYRVNFF
ILDASNVIRAYTDWSDIVTTLNVTNNAQLDSGLSRRSGGMVVITVLLSIALFMLVPALIATLVVGREK
SPLP

>UPK2b.Xlaevis DC123476, DY574479, BJ622989.
SNFQFFTNTDDVLGAVLSQSFMVNVPSCINAVGYVPSTLKVAVANRNPTCMVDTDSIKSLKTDPTAPV
YYTGQMKVPQCRLRRDLEPVKMNSMRDLGYQVGTENCTEVSGPFCNQFLQPGTSYWVNFIILDETDTP
RAYTGWSEPRTTRQVRRLDAVDLGLSGHSGGMVIITVLLSVSVFLLLLGFMAVVVVSRAPNLFTSESK
DCLDMQTQP

>UPK2b.Shark JK941564, JK956747; AAVX01097325.1| scaf_1099306956075,
DYDIALFNGEVSRLATLVWLSPAYCLFEKWVEQRGGELTVRSSASVQVELLRE**D**NATFVLPQRYSVPF
CNDFSPKPAAVSTPLAYQMGPNVACIDGSCIEHVLPGQRFRVRYIIYSVSEEALVTTKWSAPIATRDD
PPSYFSIDADTTPRSGAMVVITTVLVVALFLLLLGFVSMLAARSKFAR

>UPK2A.salamander C097794
QTAQNFSTSLADLPINPLQTLAIVAFPPCWVGVSQSNANLILVSKNSSGSSITNNQTVPVPPCRLRRD
AVFSSDSSSGGTVITNIGFRVTNLTANTTYTASYQSNGVTIGLPTNFTTVQPTNYTAMPEVFARSGGM
VVITVLLSIAMAILVIALILTFVMGRKK

>UPK2a.Xlaevis BP683995
QNTSLADGVLTPLSTSVIIAFPGCKDSGKTVNLIVANGTTTVQNISLQVPQCRLKRDVVVINNSQSGN
VQTVNVGYQIQNLQPGAIYTTYYAVDGSNIPSITFSTRSVSQTVPDIMARSGGMVVITVLLSIAMFVL
LVGLIAVLVIGRK

>UPK2a.Xtropicalis genomic profiling;gb|AAMC01017077.1
scaffold_39_Cont17077,
QNVSQATGVLTPLATSAIFAFPDCTYSGQTVSLVITNSTSTATIIQ**N**ATFQVPQCRLKRDIVVINNGQ
SGNVQTVNVGYQIQNLQPGTNY**M**ATYSSGGISGPSFQFSTRT**V**YPAVANIMARSGGMVVITVLLSIAM
FVLLAGLIAVLILGRK

>UPK2a.coelacanth profiling gi|346202989|gb|AFYH01011847.1|
contig011847
NFNTSLSDNAALISNVYSSFVILSLPPCTYAGKNASVTYSKNSTSE**D**SKTESFVVPPCRFRREVVEVA
RQMEGFTVTDLLGFRVGNLKAGTQY**D**FRYTIDNTTNVLKSNIIQITTSL**V**TSNVLIDEGFKLHSGGMI
VITILLSFAMLFLIIGVIVVLVLGNKS

>UPK2a.skate CV067582; AESE010437608.1
WGS_1_CONTIG_439473;AESE012541543.1| WGS_1_CONTIG_2704617;
AESE011527864.1|WGS_1_CONTIG_1648366,
GFTISLANDNMGEVVASRRSMSAIITMDPNSCNLAGETVIVTVNNTSNGQ**V**IAQPNFVRPVCRNRRDL
ISLVSNADGTPQTLNLGYMLEMLQPSTTY**N**VYLRAGTIRSNMLGVTTIS**P**VDYRTIDLGFGRSGAMVV
ITVILSIAMLALIIAFIVVLVLSK

>UPK2a.zebrafish EH444580, EH464141; gi|312125210:188150-200000
scaffold, Zv9_scaffold619
DIPISLLNPNTDGVLASTFPNSFLLQMPDCSIYGNQSVLLLYTEAPTNLN**N**TVNFTVQPCPVSQSWYL
LGNLKNGTTY**S**MSYKIGNDTSSVLTNTTTNNDYQQIDTGLRARSGAMVVITVILSLAMVFLLVGIILV
FFFFSG

>UPK2a.salmo EG847319 EST_ssal_eve_49206; AGKD01043111.1|
Contig_043115
EFQVSLLKESDGVVTGRFADSLLLSLPPCALATQSVTLEYNNTDTNES**K**TLVNIFKVLPCRFRRDIIS
TIENNAQFTTSRNLGYQVTNLTTGSTY**R**FQYVVGAEKSNILEVSTRQ**V**KDHNQIDSGLPACSGAMMVI
TVILSVSMFILLVALIFTVAHSLGGD

>UPK2a.lamprey

DYQVKLLNTSVVRAAQTPQSVAFAVPSCEIAMIAGTMLNIFVTPNTTG**V**EVIVGQVAVPGCRVARAVS
GQVVSGNDGTGPGIPVPSGVAYRVTGLTPSTSY**S**VVLRHSTLGLQSVPGILSTANARTPRSAVDAEAF
SRSGGMVVITVILSVLIFLLLAILVAALLLGNKN
