# Supplementary material for: Generation of divergent uroplakin tetraspanins and their partners during vertebrate evolution: identification of novel uroplakins
Source: BMC Evol Biol. 2014 Jan 23;14:13. doi: 10.1186/1471-2148-14-13 (PMC3922775; doi:10.1186/1471-2148-14-13)
Supplement: Additional file 2: Figure S2 — List of all uroplakin DNA sequences and their accession numbers used in this study. (A) Tetraspanin uroplakins (UPK1a and UPK1b) DNA sequences. (B) UPK2/3 uroplakins (UPK2a, UPK2b, UPK3a, UPK3b, Upk3c and UPK3d) DNA sequences exons 2–5. http://www.biomedcentral.com/imedia/1744512341035356/supp2.pdf. [file 1471-2148-14-13-S2.pdf]

A.Teraspanin uroplakins (UPK1a and UPK1b)

```
>UPK1a.human  NM006760
ATGGCTTCTGCAGCAGCAGCAACGACAGAGAAGGGGTCTCCAGTTGTGGTGGGTCTGCTGGTCATGGGCA
ACATCATTATTCTGCTGTCAGGCCTGGCCCTGTTTGCTGAAACGGTATGGGTGACCGCTGACCAGTACCG
CATATACCCGCTGATGGGCGTCTCGGGCAAGGATGACGTCTTCGCCGGCGCCTGGATCGCCATCTTCTGC
GGCTTCTCCTTCTTCGTGGTGGCCAGCTTTGGTGTGGGCGCAGCACTCTGCCGCCGCCGCTCCATGATCC
TCACGTACCTGATACTCATGCTCATCATCTACATCTTTGAGTGCGCCTCCTGCATCACGTCCTACACCCA
CCGAGACTATATGGTGTCCAACCCGTCCCTGATCACCAAGCAGATGTTGACATTCTATAGTGCAGACTCG
AACCAGGGCCGGGAACTGACCCGCCTCTGGGATCGCATCATGATTGAGCAAGAGTGCTGTGGCACGTCAG
GCCCCATGGACTGGGTGAACTTCACGTCTGCCTTCCGGGCCACCACCCCAGAGGTGGTGTTCCCCTGGCC
CCCGCTATGCTGTCGACGGACCGGCAACTTCATCCCAGTCAATGAAGAAGGCTGCCGCCTGGGCCACCTG
GACTACCTGTTCACCAAGGGCTGCTTTGAACATATTGGCCACGCCATCGACAGCTACACGTGGGGCATCT
CGTGGTTTGGGTTTGCCATCCTGATGTGGACGCTCCCCGTGATGCTGATAGC
CATGTATTTCTACACCACGTTGTGA

>UPK1a.mouse NM_026815.2
ATGGCTTCTGCAGCGACAGAGGGAGAGAAGGGGTCTCCTGTGGTGGTGGGGCTGCTGGTCGTGGGCAACA
TCATTATTTTGCTGTCAGGCCTGGCCCTGTTTGCGGAGACAGTGTGGGTAACAGCTGACCAGTACCGCGT
GTACCCACTGATGGGCGTCTCAGGCAAGGATGATGTCTTCGCTGGCGCCTGGATTGCCATCTTCTGCGGC
TTCTCCTTCTTCGTCGTGGCCAGCTTTGGTGTAGGAGCTGCGCTCTGTCGTCGTCGGTACATGATCCTCA
CGTACCTGCTGCTGATGCTCATCGTCTACATCTTCGAGTGTGCCTCCTGCATCACATCCTACACCCACCG
CGACTATATGGTGTCCAACCCATCCCTGATAACCAAGCAAATGTTGACCTACTACAGTGCAGACACTGAC
CAGGGCCAGGAGCTAACCCGACTCTGGGACCGGATCATGATTGAGCAAGAGTGTTGTGGCACATCTGGCC
CCATGGACTGGGTGAATTACACATCAGCCTTCCGGGCAGCCACCCCGGAAGTGGTGTTCCCGTGGCCCCC
ACTGTGCTGTCGTCGGACAGGCAACTTCATCCCCATCAATGAAGATGGCTGCCGAGTAGGCCACATGGAC
TACCTGTTTACCAAGGGTTGCTTCGAGCACATCGGCCATGCCATTGATAGCTACACGTGGGGCATATCGT
GGTTTGGCTTTGCCATCTTGATGTGGACCCTCCCTGTGATGTTGATAGCCATGTATTTCTACACCACTCT
CTGA

>UPK1a.Dog  XM_541693.1
ATGGCTTCAGCGGCGACAGAGGCCGAGAAGGGGTCTCCGGTCGTGGTGGGACTGCTGGTGGTGGGCAACA
TCATAATTCTGCTGTCAGGCCTGGCCCTGTTCGCCGAGACCGTGTGGGTGACCGCTGACCAGTACCGCGT
GTACCCACTGATGGGCGTCTCGGGCAAGGATGACGTCTTCGCTGGCGCCTGGATCGCCATCTTCTGCGGC
TTCTCCTTCTTCGTGGTGGCCAGCCTTGGGGTGGGCGCAGCGCTGTGCCGCCGCCGGTCCATGATCGTCA
CGTACCTGGTGCTCATGCTCATCGTCTACATCTTCGAGTGCGCCTCCTGCATCACGTCCTACACTCATCG
CGACTATATGGTGTCCAACCCATCCCTGATTACCAAGCAGATGCTGACCTTCTACAGTGCAGACACGGAC
CAGGGCCAGGAACTGACTCGCCTCTGGGACCGCATCATGATTGAGCAAGAGTGCTGTGGCACGTCTGGTC
CCATGGACTGGGTGAACTTCACGTCAGCCTTCCGGACAGCCACCCCGGAGGTGGTGTTCCCCTGGCCTCC
GCTGTGTTGTCGGCGGAATGGCAACTTCATCCCCCTCAATGAAGAGGGCTGCCGCCTGGGTCACACGGAC
TACCTGTTCACCGAGGGCTGCTTCGAGCACATCGGCCACGCCATCGACAGCTACACATGGGGTATCTCGT
GGTTTGGGTTTGCCATCCTCATGTGGACGCTCCCCGTGATGCTGATAGCTATGTACTTCTACACCACCTT
G

>UPK1a.cow NM_176611.2
ATGGCTTCTGCAGCAGCAGCAACGACAGAGAAGGGGTCTCCAGTTGTGGTGGGTCTGCTGGTCATGGGCA
ACATCATTATTCTGCTGTCAGGCCTGGCCCTGTTTGCTGAAACGGTATGGGTGACCGCTGACCAGTACCG
CATATACCCGCTGATGGGCGTCTCGGGCAAGGATGACGTCTTCGCCGGCGCCTGGATCGCCATCTTCTGC
GGCTTCTCCTTCTTCGTGGTGGCCAGCTTTGGTGTGGGCGCAGCACTCTGCCGCCGCCGCTCCATGATCC
TCACGTACCTGATACTCATGCTCATCATCTACATCTTTGAGTGCGCCTCCTGCATCACGTCCTACACCCA
CCGAGACTATATGGTGTCCAACCCGTCCCTGATCACCAAGCAGATGTTGACATTCTATAGTGCAGACTCG
AACCAGGGCCGGGAACTGACCCGCCTCTGGGATCGCATCATGATTGAGCAAGAGTGCTGTGGCACGTCAG
GCCCCATGGACTGGGTGAACTTCACGTCTGCCTTCCGGGCCACCACCCCAGAGGTGGTGTTCCCCTGGCC
CCCGCTATGCTGTCGACGGACCGGCAACTTCATCCCAGTCAATGAAGAAGGCTGCCGCCTGGGCCACCTG
```

GACTACCTGTTCACCAAGGGCTGCTTTGAACATATTGGCCACGCCATCGACAGCTACACGTGGGGCATCT
CGTGGTTTGGGTTTGCCATCCTGATGTGGACGCTCCCCGTGATGCTGATAGCCATGTATTTCTACACCAC
GTTGTGA

>UPK1a.elephant XM_003420755.1
ATGGCTTCTGCGGCGGCAGAGGTGAAGAAAGGATCTCCAGTTGTGGTGGGGCTGCTGGTCGTGGGCAACA
TCATTATTCTGCTGTCAGGCCTGGCCCTGTTCGCCGAGACAGTGTGGGTGACAGCGGACCAGTATCATGT
GTACCCACTCATGGGCGTCTCGGGTAAGGACGACGTCTTCGCGGGCGCCTGGATTGCCATCTTCTGCGGC
TTCTCCTTCTTCGTGGTGGCCAGCTTTGGTGTGGGCGCCGCACTGTGCCGCCGCCGGTCCATGATCCTCA
CGTACCTGGTGCTCATGCTGACCGTCTACGTCTTTGAGTGCGCTTCCTGCATCACGTCCTACACCCATCG
AGACTTCATGGTGTCCACCCCCTCCCTGATTACCAAGCAGATGCTGACCTTCTACGGTGCAGACACGGAC
CAGGGCCAGGAGCTGACCCGCCTCTGGGACCGCATCATGATTGAGCAAGAGTGCTGCGGTAACTCTGGGC
CCATGGACTGGGTGAACTTCACATCCACCTTCCGGAAGGCCACTCCGGAGGTGGTGTTCCCCTGGCCCCC
ACTGTGCTGCCGCCGGAATGGCAACTTCATCCCCCTTAGTGAGGAAGGCTGCCGCGTGGGCCACGTGGAC
TACCTGTTCACCAAGGGCTGCTTTGAGCACATTGGCCACGCCATCAACAGCTACACGTGGGGCATCTCAT
GGTTCGGGTTTGCCATCCTGATGTGGACGCTCCCTGTAATGCTGATAGCCATGTATTTCTACACCACATT
GTGA

>UPK1a.opossum XM_001370061.1
ATGGCAGGAGAAAAAGGATCCCCAACTGTGGTGGGCTTACTGGTCCTTGGAAACATCATCATCCTGATGT
CAGGCTTGGCCCTGTTTGCGGAGACTGTTTGGGTGACAGCAGATCAGTACCGGGTCTACCCTCTGCTCGG
AGTTTCTGGCAAGGATGACGTCTTTGCAGGAGCCTGGATTGCCATTTTCACCGGCTTCTCCTTTTTTGTG
GTTGCCAGCTTTGGTATCGGAGCAGTACTGTGCCACAGCCGAGGAATGCTTCTCACGTACCTGCTATTAA
TGCTGATTGTCTATGTGTTTGAGTGTGCCTCCAGTATCACATCCTATACCCACAGAGACTACGTGGTGTC
AAACCCAGCACTTATTACCAAGCAGATGCTGACATACTACAATGCCCCAACTAGCCAGGGCCAGGAACTA
ACTCGTTTCTGGGACCGAGTCATGATTGAGCAACAATGCTGTGGTACCTCAGGGCCCCTAGACTGGGTGA
ACTTCACATCAGCCTTCCGAGAGGCCACCCCTGAGGTTGTGTTTCCTTGGCCCCCACTGTGTTGCCGTAG
AGATGCCAACTTCAATCCTCTCAATGAGGATGGATGTCGGCTTGGCCACCAAGACTACCTCTTCACCAAG
GGCTGCTTTGAGCATATTGGTCATGCCATTGATAGCTACACATGGGGGATCTCTTGGTTTGGATTCGCCA
TTCTTATGTGGACGCTCCCTGTGATGCTGTTGGCCATGTACTATTATACGATACTCTGA

>UPK1a.platypus exons 2-6 but exon 5 coo intron Boundary CT instead
of GT; missing exons 1 and 7; profiling AAPN01309122.1 plus
AAPN01141210.1 Cont44002.2,
CTGTCCGGGCTGGCGCTGTTTGCCGAGTCCGTGTGGGTGGCGTCTGACCCGTACCGCGTGTATCCGACGC
TCGGCGTGTCCGGGAAGGACGACGTCTTCGCCGGGGCCTGGATCTCCATCTTCACTGGTTTCGCCTTCTT
CCTGGTGGGCAGCCTCGGCCTCGTGGCACTGCTCCGGCGGAGTCGGCCCATGGTCTTGACGTATCTGGTA
CTGATGCTGATCGTGTACGTGTTTGAAAGCGCCTCATCTATCACCGCACACACCCATCGGGACTACATGG
TCTCCGACCCGTCGCTCATCACCAAGGAGATGTTGACGCACTATGGCGCCCCCAGCCGCCAGGGCCGGGA
ACTGACCCGCCTGTGGGACAGCATCATGGTGGAGAAAGAGTGCTGTGGGACTTTGGGGCCTCTGGACTGG
GTGCACTATACATCGACATTCCGCACCTCGATCCCTGAGGCGGTGTTCCCCTGGCCGCCTCCCTGCTGCC
GAAGGGACCCCAACTTCATCCCGCTCAGCGAGGAGGGCTGCCGCGTCGGCCATCGGGATTATATCTTCAC
CAAGGGTTGCTTTGAGCACATTGGCCACGCCATCGACAGCTACGCTTGGGGGGTTTCCTGGTTCGGATTT
GCGATCCTCATGTGGACC

>UPK1a.aligator profiling AKHW01101299.1| AKHW01101300.1 scaffold-
12507_11
ATGGCAGAGAAGGGGAACCCCCTGGTCGTGGGGCTGCTGATCTTGGGCAACCTCATCATCCTGCTGGCAG
GACTGGCACTGTACGCAGAGACCATCTGGGTCACGGCCGACCAGTACAAGGTCTACCCCATCCTGGGGGT
GTCGGGCAAGGATGATGTCTATGCGGGCGCCTGGATCGCCATCTTCTGCGGCTTCGCCTTCTTCTGCCTG
GGGGTGCTGGGCATCATCGCCCTGGCACGGGGCAGCCGCCCACTGCTTATGGTGTACCTGGCACTGATGG
TCATCGTCTACATCTTTGAGTCGGCCTCATGCATCACGTCCTACACACACCGCGACTTTGTGGTATCAAA
CCCACGGCTTGTGACCAAGCAGATGCTGTCCTTCTACGCGGCGCCATCACGCCAGGGCCGCGAGCTCACA
CGCATGTGGGACCGCATCATGATGGAGCAACAGTGCTGTGGCACGTCAGGGCCCCTGGACTGGGTGAACA
ACACATCGGCCTTCAGGGCCCGGTTCCCTGAGGTGGCCGCGCCGTGGCCCTTCCTCTGCTGTCGCCGTGA
CCCCAACTTCGTCATCCTCAACCAAGAGGGCTGCCGCGTCGGGCACGTGGACTACGTCAACACCAAGGGC
TGCTTTGAACACATTGAGAACGCGGTGAACAGCTACACATGGGGCATCTCCTGGTTCGGCTTCGCCATCC
TCATGCTGGCATGCCCCGTGATGCTGTTGGGCATGTACCACTACACCACGCTGTGA

>UPK1a.lizard FG732695.1; FG756112.; FG756160.1

ATGGCGGCAAAAGGCTCGACTTTGGTTGTGACGTTACTGGTGATGGGCAATGTTATTATCATGCTCGCAG
GACTGGCCCTTTATGCCGAGTCTATCTGGGTAACAGCCGATCCGTACAAAGTGTACCCCATCATGGGAGT
GTCAGGCAAGGATGATGTCTATGCAGGTGCCTGGATTTCCATTTTCACTGGGTTTTCGTTCTTCTGCGTC
TGCGTCTTTGGCATCATCAGCCTCGTGAATGGCAACCGCCTGATGGTGTTGTTGTATCTGGTGCTAATCC
TGGTTGTCTACCTGTTTGAGTGTGCTTCTTGCATCACCTCCTACACGCATCGTGATTTCGTCGTTTCAAA
TTCCAGGTTGATAACCAAACAGATGTTGTCCTACTACTCTGAGGATTCATACCAAGGACGGGAACTCACC
CGTATGTGGAATCGCTTCATGATGGAGCAACAGTGCTGTGGCACCAATGACCCCATGGATTGGGTGAACT
ACACCTCGGTCTTCCGAAGAAGATATCCTGAAATTGTGGCTCCCTGGCCGTTCTTCTGTTGCAAGCGGGA
TCGGAACTTCATTATCATCAATGAGGAAGGATGCCGACTGGGGCATGTGGATTACATCAACACACAGGGC
TGCTTTGAGCACATTAAACATGCCGTACAGAGTTATGCCTGGGGCGTCTCCTGGTTCGGATTTGCCATTC
TTATGCTCACGGTGCCCTGTCGTTCTTCTGGCTATATATCATTACACTACAATGTGA

>UPK1a.salamander JK981846.1
ATGGCGGACAAGGGATCGCCTGGTCTTGTTGTTCTTCTGATCCTTGGGAATGTCGTCATCACGATGTCCG
GTTTAGCGCTGTTTGCAGAAACCATCTGGGCCACGACCGACCCTTTCAAGGTGTACCCCATCCTGGGAGT
GACGGGCAAGGATGATGTGTTTGCTGGGGGCTGGATCGCCATCTTCTGTGGCTTCTCGTTCTTCATGCTG
GGGATCTACGGCGTTCTGGCAACCTTGCGGGGCAGCAGGACCATGGTCATGACGTACCTTGTCCTGATGC
TGATCGTCTACATCTTTGAGTGCGCCTCGTGCATCACTTCCTTTACACACCGAGACTACATGATCAACTC
CAACGTGGTTCAGAAGCAAATGTTGCAGTATTACACCGACTCCAGCCCGCAGGGTCGGGAGTTGACCCTA
CTTTGGAACCGCATGATGATGGAGAAGCAGTGTTGTGGTGTCAAAGGCCCAGCCGACTGGATCCAATACA
CGTCAACATTCCGTAACCAGTACACGGAGTTTGTAGCCCCATGGCCATTTTTCTGCTGCATCAGGGATGC
CAACTTCCTTCCCATCAACCAGGAGGGCTGCCGTGTGGGTGCCGAAGGCTACATTTACACCCAGGGCTGT
TGGGATCACATCAGCAACGCCATTAACAGTTATACCTGGGGCATCTCTTGGTTCGGCTTTGCCATTCTGA
TGTGGACGCTTCTGGTGATGCTCGCCGAGATGTTCTACTACACTAAACTCTGA

>UPK1a.frog profiling gi|268046463|gb|AAMC01106311.1| |267885090|
gb|AAMC01156946.1|
ATGGCAGAGAAAGGGTCTTCGGGGATGGTGACCTTCATTGTGTTTGGGAATATTGTTATATTGCTCTCTG
GCCTTGCGCTGTTTGCAGAGACGATCTGGGCAACCACCGACCCCTACAAGGTCTATCCTATTCTGGGGGT
GACTGGGAAAGATGACGTTTTTGCCGGCGGCTGGATTGCCATATTCTGTGGATTCTCATTCTTTATACTT
GGAGTCTTTGGCATCCTCGCAGTGCAGAGAGGGAGTCGCACTATGGTTCTGACGTACTTGGTGCTGATGA
TGATCGTCTATATATTTGAATGCGCCTCCTGTATCACTTCCTTCACACACAGAGATTACATGATCAACTC
CAATGTGATTAAGGGTCAGATGTTGACGTACTACTCAGACAGCAGCACCCCCCAGGGAAGGGACGTCACC
GGCGTGTGGCTCAGGATGATGCTGGAGAAAAACTGCTGCGGTGTGGACGGGCCCCTGGATTGGGTGGATT
ATTCCTCCACTTTCCGCAAGACGTACAACGAAACCACCGCCCCTTGGCCCTTGTGGTGCTGCCAGAGAGA
CAGTAACTTCCAGATCATCAATCAGCAGGGATGTGTAGTTGGACTCAAGTCTTATGTTTACCAGCAGGGC
TGCTTTGATCACATATCCAATGCCATCAACAGCTACACTTGGGGCATCTCTTGGTTTGGCTTTGCCATCC
TTATGTGGACGATGATCGTCATGTTGGTAACTATGTACAACTACACCAAGATGAACTGA

>UPK1a.coelacanth Ensemble ENSLACP00000006022; ENSLACT00000006074
ATGGCAGATGGGAAAGGAGGTTCGTCCGTTGGGGCGCTCTTG
ATCTTCGGAAACTTGATCATTCTGATGGCTGGCCTGGCTCTGTTTGCCGAGACGATCTGG
GTGAACACCGATGAGTTTAAGGTCTACACGTTCCTGGGGGTCTCTGGAAAGGATGACGTC
TTCGCTGGCGCCTGGATTGCCATCTTCTGCGGCTTCTGTTTCTTCCTCCTTGGAACCTTT
GGGATCTTCGCTGTCTTGAAGCAGAGCAGAACCATGGTCATGACGTACCTGATCCTGATG
CTAATTGTGTACATTTTCGAGACTGCCTCTTGTATCACATCCTTCACGCACAGGGATTAC
GTGGTCTCCAATCCAAATTTTCTGAAGAAACAGATGTTGCAGCTTTACACAAGCAACACG
TCCCAGGGCATCGAGCTCACAGAAGCCTGGAACCGGGTCATGTTAGAGGAGCAGTGCTGT
GGGGTGGAGGGACCCATGGACTGGATTTCCTTCTCCTCCACATACCAAAACAGCATAAGC
AGTCCAAGCCAGACCTCCTGGCCTCTTTACTGTTGCAAACGTGACAAGAACTTCATCATG
TTGAGTGAGTTGGCGTGCTTGATTGGACATAAAGACTTTGTCTTCTCAAACGGCTGTTGG
GACTTCCTCTCGTTCTCTGTGAACAAGTACACCTGGGGCGTTTCTTGGTTCGGATTTGCA
ATCCTCATGTGGACGTTTGTGGTCATGTGTCTGAAAATGTACTTCTACACCATCATCTGA

>UPK1a.zebrafish BE200967; AI396779; EO017774
ATGGGAGCCGTGACATGTTTGATGGTTACAGTTGTTGGCTTGAATGCAATTGCTGCTGCAGCAGGACTGG
CGTTATCTGCGGTGGCCATTTGGGTCGCAGTGGATGGATATAAACTCTACCCGATTTCTGGTGTATCAGG
GAAAGACGACATCTTTGCTGGAGCCTGGATTGCCATATTTACAGGCTTTGCGTTCTTCCTCACATGCATC
TTCGGCATCTTCGCTGCCCTGAAAAGGAGCCGTGCACTCATGCTGGTGTACCTTATCATAATGTTCATCA
TCTTTTTGTTTGAGTCCGCGTCTGCCATCACATCAGCAACCAACCGGGATTATCTGGTTGGGAACAGCAA
CCTTGTAAAGAAACAGATGCTCCAGTATTATGCAGACAGCAGCACACAAGGACAGCAGATTACAATGACA

TGGAACAACGTGATGACTCAGGTCCAGTGCTGTGGAGCTGACGGCCCGACGGACTGGATACAGTATAACT
CCACCTACAGGCAGTTGTTTGGCGCTGCATCTCTATGGCCCCTCGGCTGCTGCAAGAGACAGAGTAGTAA
CTTTGAGGTGGTAGATCCTATAGGCTGTAAAGCTGGCGTGATGAGCAGCATGTTCACACAGGGTTGCTTC
CAGTACATTGAATCGGTGTTAAGCCGTTATACCTGGGCTGTGAGCTGGTACGGTTTCTCCGTTCTCATGT
TGGTGTTTTTCACATTAGTGATCGCCATGATTTACTACACACAGCTGCCCTAA

>UPK1a.salmo profiling gi|354428581|gbAGKD01030455.1 Contig_030458.
ATGGCTAATGGAAAGGGATTTCTCATCCTGGGGAATATATTTGGAGCTGCAGCAGGTCTTGCCCTTTGCG
CTCTGGCCATCTGGGTTGCAGTAGAGCAATACAAACTCTACCCTATAGCTGGTGTGTCGGGGAAAGATGA
CATCTTTGCCGGGGCCTGGATCGCCATTTTCACAGGCTTTGCTTTCTTCTGCATATGTGTCTTTGGTATC
TTGGCTGCTACGAAGAAGAGCCGTGCCTTAATGCTAACATATCTGATCCTGATGTTGATCATCTACATAT
TTGAGTGTGCCTCGTGTATCACTGCAGTCACCCACAGAGACTATCTGGTTGGGAACAGTAATCTGGTGAA
GAAGCAGATGCTGAAGTATTATGCAGCCGAAGGTGACTCTGGCAGTCGGATCACACTGACATGGAACAAA
GTGATGAATGAGGTGGAGTGTTGTGGAACGGATGGCCCAGTGGACTGGATACAGTACAACTCCACCTTCA
GGGAGAAGTTTGGTACAGACTACCCCTGGCCCATCCACTGCTGCAAGAGGAAGAACAACTACCAGGTGGT
GAATGTGAAGGCCTGCAAGAACGGCCAGAACACCACCATGTTCACTAAGGGCTGCTTCAACCACATTGAG
TCTGTGTTCAGTCTATATACATGGGCTATCAGCTGGTATGGTTTCGCTGTGCTAATGTTTATG

>UPK1A.CatFish. CK412483
ATGGTCGTTGTTGTAATATTGAATGTCGTTGCAGCGGCAGCAGGACTGGCGTTATGTGCATTAGCCATTT
GGGTAGCTGTAGATCCATATAAAGTGTACCCAATATCTGCTGTATCGGGGAAGGATGATATCTTTGCAGC
AGCCTGGATAGCCATCTTCACTGGCTTTGCCTACTTCTGCACTGCCATCTTTGGCATCTACGCTGCTCTG
AAAAGGAAGCGGTCACTCGTGCTGTTGTACCTGATCCTCATGTTCATCATCTTCATATTCGAATGTGCAT
CCTGCATCACAGCGGTCACCAACAGAGACTATCTGATTGGAAACAGTAACCTCGTGAAGAATCAGATGCT
GAAGTACTACGCACAGGACAGTAACCAAGGAAGGCAGATAACCGGTACTTGGAACAAAGTGATGAATGAT
GCTCAATGCTGCGGGACAGACAGCCCAATGGACTGGATAGAGTACAATTCCACCTTCAAGCAGACGTATG
GCAGCACATACACCTGGCCCCTCAACTGCTGCCAACGCCAGAACAGCTTTGATCCAGCTGACCCAGTTGG
CTGCATGTTTGGCCAGACCAGTGCAGTATTCAGCAAGGGTTGTTTCAACTACATCCAGACTGTGTTAAAT
CGTTATACCTGGTCCGTGAGCTGGTATGGCTTTGCTGTGCAAATGTTTGTGTTTTTCCTCCTGCTGATCA
CCATTGTGTACTTCCTCCTCCTGGAATAA

>UPK1A.Carp EC392385.1, AU301724
ATGGGAGCAGGAGCCTTGACATGTTTGATGTTTGTAGTTGTTGCTCTGAACGCAATCGCTGCTGCAGCAG
GACTGGCATTATTCGCAGTGGCTATTTGGGTAGCAGTGGTCGGGTATAAACTCTACCCCATATCTGGTGT
GTCAGGAAAAGATGACATCTTTGCTGCAGCCTGGATTGCCATTTTTACCGGCTTTGCCTTCTTCCTCACA
TGCATCTTTGGCATCTTTGCTGCTCTGAAAAGGAGCCGTGCACTCATGATGGTGTACCTTATCATAATGT
TCATCATCTTTCTGTTTGAATGTGCATCTGCCATCACAGCAGCAACCAACCGGGATTATCTGGTTGGGAA
CAGTAACTTGGTAAAGAAACAAATGCTGCAATATTATGGGCAAGACAGCATACAAGGAAAGCAGATTACA
CAGACATGGAACAGAGTGATGGAACAGGTCGAGTGCTGTGGAGCAGACAGTCCAGAAGACTGGATACTGT
ATAACTCCACCTTCAAACAGATATATCGGACATACAACTGGCCCCTCAGCTGCTGCAAGAGACTGAGTTC
CTTTGAGTTGGAAGATCCGGCAGGGTGTAAGGTTGGCCTGACCAGCCCCGTATTCACAAAGGGTTGGTTC
AACTATATTGAATCTGTGTTAAGCCGTTACACCTGGGCTGTGAGCCGGTAA

>UPK1a.spottedgar profiling gi|363782398| gb|AHAT01019516.1|
contig019516,
ATGTCAGAGGGGAGAGGATCCACGCTCATGATGGCTCTGCTGGTCGCAGGGAACCTGTTCCTGGCG
TTGTGTGGCCTGGCCCTCTACGCCGTGGCGATCTGGGTGGCCACGGACGGGTACAGGCTGTACCCCCTCT
CGGCCGTGTCGGGCAAGGACGACATCTTCGCCGGCTCCTGGATCGCCATCTTCACCGGCTTCGCCTTCTT
CTGCGCCGCCGTCTACGGCGTGTTCGCCGCCCTCCGGGAGAGCCGGGCCATGATGCTGCTGTACCTGGTC
TTAATGCTGGTCATCTACATTTTTGAAGCTGCTTCTGCAATCACAGCTGCCACCCACCGGGACTATCTGG
TTGGCAACAGCAATCTGATAAAGAAACAGATGCTGACGTACTATGCGGATGACAGCGACCCGGGGAGACA
GGTGACAACCACATGGAATCGCGTGAACTGCTGTGGGACCGACGGCCCTCTGGACTGGATCAGCTACAAC
TCCACCTTCAGGTCCAAGTTTCCCACTCAGGAGTATCCCTGGCCGCTCAACTGCTGCAAGAGGAAGGACA
ACTTCGAGGTGCTCAACCTGGACGCCTGCCGCATCGGGGACTGGAACTACATGAACTACAAGGGCTGCTT
TGACCACATTGAGTTCGTCTTCAATCAATATGCCTGGGCCATCTGTTGGTATGGCTTTGCTGTCCTGATG
TTTGTGCTGCCCCTGATGCTTCTGGCGATGGTATACTACCTGAAGCTCTGA

>UPK1a.shark EB688237.1; ES415544.1; CV889144.1|

ATGGGGGAGACAAGTGCCTCGCCAGTGCTCAAGAGCGTACTGATCTTCGGAAAATTAGTCCTCATGCTCG
CTGGCATCGCGCTGTTTGCGGAGACTATCTGGGTGGTGACCGATCAATTCCAGGTGTACCCGGTCTTGGG
GGCTTCGGGGAAGGACGATGTGTTTGCCGGGGCTTGGATCGCCATCTTTGTCGGCTTCGCCTTCTTCTGC
CTCGCCGTCTTTGGCATTCTCGCCGTGCTGAGCGAGAGCAGGACTATGGTCATCACCTACCTGGCGCTGA
TGCTAGTTGTCTACATCTTCGAATGTGCCTCCTGTATCACATCCATCACACACCGAGACTATCTCACCTC
CAATCCCAAATTCATCAAGAAGCAAATGTTGCAGTTCTACGGGGACACCTCTTCCAACGGAGGTCGTGAT
CTCACCACGATGTGGAACCATGTCATGCCAAAGGAGCAATGCTGTGGATCAACGGGGCCCGCTGACTGGA
TCCAGTACACCTCTTTCTTCAGGACCATGTACAACGAGACCTTTGCCCCTTGGCCATTCCAGTGCTGNCA
GAGGAATGCCAACTCTCAGATCAACCAGGCGGCCTGTGCTGTTGGACACAAAGACTTCCTATATCAAAGG
GGTTGCTTTGACTATTTCAGCACGGCCCATCACAG

>UPK1a.lamprey EE740596; UPK1aTRACE name:PMAH-aab87f10.g1; Ensemble
scaffold: Pmarinus_7.0:GL480986:-1269:28785:
ATGGCGAAGGAGTCGAACGCAGGGTGCGTGCAGTGGCTGGTGATCATCGGCAACGTGATCATTTTG
CTGTGTGGCATCACGCTGACCGCCGAGACGATCTGGGTGGTGACCGATGGCTATAAGGTGTACCCCATCC
TCGGCATGGCGCACAACGACGACGTCTTCGCCGGCGCGTGGATCGCCATCTTCACGGGCATGGCGTTCTT
CCTGCTGGGCTTCGTGGGGATCGTCGCCGCGCTGCGCATGACACGGAAGCTCCTGCTCGGGTACATCGTA
TGCATGCTCATCGTCTTCGCGTTCGAGTCGGCGTCCTGCATCACCTCGTTCACGCAACGAGACTACCTAG
TGGGCGACCAAAACTTCCTGCTGAAGCAGATGCTGACGGAGTACCCGAGCGACACCTACCCAGCCTACAC
GGACACGTGGAACATGTTTATGCGAGAGCAAAAGTGTTGCGGCTCCAACGGTCCCACGGACTGGCTCATG
TACACCTCCAAGTTCTCCGAAGCTCACAACAACGACGACGTCAACTTCCCGTGGCCCATGCACTGCTGCG
TGCTGAGCAAGGACGGTCCGCCGCTGAACATCACCTACTGCCGGCTCGGCATGGACGGATACGTGAACAC
CGCGGGCTGCTTCGATTACTTCAGCGCGGCCGTGAATCGCTACACGTGGGGCGTCGCCTGGTTCGGATTC
GCCATCCTCTGCTTCACGTTCTTCGTTCTCAGTGGAGCCATGTACCTGTACACGGTCGCATAA

>UPK1a2.lamprey EG022567, profiling Ensemble ENSPMAT00000009881;
scaffold:Pmarinus_7.0: GL476495:371924:395954:-1
CTGTGCGGGATGGCCCTCATGGCCACGGCCATATGGGTGGTCACCGACCCTTACAAAATCTACCCGTTCC
TGGCCGCCGACAACAACACGGACATCTTCGCCGCAGCCTGGATCGCCATCTTCTGTGGCTTTGCCTTCTT
TCTGCTCGGCATCTTTGGGATGTACGCCGTGTGGAAGATGCAGCGGTCTGCCCTGCTCGCGTATTTCATC
CTCATGTTAATCGTGTTCATCTTCGAAGCAGCATCATGCATCGTGATCTTCACTCACAGGGACTATTTGG
TGGGCACAAAGAACTTGATGCTCAAGCACATGCTTCGTGATTACGGCGAGATTCCTTCATTCACGGACAA
CTGGAAGGACTTGATCAAAAATGCTGCGGGGTGAACGGACCCGAGGACTGGATCTCCTACACGTCGTCCT
TTAGCATATACCACAAGGAGGACGATGCCGACAGCCCATGGCCCACGAAATGCTGCGTGCTGAACGAAAA
CGACGATTACCTGTACGGTCGCATGGGCTGCATCCTGGGCAAGCCAGATGCCATCTTCCAAAAAGGTTGC
TATGGACAATTTTCATCGGCAGTTGATGGCTACACCTTCCCTCTTGCCTGGTTTGGCTTTTCCATCCTCG
TCTTGTTCTTCGTGGAGATTGGTACCCTATACCTGTACACGGTGTCGTAA

>UPK1b.human. NM_006952.3
ATGGCCAAAGACAACTCAACTGTTCGTTGCTTCCAGGGCCTGCTGATTTTTGGAAATGTGATTATTGGTT
GTTGCGGCATTGCCCTGACTGCGGAGTGCATCTTCTTTGTATCTGACCAACACAGCCTCTACCCACTGCT
TGAAGCCACCGACAACGATGACATCTATGGGGCTGCCTGGATCGGCATATTTGTGGGCATCTGCCTCTTC
TGCCTGTCTGTTCTAGGCATTGTAGGCATCATGAAGTCCAGCAGGAAAATTCTTCTGGCGTATTTCATTC
TGATGTTTATAGTATATGCCTTTGAAGTGGCATCTTGTATCACAGCAGCAACACAACAAGACTTTTTCAC
ACCCAACCTCTTCCTGAAGCAGATGCTAGAGAGGTACCAAAACAACAGCCCTCCAAACAATGATGACCAG
TGGAAAAACAATGGAGTCACCAAAACCTGGGACAGGCTCATGCTCCAGGACAATTGCTGTGGCGTAAATG
GTCCATCAGACTGGCAAAAATACACATCTGCCTTCCGGACTGAGAATAATGATGCTGACTATCCCTGGCC
TCGTCAATGCTGTGTTATGAACAATCTTAAAGAACCTCTCAACCTGGAGGCTTGTAAACTAGGCGTGCCT
GGTTTTTATCACAATCAGGGCTGCTATGAACTGATCTCTGGTCCAATGAACCGACACGCCTGGGGGGTTG
CCTGGTTTGGATTTGCCATTCTCTGCTGGACTTTTTGGGTTCTCCTGGGTACCATGTTCTACTGGAGCAG
AATTGAATATTAA

>UPK1b.mouse. NM_178924.4
ATGGCCAAAGACGATTCCACTGTTCGTTGCTTCCAGGGCCTGTTGATTTTTGGACACGTAATTGTTGGTA
TGTGTGGCATCGCCCTGACAGCAGAGTGTATCTTCTTTGTATCTGACCAACACAGTCTTTACCCACTTCT
CGAAGCCACCAACAATGATGATATCTTCGGGGCAGCTTGGATAGGCATGTTCGTGGGCATCTGCCTCTTC
TGCTTGTCCGTTCTAGCCATAGTAGGAATTATGAAGTCCAACAGGAAAATCCTCTTGGCGTACTTCATCA
TGATGTTTATAGTGTACGGTTTTGAAGTGGCATCTTGCATCACAGCAGCAACACAACGCGACTTTTTCAC
AACCAACCTCTTCCTGAAGCAGATGCTGATGAGGTATCAAAACAACAGTCCCCCAACCAATGACGACGAA
TGGAAGAACAACGGGGTCACCAAGACCTGGGATAGGCTCATGCTGCAGGACCACTGCTGTGGTGTAAACG
GTCCGTCAGACTGGCAGAAATACACCTCTGCCTTCCGGGTGGAGAATAACGATGCTGACTACCCCTGGCC

TCGGCAGTGCTGCGTCATGGACAAGCTTAAAGAGCCTCTCAACCTGGACGCTTGCAAACTTGGAGTGCCT
GGTTATTACCACAGTCAGGGCTGCTATGAACTGATCTCTGGACCAATGGACCGGCATGCCTGGGGAGTTG
CCTGGTTTGGATTTGCCATCCTCTGCTGGACTTTTTGGGTTCTCCTGGGCACCATGTTCTACTGGAGCAG
AATTGAATATTAA

>UPK1b.dog XM_845047.2
ATGGCCAAAGATGACTCCACTGTTCGTTGCTTCCAGGGCCTGCTGATTTTTGGAAATGTGATTGTTGGTA
TGTGCGGCATCGCCCTGATGGCAGAGTGCATCTTCTTTGTATCCGACCAGCACAGCCTGTACCCGCTGCT
TGAAGCCACCGACAACGATGACATCTACGGGGCGGCCTGGATCGGCATGTTTGTGGGCATCTGCCTCTTT
TGCCTGTCTGTTCTAGGCATTGTAGGCATCATGAAGTCCAGCAGGAAAATCCTTTTGGCGTATTTCATTC
TGATGTTTATAGTATATGGCTTTGAAGTGGCGTCTTGTATCACAGCAGCCACACAACGAGACTTTTTCAC
ACCCAACCTCTTCCTGAAGCAGATGCTGGAGAGGTACCAAAACAGTAGCCCTCCAAACAATGATGACCAA
TGGAAAAATAATGGAGTCACCAAGACTTGGGACAGACTCATGCTCCAGGACCACTGCTGTGGGGTAAACG
GTCCATCAGACTGGCAGAAATATACGTCTGCCTTCCGGACTGAGAACAACGATGCCGACTACCCATGGCC
TCGTCAGTGCTGTGTGATGAATAATCTTCAAGAACCTCTCAACCTGGAAGCCTGCAAGCTAGGAGTGCCT
GGTTACTATCACAAGCAGGGCTGCTATGAACTGATCTCTGGACCTATGAACCGACACGCCTGGGGGGTCG
CCTGGTTTGGATTTGCCATTCTCTGCTGGACTTTTTGGGTTCTCCTGGGTACCATGTTCTACTGGAGCAG
AATTGAATAT

>UPK1b.cow NM_174482.2
ATGGCCAAAGACGACTCCACTGTTCGTTGCTTCCAGGGCCTGCTGATTTTTGGAAATGTGATTATCGGTA
TGTGCAGCATCGCCCTGATGGCAGAGTGCATCTTCTTTGTATCAGACCAAAACAGCCTCTACCCACTGCT
TGAAGCCACCAACAATGACGACATCTATGCGGCAGCCTGGATTGGCATGTTTGTTGGCATCTGCCTCTTC
TGCCTCTCTGTCCTGGGCATCGTAGGCATCATGAAGTCCAACAGGAAAATTCTTCTGGTGTATTTCATCC
TGATGTTTATTGTATATGCTTTTGAAGTGGCATCTTGTATCACAGCAGCAACACAACGAGACTTTTTCAC
ACCCAACCTCTTCCTGAAGCAGATGCTGGAGAGATACCAAAACAACAGTCCTCCAAACAATGATGACCAA
TGGAAAAACAATGGAGTCACCAAGACCTGGGACAGACTTATGCTCCAGGACAATTGCTGTGGTGTAAATG
GCCCGTCAGACTGGCAGAAATACACCTCTGCCTTCCGGACTGAGAACAGCGATGCTGACTACCCCTGGCC
TCGTCAATGCTGTGTTATGAACAGCCTTAAAGAACCTCTCAACCTGGACGCCTGCAAATTAGGAGTGCCT
GGATACTACCATAGTCAGGGCTGCTATGAGCTGATCTCTGGACCAATGAACCGACATGCCTGGGGAGTTG
CATGGTTTGGATTTGCCATTCTCTGTTGGACTTTCTGGGTTCTCCTGGGTACCATGTTCTACTGGAGCAG
AATTGACTATTAA

>UPK1b.elephant XM_003412982.1
ATGGCCAAAGACAACTCTACCGTTCGTTGCTTCCAGGGCGTGCTGATTTTTGGAAACGTGATCATTGGTA
TGTGCAGCATCGCCTTGATGGCGGAGTGCATCTTCTTTGTATCCGACCAGCACAGCCTCTACCCGCTGCT
TGAAGCCACCAACAATGATGACATCTATGGGGCTGCCTGGATTGGCATGTTTGTGGGCATCTGCCTCTTT
TGCCTGTCCATTCTGGGCATTGTAGGCATCATGAAGTCCAACAGGAAAATTCTGCTGGCGTACTTCATTC
TGATGTTTATAGCGTACGGCTTTGAGGTGGCGTCTTGTATCACAGCGGCAACACAACGAGACTTCTTCAC
AACCAACCTCTTCCTGAAGCAGATGCTGGAGAGGTACCAGAACAACAGCCCTCCAAACAATGATGACAAG
TGGAAAAACAATGGAGTCACCAAAACCTGGGACAGGCTCATGCTTCAGGACAATTGCTGTGGTGTAAATG
GTCCATCGGACTGGCAAAAATATACATCTGCCTTCCGGATTGAGAATAACGATGCTGACTATCCCTGGCC
TCGTCAGTGCTGTGTTACGAACAATCTTAAAGAACCACTCAACGTGGAGGCCTGCAAACTAGGAGTGCCT
GGTTACTATCACAGCCAGGGCTGCTATGAACTGATTTCTGGGCCAATGGACCGACATGCCTGGGGCGTTG
CCTGGTTTGGATTTGCCATTCTCTGCTGGACCTTTTGGGTTCTCCTGGGTGCCATGTTCTACTGGAGCAG
AATTGAATAT

>UPK1b.opossum XM_001363019.2
ATGGCCAAGGGTGACTCCACTGTGCGCTGCTTCCAAGGTCTTCTGATTTTTGGAAATGTGATTATTGGAA
TGTGTGGCATCGCCCTGACAGCAGAATGCATCTTCTTTGTATCGGACCAACACAGCCTGTACCCTTTACT
CGAAGCCACTGATAATGATGACATCTATGGAGCAGCCTGGATTGGCCTCTTTGTGGGCATCTGCCTTTTC
TGCTTGTCTGTTCTTGGCATTGTGGGCATCATGAAGTCAAGCAGAAAAATACTTCTGGCGTATTTTATTC
TGATGTTTATAGTATATGCCTTTGAAGTGGCATCCAGTATCACAGCGGCTGTGCAGCGAGATTTTTTCAC
GACCAATCTCTTCCTGAGGCAGATGCTGGAAAGGTACCAGAACAAAAGTCAGTCCAGTAATGATGACATC
TGGAAAAATATGGCCGTGACCGAAACTTGGGACCGCCTCATGCTCCAGGAAAAGTGCTGTGGTGTGAATG
GTCCATCAGACTGGCAAAAGTACAAGTCAGCCTTCCGGGATGAGAATAATGATGCTGACTACCCCTGGCC
TCATCAGTGCTGCGTTATGGACAATCTCTATAAGCCACTCAATCTCGACGCCTGTAAACTCGGTGTGTCT
GGTTACTATCACAGCAAGGGCTGCTACAAGCTGATCTCAGGACCAATGAACCGACATGCCTGGGGTGTTG
CATGGTTTGGATTTGCCATTCTCTGCTGGACTTTCTGTGTTCTCCTGGGCACCATGTTCTATTGGAGTAG
AATCGAATACTGA

>UPK1b.platypus Exon 3 and 6 missing 24 and 28 aa; profiling
AAPN01272338.1 reverse exons 1,2 | AAPN01106002.1|exon 3
AAPN01297767.1 exon4 AAPN01302484.1 exon 5 AAPN01104274.1 exon
Cont11876.1.
ATGGCGAAGGACGACGGGTCGGTCCGCTGCCTCCAAGGCCTGCTGGTGTTCGGGAACGTGGTCATCGCG
ATGAGCGGGCTGGCTCTGATGGCGGAGTGCATCTTCTTCGTGACGGACCCGTGGCGGCTGTACGGGCTGC
TGGAAGCCACGGACAACGACGACGTCTTCGCCGCCGCCTGGATCGGAATCTTCACCGGCTTCTCCCTGTT
CTGCCTGGCCGCCCTCGGCGTCGTGGCTGTCCTCAGGGCCGGCCGCAAGCTGCTCTTGGCG
NNNNNNNNNNNNNNNNNNNNNNNNNNNNNNNNNNNNNNNNNNNNNNNNNNNNNNNNNNNNNNNNNNNNNN
NNTTCACGCCCAACCTCTTCCTGAGGCAGATGCTGGAAAGGTACCAGAACCAGAGCGGCCCGACCAACGA
CGACCAGTACAAGAACAAGAAGGTCACCGACACGTGGGATCGCCTCATGCTCCAGGAAAAGTGCTGCGGA
GTGAACGGCCCCTCGGACTGGCAGGACTACCACTCGGCCTTCCGCCGCACGCACCTGGACGCCGACTTCC
CCTGGCCGCACCAGTGCTGCGCCCGGGACGGGCAGTACCGCCCGCTCAGCCTCGACGGGTGCAAGCTGGG
GGTCGCCGACTACTATCACGAGGAGNNNNNNNNNNNNNNNNNNNNNNNNNNNNNNNNNNNNNNNNNNNNN
NNNNNNNNNNNNNNNNNNNNNNNNNNNNNNNNNNNNNNTTCTGCGTGCTCTTGGGCGTCATGTTCTACT
GGAGCCGGATTGAGCCG

>UPK1b.Gallus XP_416567.3; DR426684.1; BU224014.1
ATGGCAAAAACTGACAATGGCATACGCATCTGTCAGGGTCTATTAATCCTGGGGAATGTGGTCATTGGGA
TGTGCGGTATTGCCCTGACAGCAGAATGCATCTTCTTCGTGTCGGATCCCCACGGTCTCTACCCATTGCT
GGAAGCCACAGAAAACAATGACATCTATGCTGCGGCCTGGATAGGTATCTTTGTTGGCTTTGCACTCCTT
GCTCTGTCTATCCTCGGCATCGTTGGAGTCATGAAGTCCAGCAAGACTTTGCTACTGGTGTATATTATTC
TGATGCTGATCACTTATGCATTTGAAATGGCTTCTTGCATCACAGCAGCAACTCACCGAGACTTTCTTAC
TCCAAACCTCTTCCTGAAGCAAATGCTGGAGAGGTATATGAAGTCAGATACAGACAATAACAATGACAAA
CAGATGACTGAAGGGGTCACAAAGACATGGGATAATCTCATGCTCCAGAACCAGTGCTGCGGGGTGCATG
GGCCCTTGGACTGGCAGGAGTACACATCTGCTTTTCGGATGACACACAACGACGCTGACTATCCTTGGCC
ACACAACTGCTGTGTCATGAACACCCGAAGTGAACCCATCAATCTTGATGGCTGCAAGCTTGGAGTCCCT
GGCTTCTATAACAGCAATGGTTGTTACGATGCTATTTCTGGGCCACTGAACAGGCAAGCCTGGGGTGTTG
CCTGGTTTGGTTTTGCCATCCTCTGCTGGACTTTCTGCGTCCTCCTCGGAACCATGTTCTACTGGAGTAG
AATTGCATACTGA

>UPK1blizard FG709884, FG750007; gb|AAWZ02001347.1|este exones 3-7;
gb|AAWZ02001348.1| FIRST EXON chromosome 3 cont2.1347,
ATGGTAAAAGGTGATACTGGGGTGAGATGTTTCCAAGGCCTGCTGATCATGGGGGTTTTGGGGTGTTGTG
GCCTTGCACTGATGGCAGAGTGCATATACTTCGTCTCAGATCAACACTCATTGTATCCATTACTGGAAGC
TACTGACAATGATGACATCTTTGGAGCTGCCTGGATTGGGATCTTCACTGGTTTCTGCTTCTTCTGTCTT
TCTGTCCTGGGCATTGTTGGTGTCGTGAAGTCAAACAGAACAATGTTACTGGTGTATATCCTCCTGATGA
TGGTTGTCTTTGCCTTTGAAGTTGCCTCTAGCATCACAGCTGTAGTGCATCGAGACTTCCTCACACCCAA
TCTCTTCTTGAAGCAAATGTTGGAGAAATATCAGAATCCAAATCCAATCAACAATGATGACAGATGGAAG
AGTGATGGTATCACTAGGACATGGAACCGTCTCATGGTGCTGGATGATTGCTGTGGTGTGACTGGACCAA
GAGACTGGCAGAACTATACCTCTGTTTTCAGGACAGTGAGCAACGATGCAGATTTTCCCTGGCCACGGCA
GTGCTGCGTTATAGATGTCCTAGGAAAGCCAACCAACTTGGATGGCTGCAAATTGGGAGTGTCCGGGTAT
TACCATGAAATGGGCTGCTATGATACCATTTCTGGTCCAATGAACAGACATGCTTGGGGTGTTGCCTGGT
ATGGATTTGCCGTTCTTTGCTGGGCTACCTGGGTCCTTCTTTGTACGATGTTTTACTGGAGCCGAATTGA
ATATTAA

>UPK1b.turtle Ensemple. ENSPSIP00000002711; gb| AGCU01013879.1|
scaffold639_322,
ATGGCGAAAGGGGACAACGGAGTGCGCTTTTTCCAAGCCCTACTAATTCTTGGCAATGTGATTATTGGGA
TGTGTGGCATTGCGCTGACGGCCGAGTGCATCTTCTTCGTGTCAGATCAGCAGACCCTGTACCCTCTGCT
GGAAGCCACTAACAATGATGACATCTATGGTGCAGCCTGGATTGGAATCTTTGTCGGCTTTGCTCTCTTT
AACCTCTCTGTCCTGGGCATTGTTGGCGTCATTAAGTCAAACAGGACGATGCTGTTGGCGTACATCATTC
TAATGTTGATCACCTATGGATTTGAAGTGGCCTCTTGCATCACAGCAGCAACTCATCGAGATTTTTTTAC
ACCCAACCTCTTCCTGAGGCAAATGCTGGAGAAGTATCAGAACCCAGAGCCAGCTAGCAACGATGATAAG
TGGATGAGTGAAGGCGTCACCAGGACATGGGACCGCCTCATGCTACAGAATCAGTGCTGTGGGGTGAATG
GCCCATCTGACTGGCAGAACTACATGTCTGTCTTCCGGAAAGGGAACAGTGATTCAGAGTTCCCTTGGCC
ACGCCAGTGCTGCGTCATGGATGTTCAAGGCATTCCAATCAATTTGGATGGCTGCAAATTGGGTGTTGCA
GGCTACTATAATAACAAGGGTTGCTATGACATTATCTCTGGTCCAATGAACAGACATGCTTGGGGTGTTG
CCTGGTTTGGCTTTGCCATTCTCTGCTGGACTTTCTGGGTTCTTATCTGCACCATGTTCTACTGGAGCAG
AATTGAATACTGA

>UPK1b.aligator profiling 397239340|gb|AKHW01070659.1| scaffold-8202_6,
ATGGCAAAAGGCGATAATGGTGTGCGCTGTTTTCAAGGTCTGCTAGTTCTAGGCAACGTGATTATTGGG
TTGTGTGGCATTGCACTGACAGCAGAGTGCATCTTCTTTGTGTCGGATCAGCAGACTCTGTACCCTCTGT
TGGAAGCCACAAACAATGATGACATCTATGGTGCTGCCTGGATTGGCATATTTGTTGGCTTTGCTCTCTT
CAATCTGGCTGTTCTTGGTATCGTTGGAGTCATTAAGTCAAATAGGGCAATGCTACTGGCGTATATCATC
CTGATGTTGATCACATATGCCTTTGAGACGGCCTCTTGCATCACAGCAGCAACTCATCGAGACTTTTTCA
CTCGCAATCTCTTCCTGAAGCAAATGCTGGAGAAGTATCAGAACCCAGAGCCACTCAACAATGATGACAA
GTGGATGAGTGAAGGGGTCACCAGGACATGGGACCGCCTCATGCTTCAGCATGAGTGCTGTGGTGTGAAT
GGCCCGTCTGACTGGCAGAACTACATGTCCAAGTTCAGAAACATTAACAGTGATTCAGAATTCCCTTGGC
CACGCCAGTGTTGTGTCATGAATGTTCAAGGGATACCTGTCAATTTGGATGGCTGCAAACTAGGAGTCTC
TGGCTACTACAATGATAAGGGTTGTTACGAGCTTATCGCTGGACCAATGGACAGACATGCCTGGGGTGTA
GCTTGGTTTGGCTTTGCCATTCTCTGCTGGACTTTCTGGGTTCTTCTCTGTACCATGTTCTACTGGAGCA
GAATTGAATATTGA

>UPK1bXlaevis. CD330041.1
ATGAAAGACGATTCTGGCATTCGGTGTTTCCAGTCCCTCCTTATCTTCGGCAATGTGGTCATTGGGCTCT
GTGGTCTGGCCCTGACAGCCGAGTGCATCTTCTTTGTGTCGGACCAGAGTGGTATCTACCCGCTGCTGGA
GGCCACCGACAACGATGACATATTTGGCGCAGCCTGGATTGGCATCTTTGCTGGATTCTGTCTTTTCGTC
TTGTCTATCCTCGGCATCATTGGCATCATGAAGTCGAACAGGAGACTGCTAATGGTGTATCTCATCCTGA
TGTTCATTGTGTATGCCTTCGAAGTGGCCTCCGCCATCACTGCTGCAACTCAACAAAACTTTTTCATTCC
AGAACTCTTCCTGAAACAGATGCTAGAATTTTACCAAAACCCAAACCCAATCAACAATGACAACCTTTGG
AAAATTAATGGAGTCACTCGCACCTGGAACCGCTTTATGCTTCTGAACGGCTGCTGTGGAGTCAACGGAC
CACAAGACTGGCAGACCTACAACTCCGTGTTCAGGCAGTTTAACAGTGACTCTGCCTACCCTTGGCCTCA
GCAGTGCTGCGTCATGAACAGCCTCGGGCAACCCGTAAACCTGGATGCCTGTAAGCTTGGTGTTGCTGGA
TATGTGAATCTGAATGGTTGCTACGACCTGATGGCTGGCCCTATGACCCGTCATGCCTGGGGAGTTGCCT
GGTTTGGATTTTCCATTCTCTGCTGGACATTCTGGGTTCTCCTTGGGAGCATGTTCTACTGGACCCGTAT
TGAGTATTAA

>UPK1b.salamander JK975385 CN045036
ATGGCGAAAGATGCTGGTGTGCAGTTCTTTCAAGGTCTCCTCATTTTTGGGAATGTGGTTATTGCGATGT
GCGGGATAGCCTTAACGGCAGAGTGCATTTTCTTTGTCTCGGACCAGAGCAGCCTCTACCCACTTCTTGA
AGCTACAGATAATGACGACATCTTTGGTGCTGCCTGGATAGGCATCTTCACCGGCTTCTGCCTCTTCGTC
CTGTCCATTGTGGGCATCGTGGGTGTCATGAAGTCCAACCGGAGGATGTTGTTGGTGTACCTAATCTTGA
TGTTCATCGTGTACTGTTTTGAAGTGGCGTCTTCTATTACTGCAGCAACGCAGCGTGACTTTTTCATTCC
GAACCTCTTCCTGCGGCAGATGCTGGAGCTGTACCAGAATCCGAACCCTGTCAACAATGACCAGGCCTGG
AAAATCAACGGTGTTACTGCAACCTGGAATCGCCTCATGCTGCTTAATAATTGCTGTGGTGTGAATGGCC
CGACGGATTGGCAGGCGTACGCGTCGTCTTTCAGGACGGTCAACAGTGATTCGGATTTCCCTTGGCCGCG
CCAGTGTTGTGTGATGAATGACTTGGGACAGCCGGAAAACATAGATGGATGTAAACTTGGCGTTTCTGGC
CATTACTACAACACGGGGTGCTATGACCTAATTGCTGGTCCCTTGATGCGCCATGCCTGGGGTGTTGCCT
GGTTTGGTTTTGCCATTCTGTGCTGGACGTTCTGGGTGCTACTTGGGAGCATGTTCTACTGGAGCCGGAT
TGAATGCTAG

>UPK1b.coelacanth Ensemble ENSLACP00000016648; ENSLACT00000016762
ATGGCATTGAAGGGAAGTTCACCAGTCCGTTGTTTCCAGGGACTCCTCATCTTCGGAAATGTAGTCATAG
CACTTACTGGCCTGACCCTATTCGCAGCATGCATCTTCTTCCTCTCAGACGATAATCGCTATTACCGGCT
TCTTGATGCCTCTGAGAATGATGACATTTTCGCAGCAGCCTGGATTAGCCTCTTTGTTGGTTTTTCCTTC
TTCCTGCTGAGCATTCTGGGTATCATTGGGGTCATGAAGTCCAACAGAACCATGCTTCTAGTGTATATCA
TTTTGATGTTGGTGGTTTACTGTTTTGAGGTGGCGTCCTGTATCACTGCAATAACACACAGAGACTTCTT
CATTCCCAATATGTTCCTAAAGACCTTGCTAGAAAAATATCAGAAACCTAAAGCCAGAGACTTGGATGAG
CAGGGCAAAATTGACGGGGTCACCACTATCTGGAACCGCATTATGCCACAGCACAAATGCTGTGGAGTTA
ATGGGCCCATGGACTGGCAAGAATACAACTCCAGCTTCAGAATGCAGAATAGTGACGCTGACTTTCCCTG
GCCACGTCAGTGCTGTGTTCGTGCCATAAATGGGGAGCCCCAAAATTTGGACGCTTGCAAACTGGGGATA
GTGGGATCCTACTACGCACAGGGCTGCTTTGAGTTTATCGCTGGTCCTCTGAACAGACATGCCTGGGGAG
TTGCCTGGTATGGATTTGCCATCCTCTGCTGGGTTTTCTGGGTTCTGGCTGTCACCATGTTCTACTTCAG
CAGAATCGATTCAACTGAGTGA

>UPK1b.trout FP322998, CU068699
ATGACACCTGATTTGGACACCAAGGACCGCTGCTTTAGGGCCTTGCTTATCCAAGGCAATTTGGTCATTG
CATGCTGCGGGATTGCCTTGATGGCCATGTGTATCTTCTTCATATCGGATCGAGCCGGATTGTATGTTTT
GGTCTATGCTACAGGAAATGACAGCATCTGGAGGGGAGCCTGGATAGGCCTTTTCACTGGGTTTGCCCTT

TTCTGCACATCCATCTTTGGAATGCACGCCATAGTTGTGTCCAAAAGAAATATCCTACTTGCTTACATCC
TCCTGATGGTGATCATATATGCATTTGAGGTGGCATCAGCTATTACTGCTGCAACACATAAAGACTGGTT
TGTCCCTGAACTCTTCCTGAAGCAAATGCTACAGAACTACAACAAACCTCTACCAGATAATCTGCCCAGC
ACCCAGGACCAGATCTATGTAATCAACGGGATAACAGAAGCATGGAACCGGTTCATGACTGAGTCTAAGT
GCTGTGGAGTGTACGGGCCAGAGGATTGGGTGAAGTACGAGTCCCACTTCAGGCAGCAGAACACAGATGC
TGATAATCCTTGGCCCAGACAATGCTGTCAGCAGGATGCTATGGGAGCCATCAGCCATTTAGAAGCCTGC
AAAATTGGGGTTAGCCCTTTCCTGCATGCACAGGGTTGTTATGATTACATTGCTGGTCCTTTGATTACAC
ACGGCTTTGGAGTCTCATGGGTTGGATTCGCCATATTGTGTTGGACATTCTTTGTGATACTGGGAGTCAT
TTTCCACTACACTCAGTTGGATATCTGA

>UPK1b.salmo NP_001134735.1; EG792520; EG874850; gi|354302482|
gb|AGKD01156046.1| Contig_156071; gi|354418425|gb|AGKD01040611.1
Contig_040615,
ATGACACCTGATTTGGACACCAAGGACCGCTGCTTTAGAGCCTTGATTATCTGGGGCAATTTGGTTATTG
CATGCTGCGGGATTGCCTTGATGGCCATGTGTATCTTCTTCATATCGGATCGGGGCGGATTGTATGTTTT
AGTGTATGCCACAGGAAATGACAGCATCTGGAGGGGAGCCTGGATAGGCCTTTTCACTGGGTTTGCCCTT
TTCTGCACGTCGATCTTGGGAATGCACGCCATAGTTGTGTCCAAAAGAAACATCCTACTAGCTTACATCC
TCCTGATGGTGATCATATATGCATTTGAGGTGGCATCAGCTATTACTGCTGCAACACATAAAGACTGGTT
TGTCCCCGACCTCTTCCTGAAGCAAATGCTACAGAACTACAACAAACCACTACCAGATAATCTGCCCAGC
ACCCAGGACCAGATCTATGTAATCAACAGGGTAACAGAAACATGGAACCGGTTCATGACTGAGTCTAAGT
GCTGTGGAGTGTACGGGCCAGAGGATTGGGTGAAGTATGAGTCCCACTTCAGGCAGCAGAACACAGATGC
TGATTATCCTTGGCCCAGACAATGCTGTCAGCAGGACGCTATGGGAGCCATCAGCCATTTAGATGCCTGC
AAAATCGGGGTTAGCCCTTTCCTGCATGCACAGGGTTGTTATGATTACATTGCGGGTCCTTTGATCAGAC
ACGGCTTTGGAGTCTCATGGTTTGGATTCGCCATATTGTGTTGGACATTCTTTGTGATACTGGGAGTCAT
TTTCCACTACACTCAGTTGGATTTCTGA

>UPK1b.spottedgar profiling gi|363798646| gb|AHAT01003268.1|
contig003268,
ATGGTGGCCAAATCTGACATCGGCGTTCGCTGTTTTCAAGCCTTCCTCGCGTGGGGCAACTTCTGCTGTG
GCCTTGCGCTGATGGCCATGTGCATCTTCTTCATCTTTGATCGGGACCACCTGTACGTCCTGGTGTACGC
TACGGGCAATGACAGCATCTGGAGAGCGGCCTGGATCGGCCTGTTCACGGGCTTCGCTCTCTTCTGCACG
GCTGTGTTTGGGATGTACGCCGTCTTGAAGTCCAAAAGGGGGCTCCTGCTTGTGTATATTCTACTGATGA
TTTTCATCTTTGCTTTTGAAGTGGCATCTGCCATCACAGCAGCGACACACAAAGACTGGTTTGTTCCGAA
TCTCTTCCTTAAACAAATGTTGGAGAACTACAACAAGCCACTGCCCCAAAATCTGCCAAGCACTCAGGAC
GAAATCTACAAGAACAGTGGCATTACCAGCACGTGGAACATGATCATGACTGAGTACCAATGCTGTGGTG
TATATGGACCTCAGGACTGGCTGCAGTACAACTCCCAGTTCAGACAGCAGAACTCGGACTCCGAGTACCC
CTGGCCCAGACAGTGCTGTCTGCAGGATGGAACTGGAGGCATTGTGAACGTGAATGCCTGCAAGATTGGG
GTGGAGCCCTACCTCTTCACTCAGGGCTGCTATGATTACATTGCTGGCCCTTTGATTCGACAAGGATTGG
GTGTCTCCTGGTTTGGTTTTGCCATCTTGTGTTGGACGTTTTTTGTGGTGATAGGAGTAATGTTTTACTA
CACTCAGTTGGACTCCTAA

>UPK1B.skate CV547311;.CO049535; CO050503; AESE010043837 exons 1-5
gi|363355253|gb|.1| LER_WGS_1_CONTIG_4389;
gi|363324303|gb|AESE010074792.1| CONTING EXON 6
LER_WGS_1_CONTIG_74854.
ATGTCTAAAAGTACTGGAGTGAGGGTTGTCCAAGGACTACTCATTTTTGGAAATGTAGTAATCATGTTAA
GTGGCCTTGCATTGACAGCTGAATGCATCTTCCATGTTTCGGATCAGCATAACCTCTGGCCCTTCCTTGC
AGCTGCTGATAATAGTGACATTTTTGCTGCTGCATGGATCGGGCTTTTTGCTGGCTTTTGTCTGTTTTGT
CTGAGCATTGTTGGTATATTTGGAATCATGAAATCGGCCAAGAAAGTTATTTTGACATACCTAATATTGA
TGCTAATTGTCTACATATTTGAAGTTGCCTCTTGTATCACAGCTGCAACACAAAGAGACTTTTTTGTTCC
GAATTTCTTCCTCAAACAAATGTTGCAGTTGTATGGTAATCCAAACCCACTGACTGAAGCTGAAATCAGA
AACACTTTCGGCGTTACAAATGCTTGGAATCGCGTCATGCCTGATAACGACTGTTGTGGTGTAAATGGAC
CTCGGGACTGGGTTCAACATAACTCTGCATTCAGAGTAGTCAATTCTGATTCTCGGTTCCCCTGGCCTCG
ACAGTGTTGTACCACGGACCAATTTGGCCGAATGCTAAATGTAACTGGCTGCAAACTTGGTATTCCTGGC
TATGTTTATGAACAGGGTTGCTTTGATTTCATCGCTGGACCTATGGATAGACATGTTTGGGGTGTAGCAT
GGTTTGGATTTGCCATTCTTTGTTGGACA

>UPK1b.Lamprey FD710561; EB084275 1220567368gi; TRACE
gnl|ti|1470569568 reverse exon4 name:PMAC-bnw68a06.g1 mate:1470753301;
gnl|ti|1423504665 name:PMAC-axy03h10.g1 reverse exon 6 y 7
ATGAGGGACGTCTCGTCTGGAGTGAGATGCGTG

```
CAGATTCTGCTGATCGTTGGGAATGTAGTCCTCCTGGGTTGTGGCCTGGCTCTGTTCGCCGTGTCTGTGT
TCGTGGTGGCCGACCCAGCTCGGCTGTGGCCACTCCTCTCCGCCTTCAACAACACGGACATCTTTGCCGG
CGCCTGGATCAGCATCTTCACCGGGTTCACCTTCTTCCTGCTCGCCATGCTGGGCATCTACGCAGTGATG
AGGCTCGCTCGGGGGCTGCTGCTGGCGTACATTCTGCTCACGCTGTTTGTCTACATCTTTGAAGTGGCCT
CCTGCATCACCGCAATCACCCACAGAGACTTTCTCACCAAGAACGCCTACCTACAGCAGATGCTACAGCT
CTATCAGAACCCCAACTGGGTTGATGAAAACTCCAGATTCAACTACGAAGGGGTCACAGCCGTGTGGGAT
CGCACCATGTTCAACTATCAGTGCTGTGGAGTGAATGGCCCCCAGGACTGGCAGGCCTACGGCTCGGCCT
TCCGAGAGGCCCACCCCGAGGGAGATTTCCCCTGGCCCTTGCAGTGTTGCAGCCTGGACCCGGCCAGCGG
CCTCATCAGGGACCTCGATGCCTGTCGGCTGGGCCTGGCTGGGTACTTGAACACCCCGGGTTGCTATGAC
TACGTGAGTGGGCCTCTCCACCGCGCCGCTTGGGGTGTCGCCTGGTTCGGGTTCGCCATCCTCTGCTGGG
AGTTCTGGGTCCTGCTGGGCACCATGTACCTCTATAACATCTCCTAG
```

B. UPK2/3 uroplakins (UPK2a, UPK2b, UPK3a, UPK3b, UPK3c AND UPK3d). Exons 2-5

```
>UPK3a.human NM_006953.3
CTGTGAACCTGCAGCCCCAACTGGCCAGTGTGACTTTCGCCACCAACAACCCCACACTTAC
CACTGTGGCCTTGGAAAAGCCTCTCTGCATGTTTGACAGCAAAGAGGCCCTCACTGGCACCCACGAGGTC
TACCTGTATGTCCTGGTCGACTCAGCCATTTCCAGGAATGCCTCAGTGCAAGACAGCACCAACACCCCAC
TGGGCTCAACGTTCCTACAAACAGAGGGTGGGAGGACAGGTCCCTACAAAGCTGTGGCCTTTGACCTGAT
CCCCTGCAGTGACCTGCCCAGCCTGGATGCCATTGGGGATGTGTCCAAGGCCTCACAGATCCTGAATGCC
TACCTGGTCAGGGTGGGTGCCAACGGGACCTGCCTGTGGGATCCCAACTTCCAGGGCCTCTGTAACGCAC
CCCTGTCGGCAGCCACGGAGTACAGGTTCAAGTATGTCCTGGTCAATATGTCCACGGGCTTGGTAGAGGA
CCAGACCCTGTGGTCAGACCCCATCCGCACCAACCAGCTCACCCCATACTCGACGATCGACACGTGGCCA
GGCCGGCGGAGCGGAGGCATGATCGTCATCACTTCCATCCTGGGCTCCCTGCCCTTCTTTCTACTTGTGG
GTTTTGCTGGCGCCATTGCCCTCAGCCTCGT

>UPK3a.mouse NM_023478.2
CTGTGAACCTCCAGCCCCAACTGGCCAGTGTGACCTTTGCCACCAACAACCCTACCCTCACCACCGTGGCC
TTGGAGAAGCCTCTGTGCATGTTTGATAGCTCAGAGCCACTCAGCGGCTCTTACGAGGTTTACCTCTATGC
TATGGTCGACTCAGCCATGTCCAGGAATGTGTCTGTACAGGACAGCGCTGGCGTCCCACTGAGCACCACTT
TCCGGCAAACCCAGGGTGGGAGGTCAGGCCCCTATAAAGCTGCGGCCTTTGACCTGACCCCTTGTGGTGAC
TTGCCCAGCCTGGATGCTGTTGGAGATGTGACCCAGGCCTCAGAGATCCTGAACGCATACCTAGTCAGGGT
GGGCAACAACGGGACCTGTTTTTGGGACCCCAACTTCCAGGGCCTCTGCAACCCACCCCTGACAGCGGCCA
CTGAGTACAGATTCAAGTATGTCCTGGTCAACATGTCCACAGGCTTGGTGCAGGACCAGACACTATGGTCA
GATCCCATCTGGACCAACCGGCCCATCCCCTACTCGGCCATCGACACGTGGCCCGGCCGGCGGAGTGGAGG
CATGATTGTCATCACGTCCATTCTGGGCTCCCTGCCCTTCTTCCTGCTCGTGGGTTTCGCTGGAGCCATCA
TCCTCAGCTTTGT

>UPK3a.dog XM_846321.1
GTGTGAACCTCCAGCCCCAACTGGCCAGCGTGACCTTTGCCACCAACAACCCCACCCTCACTACTGTGGCC
TTGGAAAAGCCTCTGTGCATGTTTGACAGTGCGGCGACCCTCAATGGCACCTATGAGATCTACCTCTATGT
CCTGGTCAACCTCGCCAGCTCCAGGAACGCCTCGGTGCAGGACGGTGCCAGGGCCCCGCTGAGCTCCACGG
TCCAGCAGACAGAGGGAGGGAGGACAGGCCCCTATAAGGCCGTGGCCTTTGGCCTGATCCCCTGCAGTGAC
CTGCCCAGCTTGGACGCCGTGGGGGACGTGGCCCGGGCCTCGGAAATCCTGAACGCGTACCTCGTCAGGGT
GGGAGCCAACGGGACCTGCCTGTCAGACCCCAACTTTGGGGGTCTCTGCAACGCGCCCCTGTCAGCAGCCA
CGGAGTACAGGTTCAAGTACGTCCTTGTCAATATGTCCACGGGCTTGGTGCAGGACCAGACCCTGTGGTCC
GACCCCATCCGCACCAACCGGCCCACCCCGTATGCGGCGATCGACACGTGGCCGGGCCGGCGGAGCGGGGG
CATGATCGTCATCACGTCCATCCTGGGCTCCCTGCCCTTCTTCCTGCTGGTTGCCTTTGCCGGCGCCGTTG
TCCTCAGCCTTTT

>UPK3a.cow NM_174709.3|
```

GTGTGAACCTCCAGCCCCAACTGGCTAGTGTGACCTTTGCCACCAACAACCCCACCCTCACCACGGTGGCC
TTGGAAAAGCCCCTCTGCATGTTTGACAGCTCAGCGGCCCTCCACGGCACCTACGAGGTCTACCTCTATGT
GCTGGTCGACTCGGCCAGCTTCAGGAACGCCTCTGTGCAGGACAGCACCAAGACCCCTCTCAGCTCGACGT
TCCAACAGACCCAGGGGGGGAGGACAGGGCCCTACAAGGCCGCGGCCTTTGACCTGACCCCGTGCAGTGAC
TCGCCCAGCTTGGATGCTGTCCGGGATGTGTCCCGGGCCTCGGAGATCCTGAACGCCTACCTGATCAGGGT
GGGCACCAACGGGACCTGCCTGTTGGACCCCAACTTCCAGGGCCTCTGCAACCCACCCCTGTCTGCAGCCA
CGGAATACAGGTTCAAGTACGTCCTGGTCAATATGTCCTCGGGCTTGGTGCAGGACCAGACCCTGTGGTCG
GACCCCATCCGCACCGACCGGCTCACCCTGTACTCGGCGATAGACACGTGGCCCGGCCGGCGGAGCGGGGG
TATGATCGTCATCACGTCCATCCTGGGCTCCCTGCCCTTCTTCCTGCTCATCGGCTTTGCTGGCGCCATCG
TCCTCAGCCTGTG

>UPK3a.elephant XM_003420898.1
CAGCCCCAGCTGGCCAGCGTGACCTTCACCACCAACAACCCCACGCTCACCACTGTGGCATTGGAGAAGCC
GCTCTGCATGTTTGAAAGCAACACTGCCCTTGTTGGCACCTACGAGGTCTACCTCTACGTCCTCGCTGAGT
CGGCCAGCCTCAGGAACGCCTCCATACAGGACCACACCAGTGCCCCGCTGAGCTCGACATTCCAGCAAACA
GAGGGCGGGCGGACAGGCCCCTACAAGGCCGCGGCCTTTGATCTGCAGCCCTGCAGCGACCTGCCCAGCCT
GGATGCCGTGGAGGATGTGTCCCGGGCCTCAGAGATCCTCAGCACATACCTGGTCAGGGTAGGCGCCAATG
GGACCTGCCTGTCCGACCCCAACTTCCAGGGCCTCTGCAACCCACCCCTGTCGGTGGCCACGGAATACAGG
TTCAAGTACGTCCTTGTCAATATGTCCACGGGCTTGGTACAGGACCAGACCCTCTGGTCGGACCCCATTCG
CACCAACCGGCTCACCCCTTACTCAGCGATTGACACGTGGCCTGGGCGGCGGAGCGGAGGCATGATTGTCA
TCACAGCCATCCTGGGCTCCCTGCCCTTCTTCCTGCTCCTGGGATTCGCGGGTGCCGTCCTCCTCAGCTTT
GT

>UPK3a.opossum XM_001378398.2
CAGTAGACCTGGAGCCTCAAATGGCCAGTATCACATTTGCCACAAATAACCCTACCTTGACCACCATC
ACTCTGGAGAAGCCCTTCTGTATGTTCAATGCCTCTAACCTTGGGAATGTCTCCTATGAGGTCAACCTGTA
CGTGATGGAGAATTCAGGAAGTGTTAGATATGCGGTCATCAAGAACAATCGCAGCTTCCCAATCAACAGCA
CATTCCAGGAGACTGCAGGGGGCCAGAGGGCACCATACAAGGCTGCTTCCTTCATCCTGCCCCAGTGTGGT
GACCTGCCCAACCTGGATGAGGCTGGGGATGTGAGCAAAGTAGCTGAGATTCTGAGTGCCTACCTGGTCAG
GGTTGGAGACAATGGCTACTGCTCACTGAATCCTAACTTCAAGGGCACCTGCAACCCACCTCTGACAAGGG
CCACAGAATACAGGTTCAAATATGTCTTGATCAATCCATCAAGTGGTTTTGTAGAGGATCAAACCCTCTGG
TCTCAGCCAATCCGGACCAACCAGATTTCCCCCTACTTGGAGATTGACACATGGCCTGGCAGGAGGAGTGG
AGCCATGATAGTCATCACTTCCATCCTGAGCACCCTAGTATTCTTCCTGCTGGTAGGGTTTGCAGCTGCTG
TCATCTTCAGCTTTGT

>UPK3a.platypus profilinf AAPN01155619.1; AAPN01135149.1Cont1295.23,
CTGCCCAGATCAAGCCTCAGATCGCCGGTTTGGCATTTGCCACGAACAACCCTACCCTCACGACGATCGCC
CTGGAGAAACCCTTCTGCATGTTCGACGGCGCCGGCCCTGCCCAACTGCCCTCCGAGATCTACCTCTATGT
CATGATGGACTCTGCGGACGCCCACCCGGTGCTGGACAACGGGAGCCGTCCTCTCCGGACGACATTCCAGC
AGGCCGCGGGTGGCCGCCGGGGCCCCTACTGGGCCGCGACCTTCAGCGTTCCCCGGTGCGAGGACCTCCCC
CAGCTGTGGGACGCCGGGGACCCTGCCAGGGCCCCCAGATTCTGGACGCCTACCTCTTCCGCGTCGGCGG
CGACACCGCCTGTATGTGGGACCCCGACTTCAGCGGGGCGTGCAACCCGCCCCTGGCCGGGGAGACCGGGT
ACAGGTTCAAATATGTGTTGGTGAACACAACAAGTGGCTCGGTTGTGGATCAGTCCCTCTGGTCCGACCCC
ATCAGGACAAAGCGCACCCTGTCGTGGTCGCGCGTGGACACCTGGCCAGGGAGGAGGAGCGGGAGCATGAT
AGTCATCACGTCCATCCTGAGCTCCCTGATGTTCGTCCTGCTGGTCGGCCTGGCCGCCGCCGTCACCTGCC
GTGTCCT

>UPK3a.chiken BU410839, BU464912 and profiling NW_001471513.1
Gga1_WGA14_2:27108888-27154218
ATCAGAGCATGAAACCTCAGCTTGCAGCCCCCGAGCTTGCAACAAACAATCCCACTCTCACTACAGTTGCC
CTAGAGAAACCTTTCTGTATGTTTGATAGCTCACTACATCCAAACAAATCTTATGCCATCTACTTGTATGT
GATGAAAAGTTCAGCCAACACAATAAGCTCCGTGGTGACTGACAGCAGCAGCAAGCCACTGGACAGCACGT
TCCAGCAAACACATGGGGGACATCTTGGACCTTACAAGGCTGCCTCATTCGATGTACCCAACTGTGTGTCA
CCCCCCAGGCTTGCTGATGCAGGAGACATCAACAAAGTTTCCGATGTCCTGAAACAATACCTCTTCAGAGT
TGGGGATGATGGGACTTGTTTGTATGACCCAAACTTCCTAGATGTCTGCAACCCACCTCTTGCACCAGACA
CAACATACAGGTTTAAATACGTGTTGGTCGATAACACTGAAGGTATCGTGAAAGACCAAACTCTTTGGTCT
GATCCAATCAAAACCAGAAAAGCTAAACTTCCCATGAAAATTGATATCTGGCCTGGTCGAAGGAGTGGAAG
CATGATTGTCATTACATCAATTCTAAGTGTGTCAGTGTTCCTTCTGCTTGCTGGCTTGCTTGCTTCTGTGT
TTTCTGCTCTCGT

>UPK3a.aligator profiling gb|AKHW01039965.1| scaffold-4401_2,

CTCAGAGTCTGAGGCCACAGATTGCAAGCCCCCAGCTGGCTACAAACAATCCCACCTTTACTACAATTGCT
TTAGAAAAACCTTTTTGTGTATTTGATGGCTCACTGTCCCCAGGTAAATCCTATGAAGTATATCTGTATGC
AATGATGGATTCTATGAGCATGATCAGCTCAGCCGTGACTGACAACAGTAGCAAACCTCTGGACAGCACTT
TCCAGGAAGTGAACGGAGGCCAGCTTGGACCTTACAAGGCAGCAGTGCTAAATGTGCCTGACTGTGCATCG
CCTCCCAAACTAGCTGATATTAGAAATGTCAAAAAAGCTTCTGATGTCCTGAAACAGTACCTCTTCAGAGT
GGGAGATGATGTCTCTTGTTTGTATGATCCAAACTTCCTAGGTGTCTGTAACCCACCTCTGGCTTCAGATA
CCACCTACAGGTTTAAATACATCTTGGTAGATGAGACTTTGGGTATCATGAAAGATGAAACTCTCTGGTCT
GATCCAATCAAAACCAATAGCGTTAAAAAGTCTTCAACAATTGATACCTGGCCTGGTCGAAGGAGTGGAGG
GATGATTGTCATAACCTCAATCCTGAGCGTCCTTATCTTTCTCCTCCTTGCAGGACTTTTTGCCTCTGTAT
TCTTTGCTGTCAT

>UPK3a.turtle profiling gi|350386162|gb|AGCU01117018.1|
scaffold678_371
CTCTGAATTTGAGGCCTCAGATTGCAAATCCCAAACTTGCTACAAGCAATCCTACCCTTACCACAATTGCA
CTAGAGAAACCTTTCTGTATGTTTGATGATTCACTGTCTCCAGGGAGCTCCTATGAGGTCTACTTGTATGC
AATGGCTGACTCTGAAAGTACTGTAAGCTCAGCTGTGACTGACAACAGCAGCAAACCTCTCAATACCACCT
TCCAAGACACCAATGGAGGACAACTTGGACCTTACAGGGCTGCATTGTTTAATGTGCCCAACTGTGCATCA
CCCCCTATGCTAGCTGATGTTGTGAATGTGAAAAAAGTTTCAGATGTCTTGAAACAATACCTCTTCAGAGT
GGGAGATGATGTGACTTGTTTATATGACCCAAACTTTCCTGGTGCCTGTAACCCACCCCTTGCTCAGGACA
CAACATACAGGTTTAAATACCTCTTAGTAGATGTGAATGCAGGTGTTGTCAAAGACCAAACTCTCTGGTCT
GACCCAATGAAAACCAGAAGGGTAAAACAGTCCTCAACAATTGATACCTGGCCTGGGCGAAGGAGTGGAGG
AATGATTGTCATCACATCAATCTTAAGCACTCTTATGTTCATTCTAGTTGCTGGCTTTCTTGCCTCCCTAT
ACTTCATTGTCAT

>UPK3a.frogXt BX737880 CX441249 ENSEBLE
scaffold:JGI_4.2:GL173822.1:32034:48169:1
CCGGAGCAGACATGCCGTTGCTGGCCAACTCGGATTTCTTTAGCCTCAATCCCACCCAGACTACG
ATTGCGCTGGAGCAGCCGATCTGCATGTTTAAATCGGCCGTTAACGTCTATCTAATCGGGATCGTGGCAG
GTGCCCCAAACACCCCGCTTTATGACGGCAATAAGAAGGTTAATGCCTCAACCTACAGCGGAACCCAGGG
GGGCAAGACGGGACCCTATATCGTGGCCAAACTTCCCAACCAACAATGTATCAACATACAGGCCCTGAGC
AATATGGCCGACCCCACGCAGGTGCAGTCCATCCTTAGCAAATACGTCGTGAGAGTGGGGGCCGACGTGA
CCTGCTTAACCAATCCCAACTTTGTGGGGTACTGCAACGCCCCCCTGCAGGGGAACACTCAATACAGTTT
CAAGTATCTGTTTACAGACAGTGGGGATATTGTGCAGTCCGAGACCAGCTGGTCCCTGGGCATCACTACC
GTCAATGGCAAAGCTTCTAGTACCATAGACACCTGGCCGGGCAGGAGGAGTGGTGGCATGATTGTCCTGA
CCTCCATTCTCAGCACTCTGATGTTCTTCGTTTTCATTGCCTACGTCATTGGCTTTGCTTACTCCATAGC

>UPK3a.frogXl DC114372, DT404162
CCCGATCGGCTGTTCCTTTGCTTGCAAACTCCGATTTCTTTAGCCTCAATCCCACTCAGACTAC
GATAACGTTGGAACGGCCGTTCTGCATGTTTAAAGATGCCATTGACGTTTATCTCTTTGCCATTGTGAAA
GGTGCCACAAACATCCAAGTTGCTGATGCTGCCAAGAAGGTTATTGCCTCTAACTACACTGGAACCCAGG
GAGGCCTACTGGGACCATACCAAGTTGCCAAACTTGACAATCCAAAATGTGAAAACATACAGGCCTCCAA
CATTATGGCTGACCCCAACAAGTACATTGTGAGAGTGGGGGGCGACGTGAACTGCTTAACGGATCCAAAC
TTTAAGGGGATCTGCAACCCTCCACTTCAAAATAACTTACAATACAGGTTTACATATGTATTTACGATTG
GGGATGTCGTGCAGTACCAGACTGACTGGTCCCCTCCAATCTCTACAGTCAACGTCAAATCTTCCGGCAC
AATAGACACATGGCCTGGCAGAAGGAGTGGTGGGATGATTGTCCTGACTTCCATTCTCAGCACTCTGATG
TTCTTCGTTTTCTTCGCTTACATTGTTGGCTTTGCCTATTCCATACT

>UPK3a.salamander JK978030, JK980634, DT404162.
CCTTCGCCGTCACGCCGCAGGTCGCGGGCCCCGCGCTGGTGCCGAACACCCCCACGCTGAACACGGTGGCG
CTGCAGAAGCCCGTCTGCCTCTTCGACAGCCAGACGGGCGGGGACCCGGCCAACTACCAGGTCGAGCTGTT
CGCCATGGCGGCCAGTGCGCCCAGCAGCACCCCCCTGGCGAGCGGCAACACGTTCCGGAACACGAGCGGGG
GCACCACCGGGCCCTACGTAGCCGGGAAGTTTGGCGTGCCCAATTGCACTCTGGTCGGCTCCCTTACCCCC
TCCCAGGACGACTTTCTGAGGCAGTACATCTTTCGAGTTGGAGATAATCCCACCTGCTTGACCGATCCGAA
CTTCTCCGGCATCTGCAACCCTCCCCTTGCTAATAGCACGGCCTACAGGTTCCTGTATTCCCTGGTGCACA
ACAATGGGAGTCGTGTTGCCAACACACACTGGTCCGATCGAATCTCTACAAAGAACGTCAAGACCCCGGAC
ACGCTGGACACTTGGCCTGGCAAAAGAAGCGGGGGTATGATTGTCATCACGTCCATCCTAAGTACCCTCCT
CTTCTTCCTGCTCTCGGGGTTCGTCGCTGCTGCCGCCGCCAACGTTCT

>UPK3a.coelacanth Ensemble scaffold:LatCha1:JH126738.1:234530:268960:-
1; gbAFYH01050375 gi|346164461|gb|.1| contig050375.

CTCAGTTCCCAAAGCCAGAGATTGCAAATCCTCAGTTTGTAGCAGGCAACCCCACCCTGACGACAATCTCA
TTGGAAAAGCCGTTCTGTGTGTTTGATCGAACCATCAGTGCAAGCAGTGGAGATTTTACAGTTGCAGTGTT
TGCAGTAAAAAGCACTGTAGCTGTTGCTGAAAATATTAATGATTTTACACAAACTTATCAGAGTTCAAGAG
AAGGAACCACGGCCCCTTACAAGGCTGCATCCTTTGCTGTCCCCAATTGTGACTCTCCACTTGTTTTGAGT
AATCCTGTGAACGTGAACGTGATTCGGTCTTTGCTGAACCAGTATCTTATCAGAATTGGGGATGACACAAT
GTGTTCTAATATGCCAGGGATATGTAATGGGCCTCTGACTCCGAACACAGCATACAGATTTAAATTCGTGC
TTCTGAACGGCAATCGTCCCGCAGCCCAAACACCGTGGTCCAGTATGATCTCTACTAGGAAATCCAAACCG
TTTGAGGATATTGACACCTGGCCTGGGAAAAGGACTGGAGGACAAGTTGTTGTTACTACAATCCTTGTTAT
TCTCCTCTTTCTCCTGCTCTGTGGATTTGTTACCACCTTGGTAGCTAGTATTCT

>UPK3a.spottedgar   genomic profiling AHAT01033516.1 contig033516
CAGAGGCTCTGAAGGTGAAGCCCGAGGCTGTGTCTCCCAGGCTGCTGCGGTTTAACCCGACACAGAGCACG
GTGTCCCTCGCGAAGCCGCTTTGCGTTTTCGACAGCGTGAAGCCAACGAATGAAATGATGGTAGATGTATA
CGTTGTCCACAGCCTCTCGGCCACTCTGACCTTTGAGACTGGGAAAACGTACAAGGAGACGAATGGAGGGA
CAGAGACTCCTTATAAGGCTACATCCTTCGGGATCCCAAACTGCACCTCTCCACCAAACCCTGCAGACCTG
TCTGTTCCCCAGAGAATCGATAAGACTTTAGATGAGTACTTGGTCCGTATTGGCAGCAACCCGACATGCGT
GGGGGAACCGGAGGCTGAAGCCTTTTGTAATGCGCCGCTCTCCGATGGCACTTCGTACAGGTTTAAATATT
TGCTTGTAAATGGGACTACAACTATAGCAGAAACCGAATGGTCAGAGTCAATCCTAACAAGAAAGGCTCTG
AGCCCTGATGAAATTGACACCTGGATTGGGAAGAGAAGTGGAGGCGGGATAGTTGTCACTGTCATCCTGAG
TCTCCTGCTGTTTCTGCTGCTTGGGGCTGCCATTTTCATGGGAGTACTGGATGTGAT

>UPK3a.skate EE989195; GH546922; AESE010876980.1WGS_1_CONTIG_884036, AESE010997029.1;WGS_1_CONTIG_1005178;AESE012684067.1 WGS_1_CONTIG_2848843
CTCTGAGTTTCAAGCCTGAAGTCTCCTCCATCGCTTTGACTCTGGGCCTCCGGACATCCACGACCGTGACC
CTAGCCAAGCCTATCTGTGTGTTTTCCAGCGGTGATGTGGTCGAGGTGTTTGGAGTACAGACAACGGCAGA
CTCGATCCCAATTGAAATCGGCAACAGGACCATATTGACTTACCAACAAACCGATGGAGGTGCAAGGGGGC
CGTATCGAGCGGCAAGATTTATGACTCCCATTTGTACATCGTTGCCCTTCGTGCCGAGCCGGGACCCTGCG
GTGATTCGTGTACAAATCGAACAATATCTCTTCAGAGTTGGGGATGATGATCAGTGTCTGAACCAAGCACC
CTTTGTCTCGGGGAACTGTAACGCACCACTTAAAGAGAATGTAGCTTACAGGTTCAAATACGCTGTGCTGA
ACTCCTCCACAAACATCATTTTAAATGAAACGTCCTGGTCCGATCCCATCCCCCTCCTACGAGTCGCTGAC
TTTGCCCTCATTGATACGTGGCCTGGAGCAAGGACGGGAGGAATGGTGGTGATCACAACTCTCCTTGTTAT
CCTTCTCTCTCTTCTCCTGTGCGGTTATGGTGCTTTGCTGGTTTATGCCTGTTG

>UPK3a.Shark JK928319; AAVX01399128.1|PLUS AAVX01255679.1
GAATTCTTCAGTTCAAGCCAGAAATGCCCTCCGTCTCTACCATCGATGGCCTCCGCACGGCCCGCACGGTG
ACCCTCTCCAAGCCGCTGTGCGTCTTGCCCACCGGGCACATGATTGAGCTGCTGGTCGTGCAAGGAAACGT
GACCCCAATCGCCACAAACCTTGATGGCACTTACCAGGCAACTAAAGGAGGGGCAACTGGCCCATACCGAG
CTGCACGTTTTGCCAACCCAGAATGTACCTCTTCCAGCTTCACACCCAGCACAGATCCCACAAAGATTCAA
ACATTGATCGATCGCTATTTCATCCGGGTTGGAAGTGATACCCAGTGTCTGAGCGGAGGGACTGCCCAGTC
GGTGCCCTGTAATGCACCACTGAATGAAAATGTAAACTACAGGTTCAAGTACGTTGCCCGAGACCCAATAA
CCTTCATTCTTAGGGATGAAACCGTCTGGTCAAAACCAATAACTCTTCTGCAAGTTCAAGATCCTGCAAGA
ATTATTACTTGGCCTGGTGCAAGGACTGGAGGAATGGTCGTCCTCACAACCCTTCTGGTTATCATTCTCTT
CCTTCTCCTCTGTGCCTTTGTTGCTTTTCT

>lamprey3a.3 profiling Ensemble scaffold:Pmarinus_7.0:GL480097.
GCGAACGATACGGCAATCTGTTCCCCTCGACGAGCTCTACACGATGCGCTAATCAGTTCCAAAGTTTTGTT
TCCTCTGCGCATTCGTCCTCGCTGCTGCGCTTGTACGACACGACAACAGAACGATCCGCGCCAGGCTACTC
GTTCTCACGCGTGCTCGGAGCCGTGGGGTCGAGGGTGGCCACCGGCGCGGGAGATCGGGGCCCCTACCTCG
CCGGCTACTTCCCCGTGCCCCCCTGCGCGCAGCCGCCGCCGCCCTCCGTGGTGGTGGGCACCGGCGGGTCC
AGCGTTGCCCTGGCCCTCCTCGGGATGGTCATCTTCCGTCTCGGGGGTGACCCGGCCTGCGCCACGGGCAT
CGCCGATGACCCCGCGGCGCTTGTGTGCAACGGCCCCCTCTGGAACGGCACGGCCTACCGAGTGAAGTTCG
TGCTCCTGGCGAGGAGCTCTCTGCTCTCCCCTCCTGCCATGGAGACGCCGTGGTCCGACCCCATATCCACC
AAGCCAGTGGTGAGCGTTGAGCAGATCCACGTGTGGCCCGGCAAGCGCACGGGGGGCATGGTGGTGGTGAC
CTCCGTGCTCTGCTCCATCCTCTTCGTGCTGCTCTTTGCGCTCACCGCCGCGCTGCTCGCGCCCAGC

>lamprey3a.1 FD718834.1 FD706071 FD704820; profiling Ensemble scaffold: Pmarinus_7.0:GL477389.

GCAGCGGCACCAAGCCTCAACTCGTGAGCTCGACGGCCGTTCCGTACAACCCGACGGAGACAACGATCGTG
TGGTCCAAGCCCTTCTGCGTGTTCCAAAAGCCCGTTCCCACCACTCAGTACGTCGTGGACGTCTACGCGTC
CATCACCAACAACAGTTATGCATTCGACAACTCAATCGGCGCGGTGCTCTCCTCCTACTGGACTAACGCGG
TGTCGCCCAGTCCGTACCTGGCAGCCACATTCAAAGTTCCGGACTGTGCAAGTCAACCCAGCATTTACGAC
GCCATGGCTGTCAAAACGAACGCGACATTCCGTCTGGGCGGCGACACGGCCTGCGTGAACAGCATAGGGCC
CAGTACGTCCGTATGCAACGGACCGCTCGTACCCGGAATGAAGTACAGAGTGAAATATACACTTTCGGAAG
AATCTCCCCAATTTCCCAGAACTATTGTGGATCAAACTCCCTGGTCAGACCCTGTCTCCACAAAGAAATCG
CCAGCGGCCAGCACCATCAACACGTGGCCCGGCAAACGCACGGGTGGGATGGTGGTGGTTACCGCCGTCCT
CAGCACGCTGCTCTTCCTGCTGCTCGCCGCACTCCTCCTGGTGGTCATCTTCAAGGCGTGCAG

>lamprey3a.2 FD709144 prolfiling Ensemble scaffold:_7.0:GL483426.
TGTCACAGGTGGTGCCCACCGTGGTGAACCCCAACCTGCTGGGAGCCGTGACGCAGACGACGGTAGCGCTC
CAGGCGCCCTTCTGCAGCGCCCTGGATGCGGAGGTGGTGGCTCTCAGCTCGGTCTCCGCCGACCTGCGGCT
CTTCGTGATGGCCACGGCGCAACGCAACGTGAGTAACGAGATGATCACGAGCTCGTCGACGATTGGGCTCG
ACAAGGGATACGCGGGCTCGGGTGCGGGGACCAGCAGCTGGTACTTGGCTCTGGGAGGCCGCCCCCTACAA
AACTGCACCATCTCCACGCCGTCTCTGTCGACGCCCACCAGCTACTACCGCGTGGGCGCCGACTCCAAGTG
CAGCGTGGCGGTCACGTGCAACGGGCCACTCAATGCCGGCACCATCTACTGGTTCAAGTACATCATGGGAA
CGGTGGCTACCAACGGGGCCATCGACCGCTCTTACCTCGAGTCTTCCTGGTCGAAACCCATCCGGCTGAAC
AAAGCCGGTGAACTCAACGCGATTGGCGTGACCCCTGGGCCGCGNTCGGGCGGCATGGTGGTGGTGACAGT
CATCCTGGTCGTGCTCCTCTTCATCGCGGTTGATCGGGCCCGGCCTGCTCTTGGTCA

>UPK3b.Human NM_182684.1
AGCTGGTGCCCTACACACCACAGATAACAGCTTGGGACCTGGAAGGGAAGGTCACAGCCACCACCTTCTCC
CTGGAGCAGCCGCGCTGTGTCTTCGATGGGCTTGCCAGCGCCAGCGATACCGTCTGGCTCGTGGTGGCCTT
CAGCAATGCCTCCAGGGGCTTCCAGAACCCGGAGACACTGGCTGACATTCCGGCCTCCCCACAGCTGCTGA
CCGATGGCCACTACATGACGCTGCCCCTGTCTCCGGACCAGCTGCCCTGTGGCGACCCCATGGCGGGCAGC
GGAGGCGCCCCCGTGCTGCGGGTGGGCCATGACCACGGCTGCCACCAGCAGCCCTTCTGCAACGCGCCCCT
CCCTGGCCCTGGACCCTATCGGGTGAAGTTCCTCCTGATGGACACCAGGGGCTCACCCAGGGCTGAGACCA
AGTGGTCAGACCCCATCACTCTCCACCAAGGGAAGACCCCCGGATCCATCGACACCTGGCCAGGGCGGCGA
AGTGGCAGCATGATCGTCATTACCTCCATCCTCTCTTCTCTGGCCGGCCTCCTACTCTTGGCCTTCTTGGC
AGCCTCTACCATGCGCTT

>UPK3b.Mouse NM_175309.4
ACCTGATTGCCTACGTGCCGCAGATAACAGCCTGGGACCTGGAAGGGAAGATCACAGCCACTACATTCTCT
CTGGAGCAGCCTCGGTGCGTCTTTGATGAGCATGTCTCAACTAAGGACACCATCTGGCTAGTGGTGGCTTT
CAGCAATGCCTCCAGGGACTTTCAGAACCCACAGACTGCTGCTAAGATCCCGACCTTCCCACAGCTGCTGA
CTGACGGCCACTATATGACATTACCCCTGTCCCTGGATCAGCTGCCATGTGAGGACCTGACCGGTGGCAGT
GGAGGTGTCCCCGTGCTTCGGGTGGGCAATGATTTTGGCTGTTACCAGCGACCCTATTGCAACGCCCCCCT
CCCCAGCCAGGGCCCTTACAGTGTGAAGTTCCTTGTAATGGATGCCGCCGGCCCACCCAAGGCTGAGACGA
AGTGGTCCAACCCCATTTATCTCCACCAAGGAAAGAATCCCAACTCCATTGACACATGGCCTGGCCGACGG
AGCGGCTGTATGATCGTCATAACTTCCATCCTCTCTGCCCTGGCCGGCCTCTTGCTCCTGGCTTTCCTGGC
AGCTTCCACTACGCGTTT

>UPK3b.dog XM_844184.2
AGCTGATCCCCTACACGCCGCAGATAACAGCCTGGGACCTGGAAGGGAAAGTCACAGCTACCACATTCTCC
CTGGAGCAGCCTCGCTGTGTCCTGGACGGGCCTGCCAGTGTTGCCAGCACCGTCTGGCTGGTGGTGACCTT
CAGCAACGCCTCCAAGGACTTCCACAACCCACAGACGCTAGCTGAAATTCCAGCCTTCCCGCGGCTGCTGA
CTGATGGCTACTATATGACACTGCCCCTGTCCCTGGACCAGCTACCCTGTGAGGACCCTGAGGGTGGCGGC
AGGAGCATCCCCCTGCTGCGGGTAGGCAATGACCCTGGCTGTCTTGCTGACTTCTACGAACCACCCTACTG
CAACAACCCCCTCCCCAGCCCTGGACCTTACAGGGTGAAGTTCCTCTTGATGGACGCCAGGGGCTCACCCC
AGGCTGAGACCAGGTGGTCCGACCCCATTACTCTCCACCAAGGGAAGGCCCCAGGCTCCATCGACACGTGG
CCAGGGCGGCGAAGTGGTGACATGATCATCATCACCTCTATCCTCTCCTCCCTGGCTGGCCTCCTGCTCCT
AGCCTTCCTGGCAGCGTCCACTGTGCACTT

>UPK3b.Cow NM_001012672.1
ACCTCATCCCCTACACGCCGCGGATAACCTCCTGGGACCTGGAAGGGAAGGTCACGGCCACCACGTTCTCC
CTGGAGCAGCCACGCTGTGTCCTGGACAGGCACTCCAGCGCTGCCGACACTGTCTGGCTGGTGGTGGCCTT
CAGCAACGCCTCTAGGGTCTTCCAGAACCCCCAGACACTGGCTGAAATCCCAGCCTCCCCACGGCTGCTGA
CCGATGGCCACTACATGACACTGCCCCTGACCATGGACCAGCTGCCCTGTGAGGACCCTGCGGACGGCAGT
GGGCGCGCCCCCGTGCTTAGGGTGGGCAATGACGCCGGCTGCCTTGCTGACCTCCATCAGCCTCGCTACTG

CAATGCCCCCCTCCCGGGCCCGGGGCCTTACAGGGTGAAGTTCCTGCTGACGAACTCCAGGGGCTCACCCC
AGGCCGAGACGAGGTGGTCCGACCTCATTGCTCTCCGCCAAGGGAAGTCCCCAGGCTCCATCGACACGTGG
CCCGGGCGGCGGAGTGGAGACATGATCATCATCACCTCCATCCTGTCTTCTCTGGCCGGCCTCTTGCTCCT
GGCCTTCCTGGCAGCCTCCAGCGTGCGCTT

>UPK3b.elephant XM_003416566.1
CTGATCCCCTCCACGCCACAGATAACGGCCTGGGAGGGAAGGTCACAGCCACCACGTTCTCCCTGGAGCAG
CCGCGCTGCGTGCTCGACGGACATGCCAGGGCTACAGACACCGTCTGGCTGGTGGTAGCCTTCAGCAACGC
CTCCAAGGACTTCCAAAAGCCAAAGACACAGGCTGAGATCCCCACCTTTGCCCAGCTGCTGACCGATGGGC
ACTGTATGACACTGCCCCCGTCCCCAGTCCAGCTGCCCTGCACAGACTCCGTGGGGGGCAGTGGGAGCGCC
CTCCTGCTGCGGGTGGGCAATGATGCCCGTTGCCTGGCTGACCTCCAACAGTCACCCTACTGCAATGCCCC
CCTACCCAGCCCTGGGCCTTACAGCGTGAAGTTCCTCCTGATGGACACTGAGGGCTCACCCAAGGCGGAGA
CGAGGTGGTCAGACTCCATTGCTCTCCACCAAGGGAGGGCCCCAGGCTCCATCGACACGTGGCCAGGGCGG
CGAAGTGGGGGTATGATTGTCATCACCGCTGTCCTGTCTTCTTTGGCTGGCCTCCTGCTCCTGGCCTTCCT
GGCTGCCTCCACCGTGCACTT

>UPK3b.opossum XM_001378848.2
ACCAAATCCCGTATACGCCGCAGATCTCCGCGCTGGCCCTGGAGGGGAAGGTCACTGCGGCCACGTTCTCT
CTGGAGCAGCCCAGATGCATCTTCTCAGAGCTCGCCGCCCCGGCTGATGCTGTCTGGCTGGTGGTCGCCTT
CAGCAATGCCACGGAGGATTTCCAGAACCCTAAGACTGCCGCAGAGATCCCCTCCTACACTGAACTGTCCA
GCAGCTTTTACTACATGACCCTGAAGCTGTCTCCTGACCTCTACCCGTGTGAGGAAGAAGACATCGCCGTC
CTCCGTGTGGGCAGTGACACCAACTGCCTCCGAAACCTGAGCCAGGAGTACTGCAACGCTCCCCTCTTGGC
CCCTGGTCCTTACAGGGTGAAGTTTCTGGTGATGGACAACAATGGCCAGCCCAAGGCAGAGACGTGGTGGT
CGGACCCAATCACTCTCAACCAAGGGAAGGACCCCAGAAGCATTGATACTTGGCCGGGCAGGAGAAGTGGC
TGCATGATCGTCATCACCTCCATCCTCTCCACCTTTGCGGGCCTCCTGGTGATCGCCTTCCTCATCGCCTC
CACCGTCCAGTT

>UPK3b.platypus profiling gi|125702340|gb|DS191120.1| Scfld14595
AGCTGATGAGCTACAAGCCCCAGGTGTCCGCTCATCCCCTCGAGGGCAAGGTTACTGCCAGCACCTTCACC
CTCGACCAGCCGCGCTGCGTCTTTGACGGCCAAGTCGTCTCCACCGACACCATCTGGTTGGTGGTGGCCTT
CAGCAACGCCTCTCGAGATTTCCAGAACCCGACGACGGCGGCCTCCATCCCCGCCTACCCCAGGCTGCTGA
CCGACTACTATTACATGACGCTGAAGGTGTCTCCGGATCTGTACCCGTGCGCCGACGCCGGCGGCCTCTCC
GTGCTCCGGGTGGGCACCGATCCGGGCTGCCTCCGAGACCCGGGCAGAGAGTACTGCAATGCCCCACTTCC
TGCCCCCGGGCCTTATAGAACCAAGTTTCTCGTGATGGATGAGGCAAGCCACCCCAAGGCAGAGACGCAAT
GGTCTGATCCAATCACCCTCAAACAAGGGCGAGACCCCAGCTCTGTGGACACGTGGCCCGGCCGGCGTGGA
GGCAGCATGGTCGTCATCGCGTCCATCCTGTCCGTCCTCGCCGGCCTCCTTCTGCTCGCACTCCTCACTGC
CGCTGCTGGAGCCTGCACAGT

>UPK3b.chiken profiling chromosome: WASHUC2:19:4127260:4157406:-1
CCCTGCTGCCCTACGTGCCCCGCGTGGCCCCCGGTGCCATGCCGGGGAAGGTGACGGCCACCACCTTCGTG
CTGGAGAGACCCCGCTGCATCTTCGACCCCTTTGCCAACGCCTCCGATGCCGTCTGGCTGGCGGTGGCTTT
TGCCGACGCGCCGAGGTGCCCCCATGACGAGGGGCTGCCTGCCGCCCGCGCCTACATGACACTGCAGATGG
CGGCAGCCGCCTACGGATGCTCAGCTCCCGGTGCAGCCGTGCTGCGGGTCGGCGGGGACACGGCGTGCCAC
GGCCGGGCACCCTGCAATGGGCCGCTGCCCTCCCCGGGGCCCTACAGGGTGAAGTTCCTGCTGATGGGCTG
CGGTGGCCCCAAAGCGGAGACGAAGTGGTCCGACCCCATCCTCCTGCGGAGAGCTCGCAGCCTGAGCACCA
TCGACCCCACACCCGCACGTCGCAGCAGCACCGCGGTCGTCATCGCCGCCATCCTGGCCAGCCTGGGTGCC
GCGCTGGCCATGGCCGTGCTGGGAGCTGTGGG

>UPK3b.aligator profiling gi|397229275|gb|AKHW01080724.1 scaffold-
9559_4
AGCTCGTGCCCTACACGCCNCAGATCACAGGCAACTCCCTGGAAGGGAAGCTCACAGCTTCCACCTTCAGC
CTGGACCAGCCCATCTGCATCTTCGATCAGTATGTGAACGCCACTGATGACATCTGGCTAGTTGTTGCCTT
CGCTAATGCAACCTCCAGTTTAAAAAACCCAACATCTCGCACTGACATCCCACCTTACCAGCGGCTCTCGA
CTGCTCTCCATTACNTGACCTTGAGGACTACCATCNCCTTCTTCCCATGTCCTAGGAGCACCAACACAAGC
GTGCTCCGAGTAGGCAGTGATGCTTTCTGCAGGAACGACAACAGCCAGCAGCACTGCAATGGACCCCTGCC
CAACCCCGGGCCCTACAGAGTGAAGTTTCTCATCCTGGATTCCAATGGCGCCAAGGCAGAGACAAGATGGT
CTGAAAGAATCACTCTGAAGCAAGGCCACAGGTCAAGCACCATTGACACCTGGCCAGGGAGGCGCAGCGGC
ACCATGGTTGTCATCACCGTCATCCTTTCCAGCTTAGTCGGCATCCTGACCATTGCATTCCTCTGCACAAG
CGCCTATGA

>UPK3b.Xl BP703707 BJ034026

ATATCACTACATATGTTCCACAACTGACCCTTATGCCTATTCAGGGCAGCGTGACAAGTACAACATTTACA
CTGGACAAACCACAATGTATATTTGGTAGCAGAACCAATCAGGTTTGGCTGCTTGTTGCTAGAAGCAATGT
GTCAGTGTCTATAACAAATGCAATGCTCAAGCCGCCCTCTATGTATTCATCATTCCCGACTCAAGGATACT
ACCATGTTCCCCTTGGCACCGAGGCTTCATACCCCTGCTCTAACACAGCTGACTATATTAGAGTTGGAGAT
ACTGTCTATTGCACAGATAATACATATTGCAATGCACCCTTACCTGATTCTGGACCTTACCGGGTGAAATT
TGTCGTGATGAACAATAATGCTTTGGTCTCATCGTCACTTTGGTCTGGTTTGATAACTTTGCGGACAGGGA
AGAATCCATCTACAATTGATACTTGGCCTGGGAGGAGAAGTGGGGGAATGATTGTTCTAACCAGCATTCTC
TCACTTCTTATGGGAATTTTGACACTCTGCCTAATTGCTGCTTTCTTTGTTGGATG

>UPK3b.Xt EL866947; Ensemble scaffold:JGI_4.2:
GL172708.1:135056:150415:-1
ATATCACTTCATATGTTCCACAATTGACCCTCTCGCCTATCGTGGGCACAGTAACAAGTACAACATTTGTA
CTAGACAAACCACAATGCGTATTTGGTAACACAGGGAATCAGGTTTGGTTGCTTGTTGCTAGAAGTAACGT
GTCAGCGAATGTGGTGCTCACTCCACCCTCCATGTATTCATCATTTGCCACTAAAGGATACTACCATGTTC
CCTTTGGCACTGAATCTTTATACCACTGCTCTAACACAGCTGAATATATCCGAGTTGGAGATACTGCGCAG
TGTAATGATAATACAAACTGCAATGGTCCCTTACCTGATCCTGGGCCTTACCGGGTGAAATATCTTGTGAT
GAACAATAATGCTCTAGTCTCACAATCACTTTGGTCTCAACAAATAACTTTGCTGACAGGGAAAAGTTCAT
CTCAACTCGATACTTGGCCTGGGAGGAGAAGTGGAGGAATGATTGTTCTAACCAGCATTCTATCAGTTCTT
ATGGGAATTTTGACACTTTGCCTATTTGCTGCTTTCTTTGTTGGATG

>UPK3b1.Xtropicalis profiling Ensemble scaffold:JGI_4.2:
GL172728.1:2871266:2881345:1
ATATCCCTTATTATGTTCCCCAGATCACAACAAAGCCAATTCTTGGAAAACTGACATCTTCAAGTTTTGTT
CTGGAGCAGCCACAGTGCATCTTCCAGCAGTACAAAACAAGTCTGGTCTGGTTGGTTGTTGCTCTAAACAG
AGTCATTCCACAGCTCAGTTATACACAGTTGAGTAATCCAGCCAACATTTCATCATTCGAAACAAATGGAT
TTTATCACACTCTCCCTGTATTTGGAGGTGACTACCCATGTGCTGATACATCTGGACAACTTAGTGCAATG
ATATATGTGGGCTCTGATGTAAACTGCAGTAACCCTTTATTCTGCAATGGCCCTGTGCCCTCTCGTGGAAC
CTACCGAGTAAGATTTGTTGTGCTGAATGGGACAGTAATGGAGACAGGAACGCGTTGGTCAGAAGTAATTA
CAATGCACATAGCAATCAACTCATCTACGATTAAAACAGAGCCAAAAAGGCCTAGTTCCGGAATGATTGTC
ATTATCACTATACTTGCGAGTCTTCTGTTTATTTTATTGGTCTGCCTCATTGCCGCTGTTTCACTTGGAAG

>UPK3b2.Xtropicalis XP_002934406.1; EL802701; Ensemble scaffold:
JGI_4.2:GL172728.1:2884693: 2898749:1
ATGTTGGCAGCTACGTTCCAAAGATCACAACATCGATTCTAGGGAATTTAACATTCTCAACTTTTGTCTTA
GAGCAACCACAGTGTATCTTCAGCTCTAACTATCCTACACAAGATGTCTGGTTGGTTGTTGCTCTTGATAC
TGTTGAGCCCTTTTTAACTGATACAAATCTGAGTACCCCAGTGACTTATTCTTCATTTACAACTAATAAAT
TCTACCATACTCTCCGCGTCCGTGGAGCTGACTATCCGTGTTTCAATGAATCTGCGATGTCCTTAGCGTTG
CTTCAAGTGGGAGCTGATGAAAAATGCAATGAATCATTCTGCAATGGTCCTTTAACTTCTCCAGGACCTTA
CCGGGTGAGATTTGTTGTGTTGAACAACACTGGTATGGTGGCCAAAACCAACAGGTCGGATTTAATAAGAC
TGCCAATAGGAATAAATTACACAACGATTGACACGTGGCCTCTGAGTCGTAGTGGGAGTATGATTGTAATT
ACCACCATACTTTCCATTCTTCTTGCCGTTTTGCTGGCCTGTCTCTTAGCTGCTCTCTGTTCTGAAAG

>UPK3d.coelacanth profiling AFYH01004438 gi|346210398|gb|.1|
contig004438ACGTTCCCTACCTTCCCGAGGTTGCATTTAACAACGTGGCTGGAAGTGTTACAGCCACC
ACCTTCACCCTGCAGCAGCCTCGCTGCATCTTCAAGGATGTGTTCAGTTCATGTCCTTTGTGTCAACTGTG
GGTGGCGGTTGCTACAGAAAAGGGTGCGAACAATTTAATAACAACAGTAGGCCGTCCAGTAACATTTGCTG
GTTACCAGAAATTATCTCAACGGGGCTATTACTTTACTATGAGGTCGAGCAAAGATTTGTACAAGTGTGTA
GAGAATGATGACAAGGTGAGAGTTCTACGCCTAGGCGCTGACACCAGCTGTCCTCAAAGCATCGGCGAATC
AGACTGCAATGGACCACTGCCTGACAGCGGATCCTACAGGGCAAGGTTCCTCATTGTTGATTCTCAATCTG
GCAACATGCTCAAAGCACAGTCCCAATGGTCCAAGTCAATCCAGCTCAAAAGACCCAAGGACCCGGCCAGT
ATTGACACTTGGCCGGGTAAACGGACAGGTGGAATGGTTGTGGTCACCACTATCCTTGTCATTCTCCTGGC
GATCCTGCTCTTGCTCTTCATAGTGGCTCTCATTACTGCATG

>UPK3d.1. Spotted Gar genomic profiling AHAT01005644.1 contig005644
TGACTTCAGTGGAGTACACCCCAGAGATCACCGCCTACAACATGGCTGGAAGGGTTACTGGCACCACGATG
GTAATGAAACAGCCCAGGTGCTACTTTGACAACCAGCTCCTTCTGCCCTGCACCCCAGATAAATGTGAGAT
ATGGCTGGTGGCCGCTGTAGGCTCAGCAGGAATCCAAAACTTCGATGCAGACAAGGGAAAATCCACCATCC
TCAGTGAATCTCCCTATCCCACTGCTTTCACCGGCAGCCCCCCTAAGAACTACTACCTGACGAAGGTGGGA
CGTCAGCAGGTATTCCCCTGTCAGCAATCCCATGGGATTGTATATTTCAGAGTCGGGGATGAAGGAAACTG
CACCTCGGCCAACTGCAATGGAATATTGCCGGCTGGCTCCACTGTCAGGGTGAAGTATGTTCTCGTTGACC
CAGCGAGCAGAAATGTGACCACAGAAACCTATTGGTCTCAAAATATTACTCTTTACAGCTCGACGGATCCA

GTGGTCATCTTTGATGGAATCAGGCAACGCTCTGCAGGGATGATTGTCATCACCTCTATCTTGAGTGTCCT
TCTCTTCCTGCTGCTGCTGCTGCTCATTGCAGGCTTGATCTATGCCAA

>UPK3d.salmo BG936628; AGKD01191862.1| Contig_191888
TTGTGTCTCTGGATCATATTCCTGAGATTCTCCCCTATGAGGCGAGCGGCAGGGTAACCAGCACTACCATT
GTGCTAGGCCAGCCTCTGTGCTACTTCAACACACTGACCCAGCTCAAATGCAGTCAAAGCACCTGTCAAGT
ATGGGCTGCCATCGCCTCTGGACCAGGCATCAACAACTTTGACATTGACAAGCTGGTAGCAGTACAGATCG
TCAGTGCCTCTCCATACCCTATTGCCTTCAGCAGCCAGACTAATAGAATGTACTTTGTCACCAAATTGGGT
CGCCCTAAGGACTTCCCCTGTGGCCAGTTGCCTGGCATCAAGTACTTCAGAGTAGGTGCGGAGGGCAACTG
CACCAACACCAACTGCAATGGGATCCTACCTCCGGGTTCCACTGTCAGGGTGAAGTACATCCTCATTGATC
CAGTCTCTAGAGGAGTGGTCTCAGAGTCTAAATGGTCCTATCCCATTTCTCTAACATCAACGAGGTCCTGG
TCCTCCATAGATGAGTGGTTAGGGAAGCGCTCTGGGGGAATGGTGGTCATCACAGTCATCTCTTCCTGTCT
GCTGGCTGTCCTGCTACTCCTGCTGGGAGCTGTGCTGCTACTGGACGG

>UPK3d.trout CX140558, CA369219, BX073041, CX140559.
TTGTGTCTCTGGATCATATTCCTGAGATTCTCCCCTACGAGGCGAGCGGCAGGGTAACCAGCACTACCATT
GTGCTGGGCCAGCCTCTGTGCTACTTCAACACACTGACCCAGCTCAAATGCAGTCAAAGCACCTGTCAAGT
ATGGGCTGCCATCGCCTCTGGACCAGGCATCAACAACTTTGACATTGACAAGCTGGTAGCAGTACAGATCG
TCAGTGCCTCTCCATACCCTATTGCCTTCAGCAGCCAGACTAATAGAATGTACTTTGTCACCAAATTGGGT
CGCCCTAAGGACTTCCCCTGTGGCCAGCTGCCTGGCATCAAGTACTTCAGAGTAGGTGCGGAGGGCAACTG
CACCAACACCAACTGCAATGGGATCCTACCTCCGGGTTCCACTGTCAGGGTGAAGTACATCCTCATTGATC
CAGTCTCCAGAGGAGTGGTCTCAGAGTCTAAATGGTCATATCCCATTTCTCTAACATCAACGAGGTCCTGG
TCCTCCATAGATGAGTGGATAGGGAAGCACTCTGGGGGAATGGTGGTCATCACAGTGATCTCTTCGTGTCT
GCTGGCTGTCTTGCTACTGCTGCTGGGAGCTGTGCTGCTACTGGACGG

>UPK3d.Zebrafish A2CE76 DT080652.
GGTCAGATATTCCAGCC
TCAGCTTGCACCTGCTAATTTCCTAGGCCGAATCACTAGCAATACAGTGATCCTGCAGCAGCCGTACTGT
GTTTTTACTCAGACCTGTCCTGGGTGTGAGATCTGGCTGGTGGCTGCGCTGAGCACGGGAACGGGCAACT
TCAACGCTCTAGTGAACATCTCATCCCCCATCAGCCTCAGTGTGTCACCATACCCAACAGCTTTCCTCCC
ATCATCAGCACAGTTCTTTCTGACCAGAGTTGGTCCTCTGGCAAACTTCCCCTGTAATACGGCCCCTGCT
TTTCCATACTTCACAGTTGGAGCGGATGGGATTTGTACAGGCATCAACTGTAATGGAGTACTACCTGTTG
GATCCATTGTTAGTTTCAGGTATCTTCTTATCGACCCCAGCAACTATACTGTTGTTAATATGACCAACTG
GGGTGGCCCCTTTAATCTAACCACATTACTGAGCTATCAAACCATCAATGACGGTCTCAGTGCCAGATCT
GGAGCCATGGTTGTCATCACTACACTCCTCTGTGTGGCCGGGGCTCTGCTTTTGCTGGTTTTTCTCATCA
TGCTGTGTGTATCCTGC

>UPK3d1.salmo profiling AGKD01066640.1|
Contig_066647TACCAGTCAACAACACTCCTGAAATAACCCCTTCCAGCTTGGCTGCGAAACTGACCTC
TAATTCAGTGATACTGACGTCCCCAAGTTGTTACTTCAACGGTCTGGGCAATTTGTCCTGCAATTCTACAA
CTACCTGTGAAATATGGCTCGTTTCGGCCAAAGATACAGGAGTCAGCAATTATGATGCCGACAACAAAATG
CCCTACATTGACACCCGCTCTCCTTATCCAACCGCCTTCTCTTCCAACACCTCGAAGAAATACTTCCTGAC
CAAGTTGGGGCTTCAGAAAGCCTACCCCTGCCCCATCGTCGCTGGAACAGGCTACTTCAGGGTTGGCTCTG
ACGGTAACTGCTCCACTCCGAACTGTAATGGAATATTGCCTGTTGGCTCCACTGCCAGATTTAAGTATGTT
CTTATCAACCCAGCGAACAAAACAGTTGTTGCGGAGAGTTTGTGGTCCAACAACATCACTCTATACTCTTT
GAAAGACCCCGAAAAAATTGATAATGGCTTTGCTGGAAGGTCTGCGGCCATGATTGTCATAACAGCTATCC
TGTGTTCCTTCCTGGCCTTGCTGCTGCTGCTATTGCTCATCATGCTGATCTATGTCCT

>UPK3d1.trout CX145617, CX153372.
TACCAGTCAACAACACTCCTGCAATAAACCCTTCCAGCCTGGCTGCGAA
ACTGACCATTAATTCAGTGATACTGACGTCCCCAAGTTGTTACTTCAACAGTCTGGCCAATTTGCCCTGC
AATTCTACTACTACCTGTGAATTATGGCTCGTATCGGCCATAGATACAGGAGTCAGCAATTATGATGCCG
ACAAGAACAGGCCCTTCATTGACACCCTCTCTCCTTATCCAACCGCCTTCTCTTCCAACACCTCAAAGAA
ATACTTCCTGACCAAGTTGGGGTTTCAGAAAGACTACCCCTGCCCCATCGTCGCTGGAACAGACTACTTC
AGGGTTGGCTCTGACGGTAGCTGTTCCACTCCGAACTGTAATGGAATATTGCCTGTTGGCTCCACTGCCA
GATTTAGGTATGTTCTTATCAACCCAGAGAACAAAACAGTTGTTGCGGAGAGTTTGTGGTCCAACAACAT
CACTCTTTACCCTTTGAAAGACCTTGAAAGCATTGATCATGGCTTTGCTGGAAGGTCTGCGTCCATGATT
GTCATTACAGCTATCCTGTGTTCCTTCCTGGCCTTGCTGCTGCTGCTATTGCTCATCATGCTG
ATCTATGTCC

>UPK3d.SpottedGar genomic profiling contig005644, Sequence ID: gb|AHAT01005644.1|
CAGTCCCCTCAGTGGACTACGTGCCAGAGATCATTCCCATGAAACTGGCTGGCAGGCTCACCGGTACAACC
TTCCTGCTGAGGCAGCCCCTGTGCTACTTCAGCAACCAGCAGGGCCTGAACTGCAGCCTCAGCACCTGTGA
GATATGGCTAGCTGTTGCCAGAGAAGCAGGAGTTAACAATTTCGATACGGACAAGGTCCAGCCCTCGTTCG
ACATCGTCAGCGCTTCTCCATATCCCGAGGCCTTCCAGAACAAGAACTACTACGTCACCAGACTGGGGGTC
CAGAACAACTTCCTCTGTGCTGAGCTCCCCGGCATCAGGTATTTCCGAGTGGGAGCTGAAGGCAACTGCAG
CACTCCCACCTGCAATGGAATTCTGCCTGCTGGCTCCACTGCCAGCTGTGCTGTCCTGGGGTTGGCTGTTG
TCAGTCAGCTTCAGGATCAGCTGGTTAAAGGGGCTCTTCTCTGGGCTCAGCAGGGCCTTGTGGTGGTAGAT
GTGATGCCCAGCTCCTCCATTGATGATTGGACATGGAAGCGCTCTGGAGGGATGGTGGTCATCACAGTCAT
CACTTCCTGCCTGCTGGCCATCCTGCTGCTGCTGGTGGCAGCGCTGCTGCTGGGCGG

>UPK3d.2 Spotted Gar genomic profiling |AHAT01005643.1 contig005643
TGCCGTATGCCCCAGAAGTCACTCCCCACAATCTGCTGGGCAGAGTCACCAGCACAACCCTCACACTGCAA
CAGCCTGTGTGTTTCTTCAACAACCTCAGTGGCCTCCCCTGTGCTCCAGATAACTGTGAAATCTGGCTGGT
CATTGCTAGAGGAGTCCCAAAATTTGAAGCCATTAAAGGAAACACCAGTGTCTTGATGAGCTCCCCCTATC
CAGATGCCTTCAGAAATAACTCCTCACCTAATTACTTCTTGACCAAGCTGGGTGTTCAGAATGCGTTTCCC
TGTGCTGAGTCCACAGGCTTCAGGTTTTTCAGAGTTGGGGATGAGGGCTTGTGCTCCACCAGCAACTGCAA
TGGGATCTTGCCTGCTGGCTCCACTGTCAGAGCAAAGTATGTTCTCCTGGATCCTGGTTCAAAACAGGTGG
TATCTGAGTCACAGTGGTCCAGTCCCATCTCTCTGATAGCGTTGAGCGACTCTGCTGGTATTGATGAGTGG
ATCGGAAAGCGATCGGGAGGAATGATCGTGGTGACGTCCATCCTGTCCTCTCTGCTTGCCATCCTGCTGCT
CTTTCTGCTCACGGCCTTTGTCCTGAGATG

>UPK2b.Shark JK941564, JK956747; AAVX01097325.1| scaf_1099306956075,
CTGGTTGTAGACTACGACATCGCTCTGTTCAACGGTGAGGTGTCGCGGTTAGCCACGCTGGTCTGGCTGA
GCCCGGCCTACTGCCTCTTCGAGAAATGGGTTGAGCAGAGAGGGGGTGAGCTGACCATGCGATCATCTGCC
TCCGTCCAAGTGGAGCTCCTACGAGAAGATAATGCCACATTCGTCCTGCCTCAGAGGTACAGCGTCCCT
TTTTGCAACGACTTCTCTCCCAAACCCGCAGCTGTGTCCACGCCACTCGCCTACCAGATGGGACCCAATG
TGGCCTGCATAGACGGCAGCTGCATTGAGCATGTGTTGCCAGGACAGAGGTTCAGGGTTCGCTACATCAT
TTACAGTGTGTCCGAAGAGGCCCTGGTGACCACCAAGTGGTCTGCACCCATAGCAACCAGAGACGACCCT
CCAAGTTACTTTTCCATTGACGCAGACACCACCCCTCGCTCTGGCGCCATGGTGGTGATCACTACTGTGT
TGGTGGTCGCTCTGTTTCTCTTGCTTCTCGGGTTTGTCAGCATGCTCGCGGCTCGCTCGAAATTCGCCAG
A

>UPK3d.Skate EE991990; AESE010072473.1| _WGS_1_CONTIG_72535; AESE012558708 WGS_1_CONTIG_2721782; |AESE011082469.1| Leucoraja erinacea LER_WGS_1_CONTIG_1092019; AESE012622692.1 _WGS_1_CONTIG_2785766; AESE010900346.1 _WGS_1_CONTIG_907428, AESE012509864.1|WGS_1_CONTIG_2672938
CAGACGAGGCCGATTTTGTTCCTGAAATATTTAAGGATAATGTCATAGGAGGAGTGAGCCAGACCACATTT
GCACTTCAACAGCCACAGTGTGTGTTTGATAGATTTCAACCTGCCTGCACCCTCTGTGAAATTTGGTTGGT
CGTGGATAACCCAGCAAATGTTTCAACTTTTGATACCAACATGAATACCCTGACTCCCTCTGCAGCCAAGT
ATACAGATTTTATGTTGAATGGTTTTTATCTCACCGTTAAGACAGCGAGGAGTGATTACGCATGTCCGCAA
ACCATGGGCCAAGTGTACACCTTGCGAGTGGGGGATGAGGATCCTTGCACAACCCCCAACTGCAACGCACC
CTTAAATGCTGGCTCGTTGGTCAGAGTGCGATATGTGATGATTAATCCACTTGCAACTACTAACAACGTTA
TTGCAGTAACAAAATGGTCCAACCCCATCCAACTCGAGAATGCCGTCGATCCAAATATCATTGACACCTCT
ACACGTCGGAGTGGAGCGATGGTCGTAATAACCACAATACTCTCTATTCTGCTGTTCTTGCTGCTGGTGCT
GTTTATAGTCATGCTCGCTTT

>UPK3c.Humanref| NM_001114403.2|
CTGCCCCAGAGCACATCAGCTATGTGCCCCAGCTCTCAAACGACACCTTGGCGGGGAGGCTCACCCTGTCC
ACCTTCACGCTGGAGCAGCCTCTAGGCCAGTTCAGCAGCCACAACATCTCTGACTTGGATACCATCTGGCT
GGTGGTGGCCCTCAGCAACGCCACCCAGAGCTTCACGGCCCCACGGACAAACCAGGACATCCCTGCTCCTG
CCAACTTCTCCCAGAGGGGCTACTATCTCACACTGAGGGCCAACCGGGTGCTGTACCAGACCAGAGGCCAG
CTCCATGTCCTCCGCGTCGGCAATGATACCCACTGCCAACCAACAAAAATTGGCTGCAACCATCCCCTACC
AGGACCCGGCCCCTACAGGGTGAAGTTCCTGGTGATGAATGACGAAGGACCCGTGGCTGAAACCAAGTGGT
CCAGCGACACTCGCCTGCAGCAAGCCCAGGCACTTCGGGCTGTCCCCGGCCCCCAGAGCCCGGGCACCGTG
GTCATCATCGCCATCCTGTCTATCCTCCTGGCCGTCCTCCTCACGGTCCTCCTGGCTGTGCTCATATACAC
CTG

```
>UPK3c.mouse  NM_027158.1|
AGAGCATTAACTATGCCCCCCAGCTCCTCGGGGCCACCCTGGAGGGGAGACTCACACAATCTACCTTCACG
CTGGAGCAACCCCTGGGCCAATTCAAGAACGTCAACCTCTCTGACCCAGATCCCATCTGGCTGGTGGTGG
CTCACAGTAACGCCGCCCAGAACTTTACCGCCCCACGGAAGGTAGAAGACAGGCATGCCCCTGCCAACTTT
GACCGCAATGGCTACTATCTCACGCTGAGGGCCAACCGGGTACACTACAAGGGTGGCCAGCCTGACAGCCA
GCTCCGAGTCCTCCGTGTTGGGAATGACAACAACTGTTCCCTGGAGTCCCAGGGCTGTAACTCGCCTTTGC
CAGGGGCCGGCCCCTATAGAGTGAAATTCCTGGCGATGAGTGCCGAGGGACCCGTGGCTGAGACACTGTGG
TCCGAGGAGATCTACCTGCAGCAAGCCCAGACATTCCGAGAAGCTCCAGGGTCCCAGGGCAAGGGCACTGT
GGTCATCATTGCCTTCTTGTCAATCCTACTGGCCATCCTGCTTGTGGTCTTCCTCGTACTGGTCATATCCG
CTTG

>UPK3c.dog XM_844944.3
AGCACATCAGCTACGTGCCCCAGCTCTCAAACCGCAGCCTGGCAGGGACACTCACCCAGTCCACCTTCACA
CTGGAGCAGCCCAGGGGCCAGTTCAGTCACCGCAGCATCTCTGACTCTGATGCCATCTGGCTGGTGGTGGC
CCACAGCAACGCCACCCAGAACTTCAGTGCCCCACAGAGGGTGGAGGACATCCCTGTCCCTGAAGACTTCA
CCCGGAGGGGCTACTACCTCACACTGATGGCCAACCGCTTGCTCTACCCGGGCAACCAGCCAGGCAACCAG
CTCCGGGTCCTCCGCGTTGGCAATGATACCAGCTGCTCCCCCACCAAGAGGGGCTGCAACCACCCCCTGCC
GGGCCCAGGCCCCTACCGAGTGAAGTTCCTGGTGATGAGTGACAAGGGACCTGTAGCTGAGACAGAGTGGT
CCAACGAGACCCACCTACAGCGAGCTGAGAGGCTGCAGGCTGCTCCAGGCCCCCAAAGCACGGGCACCGTG
GTCATCATTGCCATTTTGTCGGTCCTGCTGGCTGTCCTCCTCACTGCCCTCCTTGCTCTGCTTATCTACAC
CTG

>UPK3c.Cow NM_001168011.1|
AGCGCATCAGCTACGTGCCCCAGCTCTCGAGTGCCACCCTGGCAGGGAGGCTCACCCAGTCCACCTTCACA
CTGGAGCAGCCGCGGGGCCAGTTCAGCCACCCCAGCATCTCCGACTCTGACGCCATCTGGCTAGTGGTGGC
CCACAGCAACGCCACGCAGAAGTTCACGGCCCCACAGAAGGTGGAGGACACCCCGGTCCCTGCCGACTTCC
CCCAGCGGGGCTACTACCTCACACTGAGGGCCAGCCGGGCTCTCTACCCCGGCGGCCCGCCCAGCAACCAG
CTCCGGGTCCTGCGCGTCGGCAACGATACCCGCTGCTCCCCGAGGACAAGAGGCTGCAACCGCCCACTTCC
GGGCCCCGGCCCCTACCGAGTGAAGTTCCTGGTGATGAGTGACAGGGGACCCATGGCTGAGACAGAGTGGT
CCAGTGAGACCCGCCTGCAGCAAGCCGAGGTGCTCCAGGCTGCCCCAGGGCCCCAGACCGCAGGCACTGTG
GTCATCATTGCCATCCTGTCCGTCCTGCTGGCCGTCCTCCTCGCTGCCCTCCTCGCCCTGCTCATCTTCAC
CTG

>UPK3c.elephant  profiling gi|253616662|gb|AAGU03064090.1cont3.64089.
AACTCATCAGCTATGTGCCCCGGCTCTCAAACGCCACCCTGGCAGGGAAGCTCACACAGTCCACCTTCACG
CTGCAGCAGCCTCGGGGTCGGTTCAGTCACAAAATCTCTGACTTTGATGCCATCTGGCTGGTGGTGGCCCA
CAGCAATGCCACCCAGAGCTTCATTGCCCCACAGAGGGTGAAGGATTCTCCTGCCCCTGCTGACTTGCCCC
TGAAGGGCTACTACCTCACACTGAGGGCCAGTCGGGCGCTCTACCCGGGCGACCAGGCTGGTAACCAGCTC
CAAGTCCTCCGAGTGGGCAATGACACCCGCTGCTCCCTAACCACAAAGGGCTGCAACCACCCCCTGCCAGG
CCCGGGCCCCTACCGGGTGAAGTTCCTGGTGCTGAGTGATGACAGGGGGCTGGTGGCTGAGACAGAGTGGT
CCGGTGAGACCCACCTGCAGCGAGCCGAGGTACTCCAGGCTTCCCCAGGGCCACAGACCGCGGGCACTGTG
GTCATCATCACCTTCCTGTCAGTCCTGCTGGTCGTGCTCCTCACCGCCCTCCTGGCCCTGCTCATCTACAT
CTG

>UPK3c.opossum XM_001378809.2
AGCCCATCAATTACACCCCAGCCATCACCAGGGAGCCCCTGGAAGGGAGCATCACGAGCTCTACCTTTACG
CTGGACCAGCCCAACGACCAATTCAACGGCTCGGGCATCAGTGACTTGGACGACATATGGCTCGTGGTGGC
CTTCAGCAATGCTTCCCAGAGCTTCGAGCCCCCCCAGTCAGCCCAAGACATCCCCTACGCGGCAACATTCC
TGGATAAAAAGTACTACCTGACCATCCGGGCCAGTCGGGACCTCTACTCCAGTAAGAGAGGCAGCCAAGGC
ATCAGCGTGCTCAGGGTGGGCAACGAGACCAATTGCACCAGGAGCGACTGCAACAAGCCTCTGCCGGGTCC
TGGTCCTTACAGGGTAAAGTTCCTTGTGATGAATACTAATGGCCCAGTGGCAGGAACGAATTGGTCCGAGG
ACATCACCCTGAGGAAACCGGTGGAGTTCTCTGAGTCCCGGCCCCCCAGCAAGAGTGCAGGGACCATTGTC
ATCATAGCAATCCTGTCCATTCTGCTCAGTCTCCTGTTCCTTGCTCTTGTGGCTCTGCTTGTCTATACATG

>UPK3c.platypus exons 2-5
AGAAGATTGCTTACACTCCGGTCATCACGAAACTCCCCATGGAGGGCAAGATCACCTTGTCCACCTTCACC
TTGGACCAGCCCCTGGGCCAATTCAACAGCAGCGCCGTCGACGACTTGGATGACATCTGGCTGGTGGTGGC
CTACAGTAATGCCACGGACAACTTCTCGAACCCTGTGAAGCCCGATGAGGAGTACAACCTCTCAGAACTCT
CCAAGAAACAGTATTACATGACGATGAGGGCCACCAGGGACCTATACCCCGGGGGCAATAACTCCAGCTTC
CTGCACGTGCTCCGGGTGGGCCAGGAGGCCAACTGCACCTCCAAATCATGCAACGGCTACCTGCCGGGGCC
```

CGGGCCCTACAGGGTCAAGTTTCTGGTGATGAATGAGAGAGGGCCTGTGGCAGAGACGGACTGGTCTGACA
ATATCGCTCTGAGAGAAGCCAAGGACCCCGGCGACCCCAACCCCACGAGGCGCAGCACTTTCATGATCGTG
ATCGTCACCATCCTCTCCGTGCTCTTCGCTCTCCTGCTGGGTGCCCTCACCGGCGTTCTCATCCAGGCATG

>UPK3c.Lyzard profiling gb|AAWZ01050405.1| cont1.50404; whole genome shotgun sequence REVERSE
TGTCCGTGAACTACACCCCACGCCTGGCTTCTGAGAATCTGGGAGGGAAGGTCACCGCCTCGACCTTCACC
CTGGACCAGCCGCGCTGCGTTTTCAATGACGTGGTCAATGCCACGGACGGGATCTGGCTGCTGGTTGCCCG
CAGCGACGCCGCCAGGAACTTCACCCGGCCGGGCTCCCCAAGCGAGCTGCCCTTCCAGGACCTGGAGAAGA
ACGGCTTGTACCTGACGCTGAACACGGCCCCGGCCAGCTACCCGTGCCCGGAGCCGGGCGCAGCAGGGGGG
CCGCTGACCGTCCTGCGGGTGGGCAACGAAGTCCAGTGCGCCTCCAACCGTGCGAGGCCAGACTGCAACGG
GCCGCTGCCCAGACCGGGGCCCTACCGGGTCAAGTTCCTGGCCATCAACCCCGACGGGGTGACGGCGGAGT
CGGAGTGGTCGGAGGAGATCGCCCTGGTCCAAGCGCAGAGCCCGGAAACCATTGACGTGTCTCCGGGGCGG
AGGAGCGCCTCGGCCATCGCCATCGCCTCCCTCCTCTCCATCCTCTGCGCCGTCTTGCTGGCCGCCCTGAT
CGCCGCCCTGGTCTACAAATA

>UPK3c.chiken profiling ref|NW_001471508.1|Gga19_WGA256_2
ACAAGTTGTCCTACAAACCCACCCTGGTTGGTGGCAACGTGGAGGGCCGTATGACGGGCAGCACCTTCGTG
CTGGAGCAGCCCCGCTGCGTCTTCGATAGTTACAGCACAGCCAACATCTGGCTGGTGGTGGCCACCCGTGC
AGGCATGAATGCTTTCAATGACAGCGCACAGCCGGGGATGCCCGAGTGGAGCTTCCAGCGCTTCCCTACCA
ACACGTCAGCCTACCTGACGCTGGGTGCCATGCAGTACCACTACGGCTGCCCTAAACCCGATAGGGAGCTC
ACTGTGCTGCGCGTGGGCAGCGAGACCGGCTGTGCTGACAACATCTCTGTACCCAACTGCAATGGGCCCCT
GCCCGGCCCCGGGCCCTACTGGGTGAAGTTCCTCGCACTGAATGGCTCTGAGCCCACAGCCACCACAGAAT
GGTCGGGGCCCATCACACTGAAGACAGCCCGGGAGCCCCAGAGCATCCCCGGGATGGGCGGTGCCCGCAGC
GGTGCCATGATCGCCATCACTGCCATCCTGTCGGTGCTGTTGGCCATCCTGCTGGCCGCCCTCCTGGCCAC
GCTGTGCAG

>UPK3c.turtle profiling Ensemble scaffold:PelSin_1.0:JH207163.1:
TCTCTATCGACTATACACCTGTGATCACTGCCCGGGAGCTGGAGGGAAAGATCACCTCTTCCACCTTCGTC
CTGGAGCAGCCCCGCTGCGTCTTCAACGACTCGGTTAGCAACACCGACGAAATCTGGTTGGTGGTTGCTCT
TAGTAACGGTACGTCGATATCCACTTTCACCAACCCCACCTCCCTGCAGAGTCTGCCCGCTTTCCAGAAGT
TCCCAGGCAGCCCCCATTACATGACCATGGGCACCTCCTCCTTAAACTACCCCTGCGAAAAGAGCTCCGGC
CAGATCACTGTGCTGCGGGTTGGGAACGAAACCGGGTGCGTGTCGGACACGACGCGACCAGACTGCAATGG
CCCCCTGCCCGGCCTGGGGCCCTACAGGGTGAAGTTCCTTGCCATGAGTCCTGTCACGGGGCCCACGGCGG
AGACACGGTGGTCAGACCCGATTCTGCTGAAAGCAGGGAAGGACCCAGCTACCATTGACACCTGGCCCGGA
AAGCGCAGTGCCGGGATGATCGTCATCACCACCATCCTCTCCATCCTGCTGGCCATCCTGCTGGCCTGCTT
CATCGCCGCGCTGACCTACAGATG

>UPK2a.human. NM_006760.3
ACTTCAACATCTCAAGCCTCTCTGGTCTGCTGTCCCCGGCGCTAACGGAGAGCCTGCTGGTTGCCTTGC
CCCCCTGTCACCTCACAGGAGGCAATGCCACACTGATGGTCCGGAGAGCCAATGACAGCAAAGTGGTGACG
TCCAGCTTTGTGGTGCCTCCGTGCCGTGGGCGCAGGGAACTGGTGAGTGTGGTGGACAGTGGTGCTGGCTT
CACAGTCACTCGGCTCAGTGCATACCAGGTGACAAACCTCGTGCCAGGAACCAAATTCTACATTTCCTACC
TAGTGAAGAAGGGGACAGCCACTGAGTCCAGCAGAGAGATCCCAATGTCCACACTCCCTCGAAGGAACATG
GAATCCATTGGGCTGGGTATGGCCCGCACAGGGGGCATGGTGGTCATCACGGTGCTGCTCTCTGTCGCCAT
GTTCCTGCTGGTGCTGGGCTTCATCATTGCCCTGGCACTGGGCTCCCGCAAG

>UPK2a.mouse. NM_009476.2
ACTTCAACATCTCAAGCCTCTCTGGTCTGCTGTCTCCGGCGCTAACAGAAAGCCTGTTAATTGCCTTGCCC
CCATGTCACCTCACGGGAGGTAATGCCACATTGATGGTCCGGAGAGCCAACGACAGCAAAGTGGTTAAGTC
AGACTTTGTGGTGCCTCCATGTCGCGGGCGCAGGGAGCTTGTGAGCGTGGTGGACAGTGGGTCTGGCTACA
CCGTCACAAGGCTCAGCGCATATCAGGTGACAAACCTAACACCAGGAACCAAATACTACATATCCTACCGA
GTACAGAAGGGGACATCCACCGAGTCCAGTCCAGAGACTCCCATGTCCACGCTTCCTCGAAAAAACATGGA
GTCTATTGGGTTAGGAATGGCCCGGACAGGAGGGATGGTGGTCATCACAGTGCTGCTGTCTGTGGCCATGT
TCCTGTTGGTCGTGGGTCTTATTGTTGCCCTGGCACTGGGTGCCCGCAAA

>UPK2a.dog XM_848214.1
ACTTCAACATCTCAAGCGTCTCTGGTCTGCTGTCGCCGGCGCTAACGGAGAGCCTGCTAGTTGCCTTGCCC
CCCTGCCACCTCACAGGGGGCAACGCCACACTGATGGTCCGGAGAGCCAATGACAGCAAAGTGGTGAAATC
TAGCTTCGTAGTGCCTTCCTGCCGTGGGCGCAGGGAGCTGGTGAGTGTGGTGGACAGTGGGGCTGGCTTCA
CAGTCACCCGGCTCAGCGCATACCAGGTGACAAACCTCGTGCCAGGAACCAAATACTACATTTCCTACCTA

GTGAGGAAGGGGTCGTCCACCGAGTCCAGTAGAGAGATCCCAATGTCCACGCTGCCTCGAAGGCAGGCGGA
ATCCATTGGGCTGGGAATGGCCCGGACAGGGGGCATGGTGGTCATCACAGTGCTGCTCTCGGTCGCCATGT
TCCTGCTGGTTCTGGGCCTCATCATCGCCCTGGCCCTGGGCGCCCGAAAGTGA

>UPK2a.cow NM_174214.2
CTGACTTCAACATCTCAAGCCTCTCTGGTCTGCTGTCCCCAGTGATGACGGAAAGCCTGCTAGTTGC
CTTGCCCCCATGTCACCTCACAGGGGGCAACGCCACACTGACTGTCCGGAGAGCCAATGACAGCAAAGTGG
TGAGATCTAGCTTCGTGGTGCCTCCGTGCCGCGGACGCAGGGAGCTGGTGAGCGTGGTGGACAGCGGGTCT
GGCTTCACGGTCACCCGGCTCAGTGCATACCAGGTGACAAACCTGGCACCAGGAACCAAATACTACATTTC
CTACCTCGTGACAAAGGGGGCATCCACCGAGTCCAGCAGAGAAATCCCAATGTCCACATTTCCTCGAAGGA
AGGCAGAATCCATTGGGCTGGCAATGGCCCGGACAGGGGGCATGGTGGTCATCACGGTGCTGCTCTCGGTC
GCTATGTTCCTGCTGGTTCTGGGCTTGATCATTGCCCTAGCACTGGGCGCCCGAAAG

>UPK2a.elephant XM_003418177.1
ACTTCCACATCTCAAGCCTCTCTGGTGTGCTGTCCCCCGCGCTAACGGAGAGCCTGCTAGTTGCCTTGGCC
CCCTGTCGCCTCACTGGGGGCAATGCCACACTGATGGTCCGTAGAGCCAATGACAGCAAAGTGGTGAAATC
CAGCTTCATGGTGCCTCCATGCCGTGGGCACAGGGAGCTGGTGAGTGTGGTGGACAGTGGGGCTGGCTTCA
CAGTCACTAGGCTCAGTGCATACCAGGTGACAAACCTCGTGCCAGGAACCAAATACTACATTTCCTACCTA
GTGAAGAAGGGGACATCCACTGAATCCAGTAAAGAGAGCGCAATGTCCACGCTTCCTCGAAGGAAGATGGA
GTCCACTGGGTTGGGAATGGCCCGAACAGGGGGCATGGTGGTCATCACAGTGCTGCTCTCAGTCGCCATGT
TCCTGCTGGTTGTGGGCTTCATCACCGCCCTGGCACTGGGTGCCCAAAAG

>UPK2a.opossum XM_001380611.2
AATTCAACATCTCCAGTCTCTCTGGCCTGCTCTCCCCAGCCTTGGCAGAGAGCCTTCTAGTCGCCTTACCG
CCCTGCCATCTCACAGGGGGCAAAGCCAGTCTGACTGTCAGGAGAGTCAATGAAAGCGCAG
GAATGACGCACAACTTTACGGTCCCCCCCTGCCGAGCGCGAAGGGACCTGGTGAGTGTGGTGTACAACAGT
GGAAGCTTCTCCATCACCCGACTCAGTGCCTACCAAGTCACCAACCTGATACCCGGCACCAAATATTT
TGTCTACTATTCGGTGGAGAAAGGGACCGCTGTAGAGTCCAGTAACAAAGTCCAGATGGCCACACTGCCTC
GCCGAAAAGTGGAGACCCTGGGGCTGGGCATGGCCCGAACAGGGGGCATGATCGTCATCACGGTGCTGCTC
TCGGTCGCCATGTTCCTGCTGGTGGTGGGTTTGATCGTCGCTCTGGCCCTGGGTGTCCACAAA

>UPK2a.Platypus profiling AAPN01121652.1 exon2 AAPN01340066.1 exon3 |
AAPN01066803.1 exon4; AAPN01269979.1, exon5.
AATTTAACATCTCCAGTTTGTCCGGCCTGCTGACCCCGACCCTGGCGGAGAGCCTGCTGGTTGCCCTGCCC
CCTTGCCACCTCACGGGGGGAAATGCCACCCTGAAGGTCCAGGGAGTCAACGGCAGCTCAGTGCTTCAGCA
GAGGTTTGTGGTGCCACCGTGCCGTGGGCGCCGGGAGCTGGTCAGCGTGGTGGACAGCAGTGCCGGCTTCG
CCACCACCCGTCTGGACGCGTACCAGATCACCGGGCTGAGGCCGGCCACCACCTACGTNNNNNNNNNNNN
NNNNNNNNNNNNNNNNNNNNNNNNNNNNNNNNNNNNNNNNNNNNNNNNNNNNNNNNNNGGCAGAAGGCGG
AGCTGCTGGTGGTGGGCATGGCCCGCACGGGGGGCATGGTGGTCATCACGGTGCTGCTGTCTGTGGCCATG
TTCCTACTGCTCCTGGGTTTCATCGTTGCCCTCGTGCTGGGTTCCCACGACTGA

>UPK2.salamander C097794
AGACTGCACAGAATTTCAGCACCAGCCTAGCCGACCTCCCAATCAACCCACTGCAAACGCTCGCCATCGTT
GCCTTCCCACCATGCTGGGTCGGGGTCAGCCAGTCGAACGCCAACCTGATACTCGTTTCTAAAAACAGTTC
CGGGAGCAGCATTACAAATAACCAAACTGTCCCAGTCCCTCCGTGCCGCCTGAGAAGGGATGCAGTCTTTT
CGTCAGACAGCAGCAGTGGCGGGACCGTGATCACAAACATTGGTTTCAGAGTGACGAACTTGACAGCTAAT
ACAACCTACACGGCTTCGTACCAATCTAATGGCGTGACTATAGGATTGCCAACCAACTTCACGACTGTACA
ACCCACGAATTATACAGCCATGCCCGAGGTCTTTGCACGCAGCGGGGGGATGGTGGTCATCACAGTGCTGC
TGTCCATCGCCATGGCCATCCTGGTCATCGCCCTCATCCTCACCTTTGTGATGGGCCGCAAGAAG

>UPK2a.frogXl. BP683995
AGAATACTTCCCTGGCAGATGGGGTTCTTACTCCACTTAGTACATCTGTGATAATTGCATTTCCAGGATGC
AAAGACTCCGGAAAGACTGTTAACCTGATCGTAGCAAATGGCACAACTACTGTACAAAATATTTCCCTCCA
GGTACCACAGTGCCGCCTTAAACGAGATGTTGTTGTGATTAATAATTCACAGTCTGGTAATGTGCAGACTG
TGAATGTGGGCTATCAAATACAAAACCTACAACCAGGTGCCATCTACACGACGTATTATGCAGTTGACGGA
AGCAATATTCCATCCATTACATTTTCCACAAGATCAGTGTCTCAAACTGTCCCTGATATCATGGCTCGCAG
TGGAGGCATGGTGGTGATCACCGTCTTGTTAAGCATTTCAATGTTTGTGCTCTTGGTTGGATTAATCGCTG
TTCTTGTTATTGGAAGGAAA

>UPK2a.frogXt. genomic profiling;gb|AAMC01017077.1
scaffold_39_Cont17077,

AGAATGTTTCCCAGGCGACAGGGGTTCTTACTCCACTTGCTACATCTGCTATATTTGCTTTTCCAGACTGC
ACTTACTCAGGACAGACTGTTAGCTTGGTCATAACGAACAGCACATCTACTGCTACTATTATACAAAATGC
TACCTTCCAGGTACCACAGTGCCGCCTTAAACGTGATATTGTTGTGATTAATAATGGACAGTCCGGTAATG
TGCAGACTGTCAATGTGGGCTACCAGATCCAAAACCTACAGCCAGGTACCAACTACATGGCGACATATTCA
AGTGGTGGAATCTCTGGACCATCCTTCCAATTTAGTACAAGAACAGTGTATCCAGCTGTCGCAAATATAAT
GGCTCGCAGTGGAGGCATGGTGGTGATCACTGTCTTGTTAAGCATTGCGATGTTCGTGCTCTTGGCTGGGC
TAATCGCTGTTCTTATTCTTGGAAGGAAA

>UPK2a.coelacanth profiling gi|346202989|gb|AFYH01011847.1|
contig011847
ATTTCAACACCTCACTTTCTGACAATGCAGCGCTGATCTCTAACGTTTACAGCTCTTTTGTGATCCTGTCT
CTACCGCCCTGCACATACGCTGGAAAAAATGCAAGTGTTACCTACTCAAAGAACAGCACAAGTGG
ATAGCAAGACAGAAAGTTTTGTTGTTCCCCCCTGCCGGTTCAGACGCGAAGTTGTTGAAGTAGCCAGGCAA
ATGGAAGGTTTCACGGTTACTGACCTGCTGGGTTTCAGAGTGGGAAATCTCAAAGCTGGAACACAATATGA
TTTTCGTTATACAATTGACAACACAACCAACGTACTCAAAAGCAATATAATTCAAATTACAACCTCACTTG
TGACGAGTAATGTGCTCATCGACGAGGGGTTTAAATTGCACAGCGGTGGAATGATCGTCATAACTATCCTG
CTCTCCTTCGCCATGCTCTTCCTCATCATTGGTGTGATTGTCGTTCTAGTGTTGGGCAACAAAAGT

>UPK2a.Skate CV067582; AESE010437608.1
WGS_1_CONTIG_439473;AESE012541543.1| WGS_1_CONTIG_2704617;
AESE011527864.1|WGS_1_CONTIG_1648366,
AATTTACTATCTCGCTGGCGAATGACAATATGGGTGAGGTTGTGGCAAGTCGCCGCTCCATGTCAGCGATC
ATAACTATGGATCCTAACTCTTGCAATTTGGCCGGAGAGACTGTCATCGTCACCGTCAACAATACCTCAAA
TGGACAAGTAATAGCGCAACCCAACTTTGTGAGGCCCGTCTGTCGCAACAGACGTGATTTAATCAGTCTGG
TGTCTAACGCAGACGGAACCCCCCAAACATTGAACCTGGGCTACATGTTGGAGATGCTGCAACCAAGCACT
ACGTACAATGTGTATTTGAGAGCTGGCACCATAAGGAGCAACATGCTGGGAGTCACCACCATCTCACCGGT
GGACTACAGGACCATCGACCTTGGTTTTGGCCGCAGTGGGGCGATGGTGGTGATCACTGTGATTCTGTCTA
TCGCTATGTTGGCCCTCATCATCGCATTCATCGTTGTGCTTGTACTCAGCAAG

>UPK2.zebrafish EH444580, EH464141; gi|312125210:188150-200000
scaffold, Zv9_scaffold619
AAATACCAATCAGTTTGCTGAATCCTAACACTGATGGGGTGCTGGCGAGTACATTCCCCAACTCTTTTCTA
CTCCAAATGCCTGACTGCTCAATTTATGGAAACCAAAGTGTTTTACTGCTGTACACAGAAGCGCCCACCAA
TCTAAACAATACAGTGAATTTCACCGTTCAGCCTTGTCCTGTTAGTCAGTCGTGGTACCTTCTGGGTAACC
TCAAAAATGGCACAACATACAGCATGTCTTATAAAATTGGAAATGACACAAGCTCAGTTTTGACTAATACA
ACTACCAATGTCAATGATTACCAGCAGATTGACACTGGTCTTCGGGCACGCAGTGGAGCAATGGTGGTGAT
CACTGTCATCCTGTCTCTGGCCATGGTGTTCCTCCTGGTTGGCATTATTCTCGTCTTCTTCTTCTTCTCTG
GC

>UPK2.salmo EG847319 EST_ssal_eve_49206; AGKD01043111.1| Contig_043115
AATTCCAAGTGAGCCTTCTGAAAGAGTCAGATGGAGTTGTAACAGGCAGGTTTGCTGATTCTTTACTTTTG
AGCCTGCCTCCATGTGCTTTGGCAACACAGAGCGTGACTTTGGAGTACAACAACACTGACACCAACGAGAG
CAAAACTTTGGTCAATATATTCAAAGTGCTCCCCTGTCGGTTCAGAAGGGACATCATCTCAACCATTGAGA
ATAATGCCCAGTTCACTACCAGCAGAAACCTGGGTTATCAAGTGACAAATCTCACCACTGGCTCAACATAC
AGGTTTCAGTATGTGGTAGGGGCAGAGAAGAGCAACATTCTGGAGGTCTCTACAAGACAAGTCAAAGACCA
CAACCAAATAGACTCTGGCCTGCCAGCATGCAGTGGAGCCATGATGGTCATCACTGTCATACTGTCTGTTT
CCATGTTCATTTTGCTGGTAGCCCTCATTTTCACTGTTGCCCATTCCCTTGGTGGGGAT

>UPK2a.lamprey
ATTATCAGGTGAAACTGCTGAACACCAGCGTCGTTCGAGCGGCGCAGACACCTCAGTCCGTGGCCTTCGCA
GTGCCCTCGTGCGAGATCGCCATGATCGCGGGCACCATGCTCAACATCTTCGTGACGCCCAACACGACGGG
AGTCGAGGTGATCGTTGGGCAGGTGGCGGTGCCTGGGTGTCGCGTGGCGCGGGCCGTGTCGGGGCAGGTGG
TGTCTGGAAACGACGGGACGGGCCCGGGCATCCCCGTGCCCAGCGGAGTGGCCTATCGGGTCACGGGACTC
ACCCCCAGCACCAGTTACTCGGTGGTTCTCCGCCATTCAACGTTGGGATTGCAGTCTGTGCCCGGGATCCT
CAGCACGGCGAATGCGCGCACCCCCCGCAGCGCGGTGGACGCGGAGGCGTTCTCGCGCTCGGGAGGAATGG
TGGTGATCACGGTCATCCTCTCCGTCCTGATCTTCCTGCTCCTTGCCATCCTCGTCGCCGCCTTGCTGCTC
GGAAACAAAAAT

>UPK2b.snake profiling gb|AEQU010390653.1| contig26931230;
gb|AEQU010390653.1| Python molurus contig26931230; gb|AEQU010042415.1|
Python molurus

contig26234771CCATGGATTTCTTCCGTAACAACAGCATGCTTCTGACCACCTTCACGGGCACCACTT
TTATTGTTAATATTCCAAAATGTGTCTCTTCCTCCAAGTTTTCTCCAGCCACAGTCAGAATAGCAATCGCT
CAACTTCCGGATGCATCCTCTTTACCAGGTATAACAGATACTGACCAGATTGAAAACCTCCGCAGGACCCC
TCAGGCCCTGGTATATTTTGCAGATGAATTCAAGGACATCTCCTGCAGAGTGGCCAGAGACCTTCTGGTCC
TCGACCTGGATGATAGCCAATATGAGCTGATCACGGTGGTAGGCTACCAGGTGGGAGGAGAATTTTGCCGT
CAAACCAAAGGTCCCTTCTGCAACCAAGCTCTTAAGCCATCTACTTTTTATAGGGTGAACTTCTTTTTTTT
GGATGACAAGTCTGTGATAAGAGCCCATACGGATTGGTCAACAGCCATACAGACCAGGAATG
TGACCAACTACGAATCAGCTGATGTGATGTTCGAAGGGCGGGCAGGAGGAATGATAGTAATTACTATTCTG
GTCTCCGTTGGTGGGGCTGTCCTTCTCGTCGCATTAATCGTAGCAGTTGCTCTTTCCAGTAAGAAA

>UPK2b.turtle profiling |AGCU01117019.1| Pelodiscus sinensis
CACTAGATTTCTTTCCAAATGCAAGCCTCATTTTAGCAGCAAGAATGAGTACATCCTTCATTGTTAATATT
CCAAAGTGTATTTCTTCCTCTCAATTCACACCCACCACGATTAGACCTGCAGTAGCGATTGTTGGTGATAA
AGTTGCTATGCCAGCTGTGACAGACACCAATCAGATCCAATCCCTCCACTCTTCAAAATTCAAGAGTGTCT
CATGTAGGATAGCCAGGGATCTTGTGAGCATGGACATTGATGATGGGAACTACAAGCTGACCACAGTCGTA
GGCTACCAGGTGGGGGTAGAAGTCTGTGAAAAAACTAAAGGTCCCTTCTGCAATCAAGCCCTTCAGCCATC
GTCGGTTTACAGGGTAAATTTCTTTATTTTGGATGAAAAAGCGGTAATAAGAGCACACACAGGCTGGTCAG
ATGTCATACAAACGAACAATGTGACAACCTTCATGGCATATGATGGATCATTCATAGGGCGGGCTGGAGGA
ATGATAGTGATCACAGTACTGCTTTCTGTTGCTATGTTTGTGCTAGTCGTTGGCTTAATTGTAGCAGCAGC
TCTTGGAGGAAAAAAATCA

>UPK2b.lyzard XP_003221456.1; FG735771.1   gb|AAWZ02009508.1|
cont2.9507,
AACTGGACTTCTTTCCTAACAACAGCATGCTTTTGACAAGCTTCATGAGTACCTACTTCATTGTGAACGTT
CCAAAATGTGTTAGTCCCAAAGATTTTTCTCCTGTCAAAATCAGATTAGCAGTGGCTCAAGTTCCAGACAC
AAGCACTTTACCAGGTGTGATAGATACCGATGACATTGCAAATCTCCGCAGAACACCACAAGCGACTATAT
ATTTTGCTGGTGAATTTGATAGTGTCTCCTGTAGAGTAACCAGAGATCTTCTTGTAATGGACATGGATGAC
AGCCAATTTGAGCTGATCACAGTTTTAGGCTACCAGGTGGGAGCAGAATTTTGCCGTCAAACTAAAGGCCC
CTACTGCAATCAAGCCCTAAAGCCCTCCACATATTATAGGGTGAATTTCTTTTTTCTGGATAACAAATCTG
TAATAAGAGCTCATACGGATTGGTCAACAGCACTACAAACGAGAAACGTGTCAGATCATGAATCTGCTGAC
GTGATGTTCGGAGGACGAGCTGGAGGAATGATCGTAATCACCATTCTGGTTTCAGTTGGTGGGGCTGTCCT
AGCAATTGCCTTAATTGTAGCAGTAGCTCTTTCAAATAAAAAATAA

>UPK2b.aligator profiling gi|397270035|gb|AKHW01039964.1| scaffold-
4401_1,
CACTGAACTTCTTTCCTAACATGAGCAATATTTTAGCAGCAAGACTGAGTTCTTCCTTCATCGTTAATATC
CCTAAGTGTATCTCCTCCTCTCACTATACACCCACTACCATCAGACCAGCAATAGCTGTTCTTGGTGAGAC
AGTTGGCCTGCCAGCTGTGACAGACACCAACCAGATCAGATCCCTCCGCAACTTCACTGACGCCCCCATTT
ACTATGCGGGAGAATTCGCAAGTGTCTCATGCAGGATGGCCAGGGCTCTTGTGAAAATGGACATAGATGAT
GAGAACTATAAGCTGACCACAATTGTAGGCTACCAGGTGGGAGCAGAAGTCTGTGAAAACACTAAGGGGCC
CTTCTGCAATCGAGTCCTGAAGCCATCCTCATTCTACAGGGTGAATTTTTTTGTTTTGGATGAAAATGCTG
TAGTAAGAGCTCACACAGATTGGTCTGATCCCATCCAGACAAATAATGTGACAAGCTTCTCAGCCTATGAT
GGTTCATTTGAAGGGAGAGCAGGAGGAATGATAGTGATCACCGTGCTGCTTTCTGTTGGCATGTTCTTGGT
GGTGGTTGGTTTAATTGTGGCAGCAGCTCTTGGAGGTAGAAAATCC

>UPK2b.salamander >UPK2b.salamander JK979875, JK978999, JK977433,
CO783569
TCCCAGCATTCAGTTTCTTCGGGAACCAAGACAACATTATTGGCAATCGCATGGGCTATTCCTTCATCACC
AACATCCCCAGCTGCATTTCCGGGGCTGGATACACCCCAGCATCCATTCTGCTCGCAGTCTCGACCTCAGC
AGCGGTGCCAGGTGTATCAAACACAGATGCCATTAAATCTCTCCTCAACGACTCTAGAGCACAGGTGTACT
ATGCAGGTCAGTTCAACACCGTGCCCTGCAGAGTATCCCGCGATGTGGTGCAGGTGAGCAGCAAGGCAGCA
GACAACAGCTTCACCCTGACCACAGTCTTGGGGTACCAGGTGGGCTCAGAAGTCTGCACGAGCGTCAAGGG
CCTCTACTGCAACCAGGTTCTGGAGCCAGGAACCCCATACAGGGTCAATTTCTTTATTTTGGATGCCAGCA
ACGTTATTCGAGCCTACACCGATTGGTCTGACATTGTGACTACTTTAAATGTGACTAATAATGCACAACTA
GACTCTGGTCTTTCGAGGAGGTCTGGAGGAATGGTGGTCATCACAGTGCTTCTGTCGATTGCGCTCTTCAT
GCTGGTTCCAGCCTTGATTGCGACATTAGTTGTTGGGAGAGAAAAATCACCACTCCCC

>UPK2b.Xl DC123476, DY574479, BJ622989.
CTAATTTCCAGTTCTTCACAAACACGGACGATGTTCTGGGGGCAGTCCTGAGCCAATCGTTCATGGTGAAT
GTGCCCAGCTGTATCAACGCCGTGGGGTACGTGCCAAGCACCCTCAAAGTGGCTGTTGCAAACAGGAATCC

AACATGCATGGTAGACACAGACAGCATTAAATCTCTGAAAACGGACCCCACGGCCCCAGTCTACTACACAG
GGCAAATGAAGGTCCCACAGTGCCGACTCCGGAGAGATTTAGAGCCAGTGAAAATGAACAGCATGAGAGAT
CTGGGCTACCAGGTGGGAACGGAAAACTGCACGGAAGTCAGTGGACCTTTCTGCAACCAGTTTCTACAACC
AGGAACCTCTTATTGGGTGAACTTTATCATTTTGGATGAAACAGACACTCCAAGGGCATACACGGGCTGGT
CGGAACCCAGGACAACTAGACAAGTAAGACGTTTGGATGCCGTGGACCTTGGACTATCAGGTCACTCTGGT
GGAATGGTGATCATCACTGTACTGTTGTCAGTATCAGTCTTTCTGCTCCTCCTTGGATTTATGGCTGTTGT
AGTTGTCAGCAGA GCCCCAAACCTGTTCACTTCAGAGAGTAAGGATTGCTTGGACATGCAAACACAGCCT
