# Supplementary material for: Generation of divergent uroplakin tetraspanins and their partners during vertebrate evolution: identification of novel uroplakins
Source: BMC Evol Biol. 2014 Jan 23;14:13. doi: 10.1186/1471-2148-14-13 (PMC3922775; doi:10.1186/1471-2148-14-13)
Supplement: Additional file 3: Figure S3 — Phylogenetic trees of UPK2/3 and UPK1a/1b DNA and protein sequences generated using parsimony analysis. http://www.biomedcentral.com/imedia/1794860451103535/supp3.pdf. [file 1471-2148-14-13-S3.pdf]

Parsimony results

upk23_D bootstrap

```
/--------------------------------------------------------------- upk2 coela(1)
|
|                                                       /------- upk2 cow(2)
|                                                       |
|                                                       +------- upk2 human(3)
|                                                /--66--+
|                                                |      +------- upk2 dog(64)
|                                                |      |
|                                         /--99--+      \------- upk2 eleph(71)
|                                         |      |
|                                  /--84--+      \-------------- upk2 mouse(5)
|                                  |      |
+----------------96----------------+      \--------------------- upk2 oposs(6)
|                                  |
|                                  \---------------------------- upk2 Platy(75)
|
|                                                       /------- upk2 lampr(4)
+--------------------------78---------------------------+
|                                                       \------- upk3A plat(76)
|
+--------------------------------------------------------------- upK2 salam(7)
|
|                                                       /------- upk2 salmo(8)
+--------------------------51---------------------------+
|                                                       \------- upk2 zebra(12)
|
+--------------------------------------------------------------- upk2 skate(9)
|
|                                                       /------- upk2 Xlaev(10)
+--------------------------100--------------------------+
|                                                       \------- upk2 Xtrop(11)
|
|      /-------------------------------------------------------- upk23 lam1(13)
|      |
|      +-------------------------------------------------------- upk23 lam2(14)
|      |
|      +-------------------------------------------------------- upk23 lam3(15)
|      |
|      |                                                /------- upk3A alig(22)
|      |                                         /--80--+
|      |                                         |      \------- upk3A turt(32)
|      |                    /--------100---------+
|      |                    |                    \-------------- upk3A chik(23)
|      |                    |
|      |                    |                           /------- upk3A cow(25)
|      |                    |                           |
|      |                    |                    /--50--+------- upk3A dog(65)
|      |             /--92--+                    |      |
|      |             |      |             /--86--+      \------- upk3A elep(68)
|      |             |      |             |      |
|      |             |      |      /-100--+      \-------------- upk3A huma(26)
|      |             |      |      |      |
|      |             |      \-100--+      \--------------------- upk3A mous(27)
|      |             |             |
|      |             |             \---------------------------- upk3A opos(28)
|      |             |
|      |             +------------------------------------------ upk3A coel(24)
|      |             |
|      +-----51------+------------------------------------------ upk3A sala(29)
|      |             |
|      |             |                           /-------------- upk3A skat(30)
|      |             |                           |
|      |             +------------97-------------+      /------- upk3A Shar(62)
|      |             |                           \-100--+
|      |             |                                  \------- upk3A elpS(72)
|      |             |
|      |             +------------------------------------------ upk3A spot(31)
|      |             |
|      |             |                                  /------- upk3A Xlae(33)
```

```
|      |             \----------------100---------------+
|      |                                                \------- upk3A Xtro(34)
|      |
|      |             /------------------------------------------ upk3B alig(35)
+--61--+             |
|      |             +------------------------------------------ upk3B chik(36)
|      |             |
|      |             |                                  /------- upk3B cow(38)
|      |             |                           /--65--+
|      |             |                           |      \------- upk3B dog(66)
|      |             |                    /--56--+
|      |             |                    |      \-------------- upk3B elep(69)
|      |             |             /--86--+
|      |             |             |      \--------------------- upk3B huma(39)
|      |             |      /-100--+
|      |             |      |      \---------------------------- upk3B mous(40)
|      |             |      |
|      |             +--90--+----------------------------------- upk3B opos(41)
|      |             |      |
|      |             |      \----------------------------------- upk3B plat(77)
|      |             |
|      |      /--70--+------------------------------------------ upk3C chik(53)
|      |      |      |
|      |      |      |                                  /------- upk3C cow(54)
|      |      |      |                                  |
|      |      |      |                           /--69--+------- upk3C dog(67)
|      |      |      |                           |      |
|      |      |      |                    /--62--+      \------- upk3C elep(70)
|      |      |      |                    |      |
|      |      |      |             /-100--+      \-------------- upk3C huma(55)
|      |      |      |             |      |
|      |      |      |      /--76--+      \--------------------- upk3C mous(57)
|      |      |      |      |      |
|      |      |      +--83--+      \---------------------------- upk3C opos(58)
|      |      |      |      |
|      |      |      |      \----------------------------------- upk3C plat(78)
|      |      |      |
|      |      |      +------------------------------------------ upk3C Liza(56)
|      |      |      |
|      |      |      \------------------------------------------ upk3C turt(59)
|      |      |
|      |      +------------------------------------------------- upk3B coel(37)
|      |      |
|      |      |                                         /------- upk3B salm(42)
|      |      |                                  /-100--+
|      \--52--+                                  |      \------- upk3B trou(45)
|             |                           /--84--+
|             |                           |      \-------------- upk3B spot(44)
|             |                    /--82--+
|             |                    |      \--------------------- upk3B2 spo(52)
|             |                    |
|             |                    |             /-------------- upk3B zebr(48)
|             |                    |             |
|             +---------88---------+-----59------+      /------- upk3B1 sal(49)
|             |                    |             \-100--+
|             |                    |                    \------- upk3B1 tro(51)
|             |                    |
|             |                    \---------------------------- upk3B1 spo(50)
|             |
|             |                                  /-------------- upk3B skat(43)
|             |                                  |
|             +-----------------74---------------+      /------- upk3B Shar(63)
|             |                                  \-100--+
|             |                                         \------- upk3B elpS(73)
|             |
|             |                                         /------- upk3B Xlae(46)
|             |                                  /-100--+
|             |                                  |      \------- upk3B Xtro(47)
|             \-----------------96---------------+
|                                                |      /------- upk3B4 Xtr(60)
|                                                \--74--+
|                                                       \------- upk3B5 Xtr(61)
|
```

```
|                                                       /------- upk2L alig(16)
|                                                /--94--+
|                                                |      \------- upk2L turt(20)
|                                         /-100--+
|                                         |      |      /------- upk2L lyza(17)
|                                         |      \-100--+
|                                  /--80--+             \------- upk2L snak(19)
|                                  |      |
+----------------97----------------+      \--------------------- upk2L sala(18)
|                                  |
|                                  \---------------------------- upk2L Xlae(21)
|
\--------------------------------------------------------------- upk2 elepS(74)
```

upk23_P bootstrap

```
/---------------------------------------------------------- upk2 coela(1)
|
|                                                  /------- upk2 cow(2)
|                                          /--52---+
|                                          |       \------- upk2 dog(64)
|                                          |
|                                          +--------------- upk2 human(3)
|                                   /--98--+
|                                   |      +--------------- upk2 mouse(5)
|                                   |      |
|                            /--60--+      \--------------- upk2 elephant(71)
|                            |      |
+-------------87-------------+      \---------------------- upk2 oposs(6)
|                            |
|                            \----------------------------- upk2 Platypus(75)
|
|      /--------------------------------------------------- upk2 lampr(4)
|      |
|      +--------------------------------------------------- upk23 lam1(13)
|      |
|      +--------------------------------------------------- upk23 lam2(14)
|      |
|      +--------------------------------------------------- upk23 lam3(15)
|      |
|      |                                           /------- upk3A alig(22)
|      |                                   /--77---+
|      |                                   |       \------- upk3A turt(32)
|      |                     /-----97------+
|      |                     |             \--------------- upk3A chik(23)
|      |                     |
|      |                     |                     /------- upk3A cow(25)
|      |                     |                     |
|      |                     |                     +------- upk3A huma(26)
|      |                     |             /--67---+
|      |                     |             |       +------- upk3A dog(65)
|      |                     |             |       |
|      +---------74----------+      /-100--+       \------- upk3A elephant(68)
|      |                     |      |      |
|      |                     +--93--+      \--------------- upk3A mous(27)
|      |                     |      |
|      |                     |      \---------------------- upk3A opos(28)
|      |                     |
|      |                     +----------------------------- upk3A sala(29)
|      |                     |
|      |                     |                     /------- upk3A Xlae(33)
|      |                     \---------100---------+
|      |                                           \------- upk3A Xtro(34)
|      |
|      +--------------------------------------------------- upk3A coel(24)
|      |
|      |                                   /--------------- upk3A skat(30)
|      |                                   |
|      +----------------93-----------------+       /------- upk3A Shark(62)
|      |                                   \--100--+
|      |                                           \------- upk3A elepShark(72)
```

```
|      |
|      +--------------------------------------------------- upk3A spot(31)
|      |
|      +--------------------------------------------------- upk3B alig(35)
|      |
|      +--------------------------------------------------- upk3B chik(36)
|      |
|      +--------------------------------------------------- upk3B coel(37)
|      |
|      |                                           /------- upk3B cow(38)
|      |                                           |
|      |                                           +------- upk3B huma(39)
|      |                                   /--55---+
|      |                                   |       +------- upk3B dog(66)
|      |                                   |       |
|      |                            /--94--+       \------- upk3B elephant(69)
|      |                            |      |
+--54--+                            |      \--------------- upk3B mous(40)
|      +--------------63------------+
|      |                            +---------------------- upk3B opos(41)
|      |                            |
|      |                            \---------------------- upk3B platypus(77)
|      |
|      |                                           /------- upk3B salm(42)
|      |                                   /--100--+
|      |                                   |       \------- upk3B trou(45)
|      |                            /--80--+
|      |                            |      \--------------- upk3B spot(44)
|      |                     /--70--+
|      |                     |      \---------------------- upk3B2 spo(52)
|      |                     |
|      |                     +----------------------------- upk3B zebr(48)
|      |                     |
|      |                     |                     /------- upk3B1 sal(49)
|      +---------57----------+---------100---------+
|      |                     |                     \------- upk3B1 tro(51)
|      |                     |
|      |                     \----------------------------- upk3B1 spo(50)
|      |
|      +--------------------------------------------------- upk3B skat(43)
|      |
|      |                                           /------- upk3B Xlae(46)
|      |                                   /--100--+
|      |                                   |       \------- upk3B Xtro(47)
|      +----------------83-----------------+
|      |                                   |       /------- upk3B4 Xtrop(60)
|      |                                   \--96---+
|      |                                           \------- upk3B5 Xtrop(61)
|      |
|      +--------------------------------------------------- upk3C chik(53)
|      |
|      |                                   /--------------- upk3C cow(54)
|      |                                   |
|      |                            /--94--+       /------- upk3C dog(67)
|      |                            |      \--68---+
|      |                     /--55--+              \------- upk3C elephant(70)
|      |                     |      |
|      |              /-100--+      \---------------------- upk3C huma(55)
|      |              |      |
|      |      /--65---+      \----------------------------- upk3C mous(57)
|      |      |       |
|      +--76--+       \------------------------------------ upk3C opos(58)
|      |      |
|      |      \-------------------------------------------- upk3C platypus(78)
|      |
|      +--------------------------------------------------- upk3C Liza(56)
|      |
|      +--------------------------------------------------- upk3C turt(59)
|      |
|      |                                           /------- upk3B Shark(63)
|      +--------------------100--------------------+
|      |                                           \------- upk3B elepShark(73)
|      |
```

```
|      \--------------------------------------------------- upk3A platypus(76)
|
|                                          /--------------- upK2 salam(7)
|                                          |
+--------------------83--------------------+       /------- upk2 Xlaev(10)
|                                          \--100--+
|                                                  \------- upk2 Xtrop(11)
|
+---------------------------------------------------------- upk2 salmo(8)
|
+---------------------------------------------------------- upk2 skate(9)
|
+---------------------------------------------------------- upk2 zebra(12)
|
|                                                  /------- upk2L alig(16)
|                                          /--94---+
|                                          |       \------- upk2L turt(20)
|                                   /-100--+
|                                   |      |       /------- upk2L lyza(17)
|                                   |      \--100--+
|                            /--63--+              \------- upk2L snak(19)
|                            |      |
+-------------82-------------+      \---------------------- upk2L sala(18)
|                            |
|                            \----------------------------- upk2L Xlae(21)
|
\---------------------------------------------------------- upk2 elepShark(74)
```

UPK23_mixed

```
/---------------------------------------------------------- upk2 coela(1)
|
|                                                   /------ upk2 cow(2)
|                                             /-53--+
|                                             |     \------ upk2 dog(64)
|                                             |
|                                        /-70-+------------ upk2 human(3)
|                                        |    |
|                                  /-100-+    \------------ upk2 elephant(71)
|                                  |     |
|                            /-86--+     \----------------- upk2 mouse(5)
|                            |     |
+------------100-------------+     \----------------------- upk2 oposs(6)
|                            |
|                            \----------------------------- upk2 Platypus(75)
|
|                                                   /------ upk2 lampr(4)
|     /---------------------72----------------------+
|     |                                             \------ upk3A platypus(76)
|     |
|     |     /---------------------------------------------- upk23 lam1(13)
|     |     |
|     |     +---------------------------------------------- upk23 lam2(14)
|     |     |
|     |     +---------------------------------------------- upk23 lam3(15)
|     |     |
|     |     |                                       /------ upk3A alig(22)
|     |     |                                 /-92--+
|     |     |                                 |     \------ upk3A turt(32)
|     |     |                /------100-------+
|     |     |                |                \------------ upk3A chik(23)
|     |     |                |
|     |     |                |                      /------ upk3A cow(25)
|     |     |                |                      |
|     +-60--+                |                /-59--+------ upk3A dog(65)
|     |     |          /-97--+                |     |
|     |     |          |     |           /-97-+     \------ upk3A elephant(68)
|     |     |          |     |           |    |
|     |     |          |     |     /-100-+    \------------ upk3A huma(26)
|     |     |          |     |     |     |
|     |     |          |     \-100-+     \----------------- upk3A mous(27)
```

```
|     |     |    /-55--+           |
|     |     |    |     |           \----------------------- upk3A opos(28)
|     |     |    |     |
|     |     |    |     +----------------------------------- upk3A sala(29)
|     |     |    |     |
|     |     |    |     |                            /------ upk3A Xlae(33)
|     |     |    |     \------------100-------------+
|     |     \-71-+                                  \------ upk3A Xtro(34)
|     |          |
|     |          |                       /----------------- upk3A coel(24)
|     |          |                       |
|     |          |                       |    /------------ upk3A skat(30)
|     |          |                       |    |
|     |          \----------57-----------+100-+     /------ upk3A Shark(62)
|     |                                  |    \-100-+
|     |                                  |          \------ upk3A elepShark(72)
|     |                                  |
|     |                                  \----------------- upk3A spot(31)
|     |
|     |                /----------------------------------- upk3B alig(35)
|     |                |
|     |                +----------------------------------- upk3B chik(36)
|     |                |
|     |                |                            /------ upk3B cow(38)
|     |                |                      /-66--+
|     |                |                      |     \------ upk3B dog(66)
|     |                |                 /-69-+
|     |                |                 |    \------------ upk3B elephant(69)
|     |                |           /-86--+
|     |                |           |     \----------------- upk3B huma(39)
|     |                |     /-100-+
|     |                |     |     \----------------------- upk3B mous(40)
|     |                |     |
+-66--+                +-92--+----------------------------- upk3B opos(41)
|     |                |     |
|     |                |     \----------------------------- upk3B platypus(77)
|     |                |
|     +-------78-------+----------------------------------- upk3C chik(53)
|     |                |
|     |                |                            /------ upk3C cow(54)
|     |                |                            |
|     |                |                      /-95--+------ upk3C dog(67)
|     |                |                      |     |
|     |                |                 /-81-+     \------ upk3C elephant(70)
|     |                |                 |    |
|     |                |           /-100-+    \------------ upk3C huma(55)
|     |                |           |     |
|     |                |     /-87--+     \----------------- upk3C mous(57)
|     |                |     |     |
|     |                +-94--+     \----------------------- upk3C opos(58)
|     |                |     |
|     |                |     \----------------------------- upk3C platypus(78)
|     |                |
|     |                +----------------------------------- upk3C Liza(56)
|     |                |
|     |                \----------------------------------- upk3C turt(59)
|     |
|     +---------------------------------------------------- upk3B coel(37)
|     |
|     |                                             /------ upk3B salm(42)
|     |                                       /-100-+
|     |                                       |     \------ upk3B trou(45)
|     |                                  /-93-+
|     |                                  |    \------------ upk3B spot(44)
|     |                            /-82--+
|     |                            |     \----------------- upk3B2 spo(52)
|     |                      /-58--+
|     |                      |     \----------------------- upk3B1 spo(50)
|     |                      |
|     +----------99----------+                /------------ upk3B zebr(48)
|     |                      |                |
|     |                      \-------77-------+     /------ upk3B1 sal(49)
|     |                                       \-100-+
|     |
```

```
|     |                                              \------ upk3B1 tro(51)
|     |
|     +----------------------------------------------------- upk3B skat(43)
|     |
|     |                                              /------ upk3B Xlae(46)
|     |                                        /-100-+
|     |                                        |     \------ upk3B Xtro(47)
|     +------------------96--------------------+
|     |                                        |     /------ upk3B4 Xtrop(60)
|     |                                        \-92--+
|     |                                              \------ upk3B5 Xtrop(61)
|     |
|     |                                              /------ upk2L Shark(63)
|     +---------------------75-----------------------+
|     |                                              \------ upk3B elepShark(73)
|     |
|     \----------------------------------------------------- upk2 elepShark(74)
|
+----------------------------------------------------------- upK2 salam(7)
|
|                                                    /------ upk2 salmo(8)
+------------------------63--------------------------+
|                                                    \------ upk2 zebra(12)
|
+----------------------------------------------------------- upk2 skate(9)
|
|                                                    /------ upk2 Xlaev(10)
+------------------------100-------------------------+
|                                                    \------ upk2 Xtrop(11)
|
|                                                    /------ upk2L alig(16)
|                                              /-94--+
|                                              |     \------ upk2L turt(20)
|                                         /100-+
|                                         |    |     /------ upk2L lyza(17)
|                                         |    \-100-+
|                                   /-94--+          \------ upk2L snak(19)
|                                   |     |
\----------------97-----------------+     \----------------- upk2L sala(18)
                                    |
                                    \----------------------- upk2L Xlae(21)
```

UPK1_D bootstrap

```
/------------------------------------------------------------ upk1A human(1)
|
|   /-------------------------------------------------------- upk1A mouse(2)
|   |
|   |   /---------------------------------------------------- upk1A opossum(4)
|   |   |
|   |   |    /----------------------------------------------- upk1A platypus(7)
|   |   |    |
|   |   |    |                                          /---- upk1A aligator(8)
|   +95-+    |   /-----------------66-------------------+
|   |   |    |   |                                      \---- upk1A lizard(9)
|   |   |    |   |
|   |   |    |   |   /--------------------------------------- upk1A sala(10)
|   |   \-98-+   |   |
|   |        |   |   +--------------------------------------- upk1A frog(11)
|   |        |   |   |
|   |        |   |   +--------------------------------------- upk1A coela(12)
|   |        |   |   |
|   |        |   |   |                                  /---- upk1A zebraf(13)
|   |        |   |   |                              /100+
|   |        \97-+   |                              |   \---- UPK1A Carp(16)
|   |            |   |                         /100-+
|   |            |   |                         |    \-------- UPK1A CatFish(15)
|   |            |   |                     /100+
|   |            |   |                     |   \------------- upk1A salmo(14)
|   |            |   +--------100----------+
```

```
|   |            |   |                    \----------------- upk1A spotg(17)
|   |            |   |
|   |            |   +-------------------------------------- upk1A shark(18)
|   |            |   |
|   |            |   |   /---------------------------------- upk1A lamprey(19)
|   |            \70-+   |
|   |                |   +---------------------------------- upk1A2 lamprey(20)
|   |                |   |
|   |                |   |                             /---- upk1B human(21)
|   |                |   |                             |
|   |                |   |                             +---- upk1B mouse(22)
|   |                |   |                             |
+100+                |   |                         /91-+---- upk1B cow(23)
|   |                |   |                         |   |
|   |                |   |                         |   +---- upk1B dog(38)
|   |                |   |                /--100---+   |
|   |                |   |                |        |   \---- upk1B elephant(39)
|   |                |   |                |        |
|   |                |   |                |        \-------- upk1B opossum(24)
|   |                |   |                |
|   |                |   |                |        /-------- upk1B Gallus(25)
|   |                |   |                |        |
|   |                |   |                |   /-52-+   /---- upk1b turtle(27)
|   |                \94-+                |   |    \72-+
|   |                    |                +53-+        \---- upk1B aligator(28)
|   |                    |            /68-+   |
|   |                    |            |   |   \------------- upk1B liza(26)
|   |                    |            |   |
|   |                    |            |   +----------------- upk1b xlaev(29)
|   |                    |            |   |
|   |                    |            |   +----------------- upk1B sala(30)
|   |                    |            |   |
|   |                    |        /88-+   +----------------- upk1B coel(31)
|   |                    |        |   |   |
|   |                    |        |   |   \----------------- UPK1B skate(35)
|   |                    |        |   |
|   |                    |        |   |                /---- upk1B trout(32)
|   |                    |   /-97-+   |            /100+
|   |                    |   |    |   |            |   \---- upk1B salmo(33)
|   |                    |   |    |   \----100-----+
|   |                    |   |    |                \-------- upk1B spotg(34)
|   |                    \81-+    |
|   |                        |    \------------------------- upk1B platypus(37)
|   |                        |
|   |                        \------------------------------ upk1B lamprey(36)
|   |
|   +------------------------------------------------------- upk1A dog(5)
|   |
|   \------------------------------------------------------- upk1A elephant(6)
|
\----------------------------------------------------------- upk1A cow(3)
```

upk1_P bootstrap

```
/--------------------------------------------------------- upk1A human(1)
|
|   /----------------------------------------------------- upk1A mouse(2)
|   |
|   |    /------------------------------------------------ upk1A opossum(4)
|   |    |
|   |    |   /-------------------------------------------- upk1A platypus(7)
|   |    |   |
|   |    |   |                                       /---- upk1A aligator(8)
|   +-87-+   |    /----------------89----------------+
|   |    |   |    |                                  \---- upk1A lizard(9)
|   |    |   |    |
|   |    |   |    |                                  /---- upk1Asalamander(10)
|   |    \55-+    |                             /-93-+
|   |        |    |                             |    \---- upk1A frog(11)
|   |        |    |   /-----------56------------+
```

```
|   |        |    |   |                         \--------- upk1A shark(18)
|   |        |    |   |
|   |        |    |   +----------------------------------- upk1A coelacanth(12)
|   |        \-95-+   |
|   |             |   |                              /---- upk1A zebrafish(13)
|   |             |   |                         /-53-+
|   |             |   |                         |    \---- UPK1A Carp(16)
|   |             |   |                     /94-+
|   |             |   |                     |   \--------- UPK1A CatFish(15)
|   |             |   |                /-92-+
|   |             |   |                |    \------------- upk1A salmo(14)
|   |             |   |   /----100-----+
|   |             |   |   |            \------------------ upk1A spottedgar(17)
|   |             \97-+   |
|   |                 |   |        /---------------------- upk1A lamprey(19)
|   |                 |   |        |
|   |                 |   |        |                 /---- upk1B human(21)
|   |                 |   |        |                 |
|   |                 |   |        |                 +---- upk1B mouse(22)
|   |                 |   |        |                 |
|   |                 |   |        |            /-62-+---- upk1B cow(23)
|   |                 |   |        |            |    |
+100+                 |   |        |            |    +---- upk1BDog(38)
|   |                 |   |        |        /92-+    |
|   |                 |   |        |        |   |    \---- upk1B elephant(39)
|   |                 |   |        |        |   |
|   |                 |   |        |        |   \--------- upk1B opossum(24)
|   |                 \61-+        |        |
|   |                     |        |        |   /--------- upk1B Gallus(25)
|   |                     |        |        |   |
|   |                     |    /50-+        +54-+    /---- upk1b turtle(27)
|   |                     |    |   |        |   \-99-+
|   |                     |    |   |        |        \---- upk1B aligator(28)
|   |                     |    |   |        |
|   |                     |    |   |        +------------- upk1Blizard(26)
|   |                     |    |   |        |
|   |                     |    |   |        |        /---- upk1bXlaevis(29)
|   |                     |    |   |   /-94-+---62---+
|   |                     |    |   |   |    |        \---- upk1B salamander(30)
|   |                     |    |   |   |    |
|   |                     |    |   |   |    +------------- upk1B coelacanth(31)
|   |                     |    |   |   |    |
|   |                     \-90-+   |   |    |        /---- upk1B trout(32)
|   |                          |   |   |    |   /100-+
|   |                          |   |   |    |   |    \---- upk1B salmo(33)
|   |                          |   \100+    +100+
|   |                          |       |    |   \--------- upk1B spottedgar(34)
|   |                          |       |    |
|   |                          |       |    +------------- UPK1B skate(35)
|   |                          |       |    |
|   |                          |       |    \------------- upk1B platypus(37)
|   |                          |       |
|   |                          |       \------------------ upk1B Lamprey(36)
|   |                          |
|   |                          \-------------------------- upk1A2 lamprey(20)
|   |
|   +----------------------------------------------------- upk1A Dog(5)
|   |
|   \----------------------------------------------------- upk1A elephant(6)
|
\--------------------------------------------------------- upk1A cow(3)
```

UPK1_mixed bootstrap

```
/--------------------------------------------------------- upk1A human(1)
|
|   /----------------------------------------------------- upk1A mouse(2)
|   |
|   |   /------------------------------------------------- upk1A opossum(4)
|   |   |
|   |   |   /--------------------------------------------- upk1A platypus(7)
|   |   |   |
```

```
|   |   |   |                                       /---- upk1A aligator(8)
|   |   |   |   /----------------81-----------------+
|   +99-+   |   |                                   \---- upk1A lizard(9)
|   |   |   |   |
|   |   |   |   |                                   /---- upk1Asalamander(10)
|   |   |   |   |   /--------------72---------------+
|   |   |   |   |   |                               \---- upk1A frog(11)
|   |   |   |   |   |
|   |   \100+   |   +------------------------------------ upk1A coelacanth(12)
|   |       |   |   |
|   |       |   |   |                               /---- upk1A zebrafish(13)
|   |       |   |   |                           /100+
|   |       |   |   |                           |   \---- UPK1A Carp(16)
|   |       |   |   |                       /100+
|   |       |   |   |                       |   \-------- UPK1A CatFish(15)
|   |       |   |   |                   /100+
|   |       |   |   |                   |   \------------ upk1A salmo(14)
|   |       |   |   |   /------100------+
|   |       \100+   |   |               \---------------- upk1A spottedgar(17)
|   |           |   |   |
|   |           |   |   |   /---------------------------- upk1A lamprey(19)
|   |           |   |   |   |
|   |           |   |   |   +---------------------------- upk1A2 lamprey(20)
|   |           |   |   |   |
|   |           |   |   |   |                       /---- upk1B human(21)
|   |           |   |   |   |                       |
|   |           |   |   |   |                       +---- upk1B mouse(22)
|   |           |   |   |   |                       |
|   |           |   +56-+   |                   /94-+---- upk1B cow(23)
|   |           |   |   |   |                   |   |
+100+           |   |   |   |                   |   +---- upk1BDog(38)
|   |           |   |   |   |               /100+   |
|   |           |   |   |   |               |   |   \---- upk1B elephant(39)
|   |           |   |   |   |               |   |
|   |           \98-+   |   |               |   \-------- upk1B opossum(24)
|   |               |   |   |               |
|   |               |   |   |               |   /-------- upk1B Gallus(25)
|   |               |   |   |               |   |
|   |               |   |   |               +56-+   /---- upk1b turtle(27)
|   |               |   \99-+               |   \94-+
|   |               |       |           /71-+       \---- upk1B aligator(28)
|   |               |       |           |   |
|   |               |       |           |   +------------ upk1Blizard(26)
|   |               |       |           |   |
|   |               |       |           |   +------------ upk1bXlaevis(29)
|   |               |       |           |   |
|   |               |       |           |   +------------ upk1B salamander(30)
|   |               |       |           |   |
|   |               |       |       /59-+   |       /---- upk1B coelacanth(31)
|   |               |       |       |   |   \--52---+
|   |               |       |       |   |           \---- UPK1B skate(35)
|   |               |       |       |   |
|   |               |       |       |   |           /---- upk1B trout(32)
|   |               |       |   /100+   |       /100+
|   |               |       |   |   |   |       |   \---- upk1B salmo(33)
|   |               |       |   |   |   \--100--+
|   |               |       |   |   |           \-------- upk1B spottedgar(34)
|   |               |       \-98-+   |
|   |               |           |   \-------------------- upk1B platypus(37)
|   |               |           |
|   |               |           \------------------------ upk1B Lamprey(36)
|   |               |
|   |               \------------------------------------ upk1A shark(18)
|   |
|   +---------------------------------------------------- upk1A Dog(5)
|   |
|   \---------------------------------------------------- upk1A elephant(6)
|
\-------------------------------------------------------- upk1A cow(3)
```
