# Supplementary material for: Generation of divergent uroplakin tetraspanins and their partners during vertebrate evolution: identification of novel uroplakins
Source: BMC Evol Biol. 2014 Jan 23;14:13. doi: 10.1186/1471-2148-14-13 (PMC3922775; doi:10.1186/1471-2148-14-13)
Supplement: Additional file 4: Figure S4 — Phylogenetic trees of UPK2/3 and UPK1a/1b DNA and protein sequences generated using Bayesian analysis. http://www.biomedcentral.com/imedia/9084203271035356/supp4.pdf. [file 1471-2148-14-13-S4.pdf]

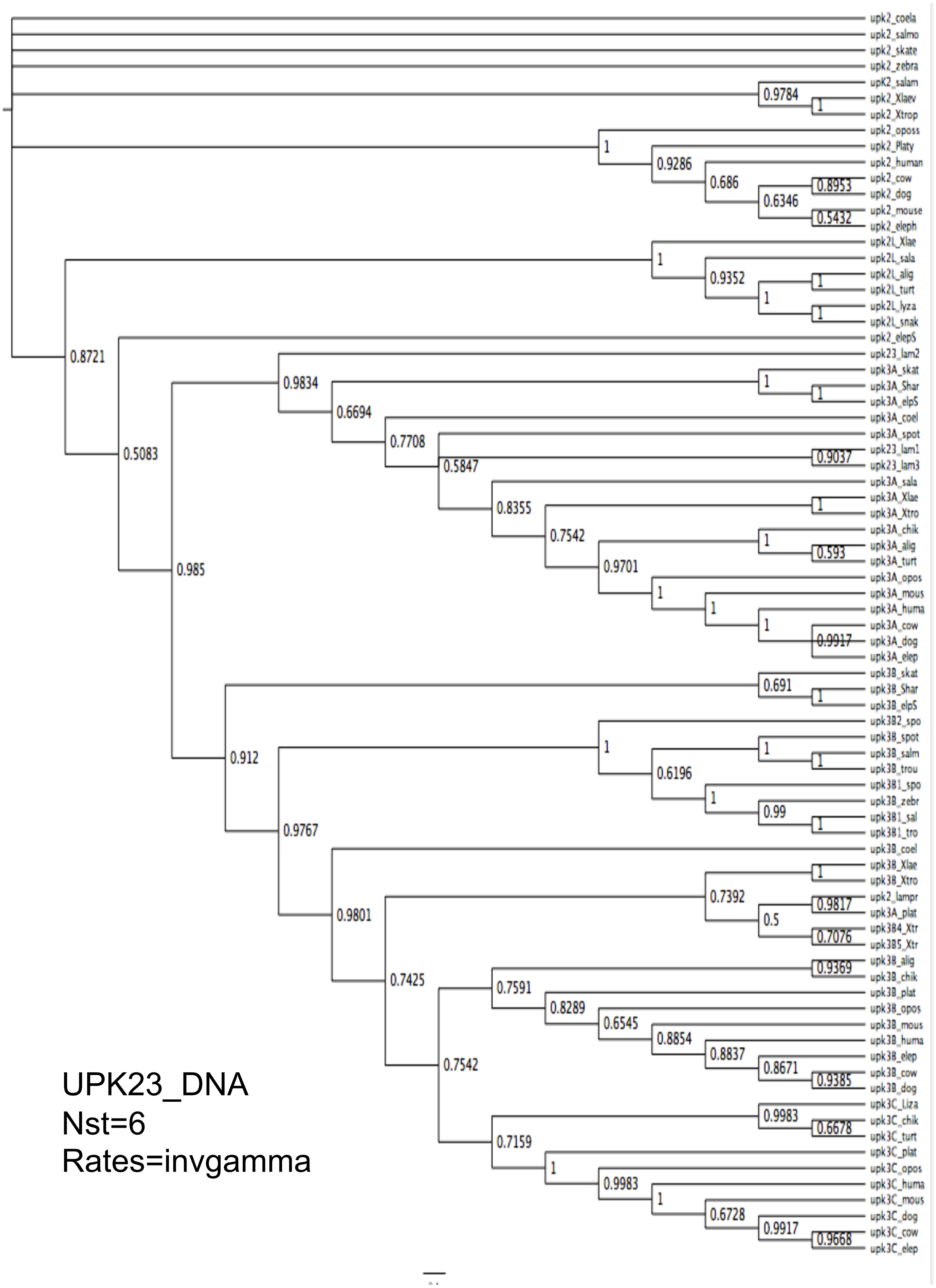

UPK23_DNA
Nst=6
Rates=invgamma

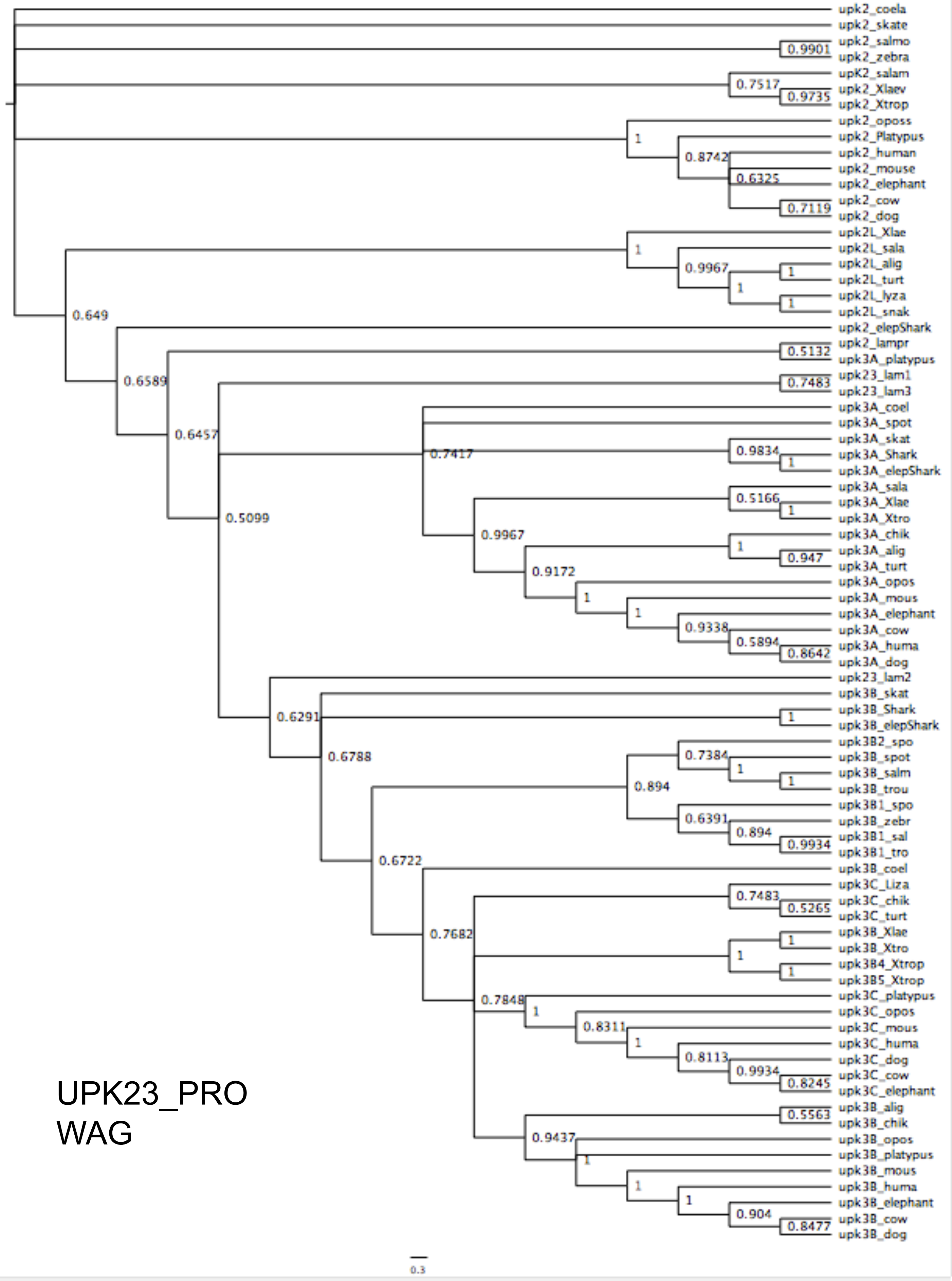

UPK23_PRO
WAG

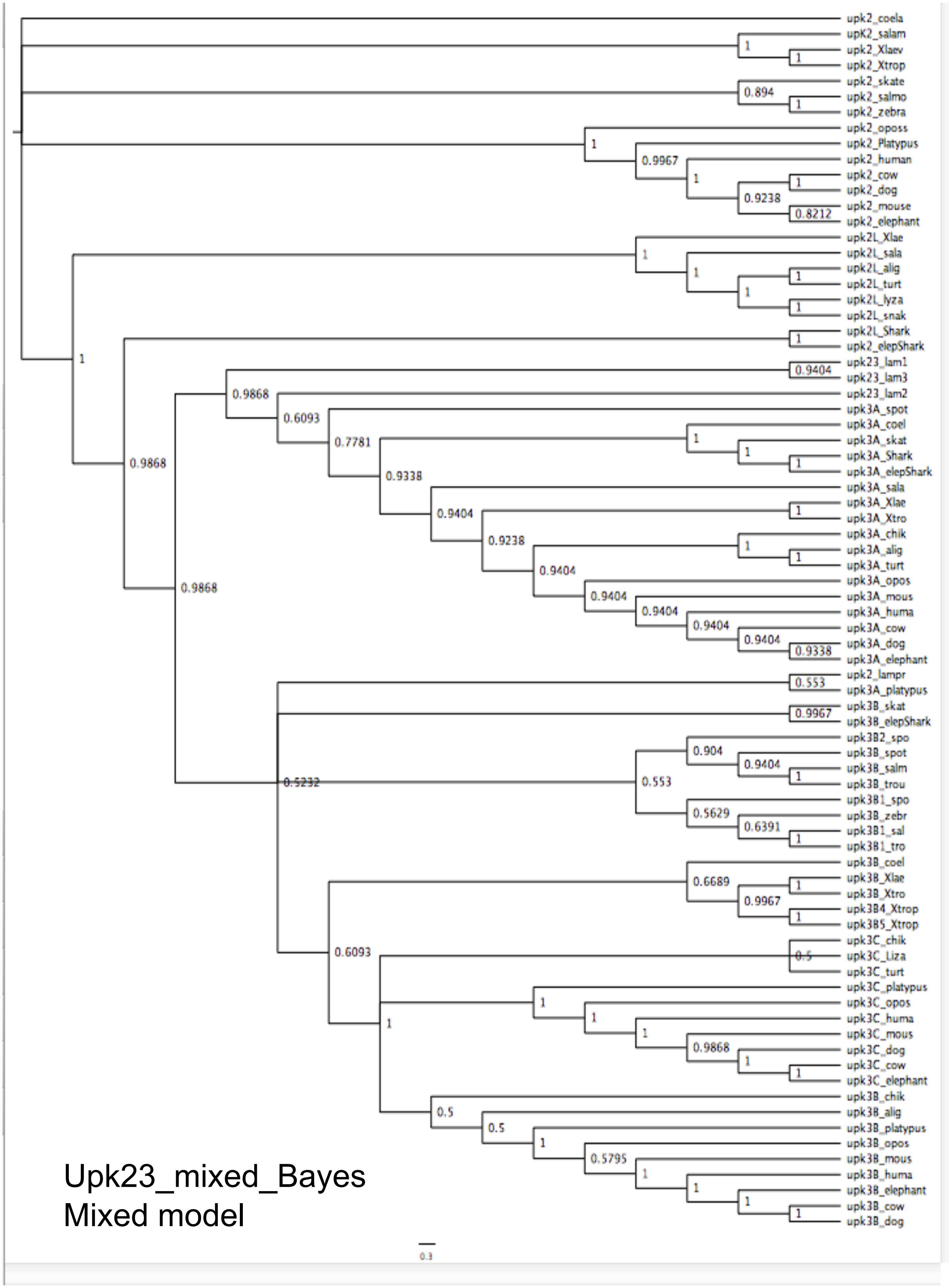

Upk23_mixed_Bayes
Mixed model

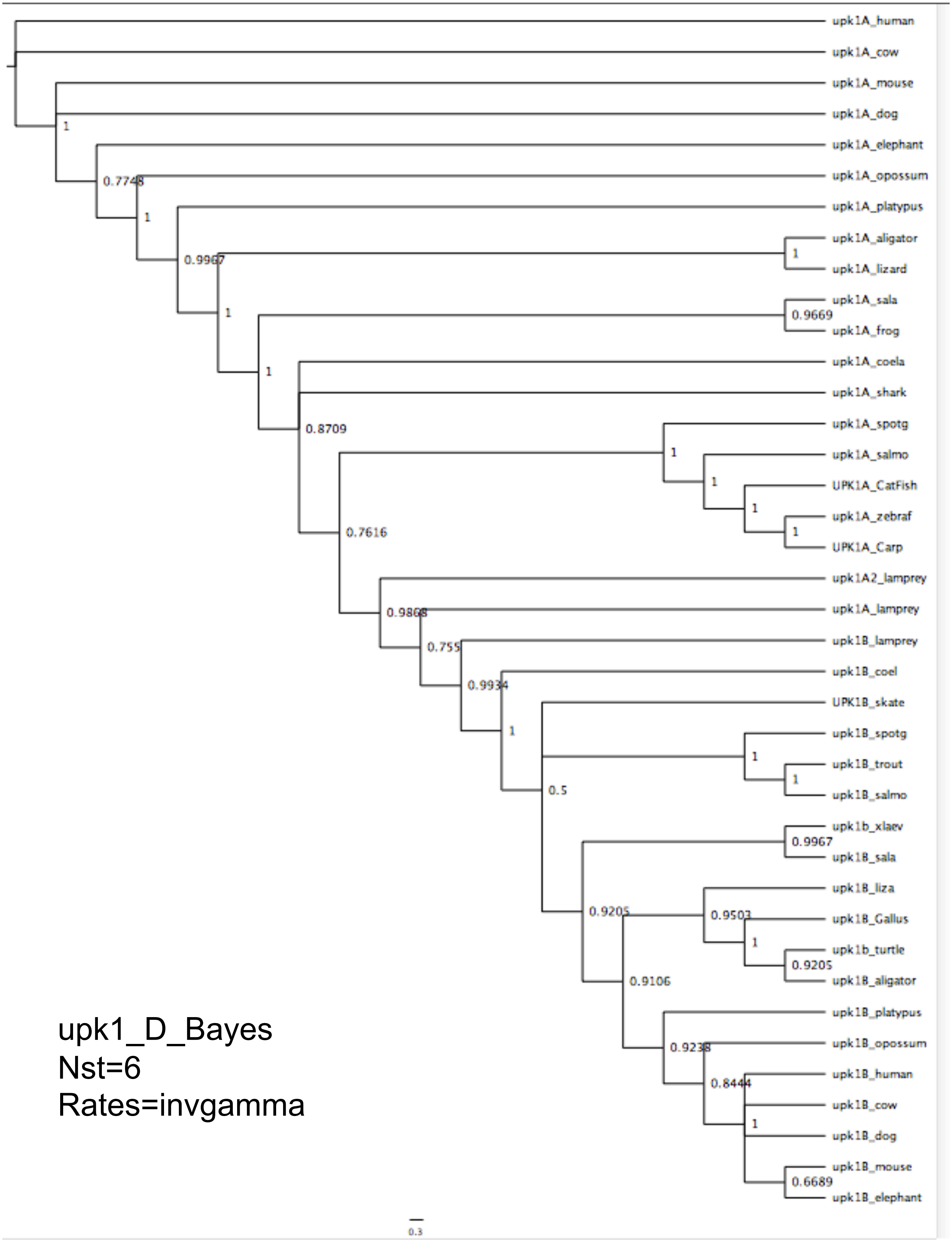

upk1_D_Bayes
Nst=6
Rates=invgamma

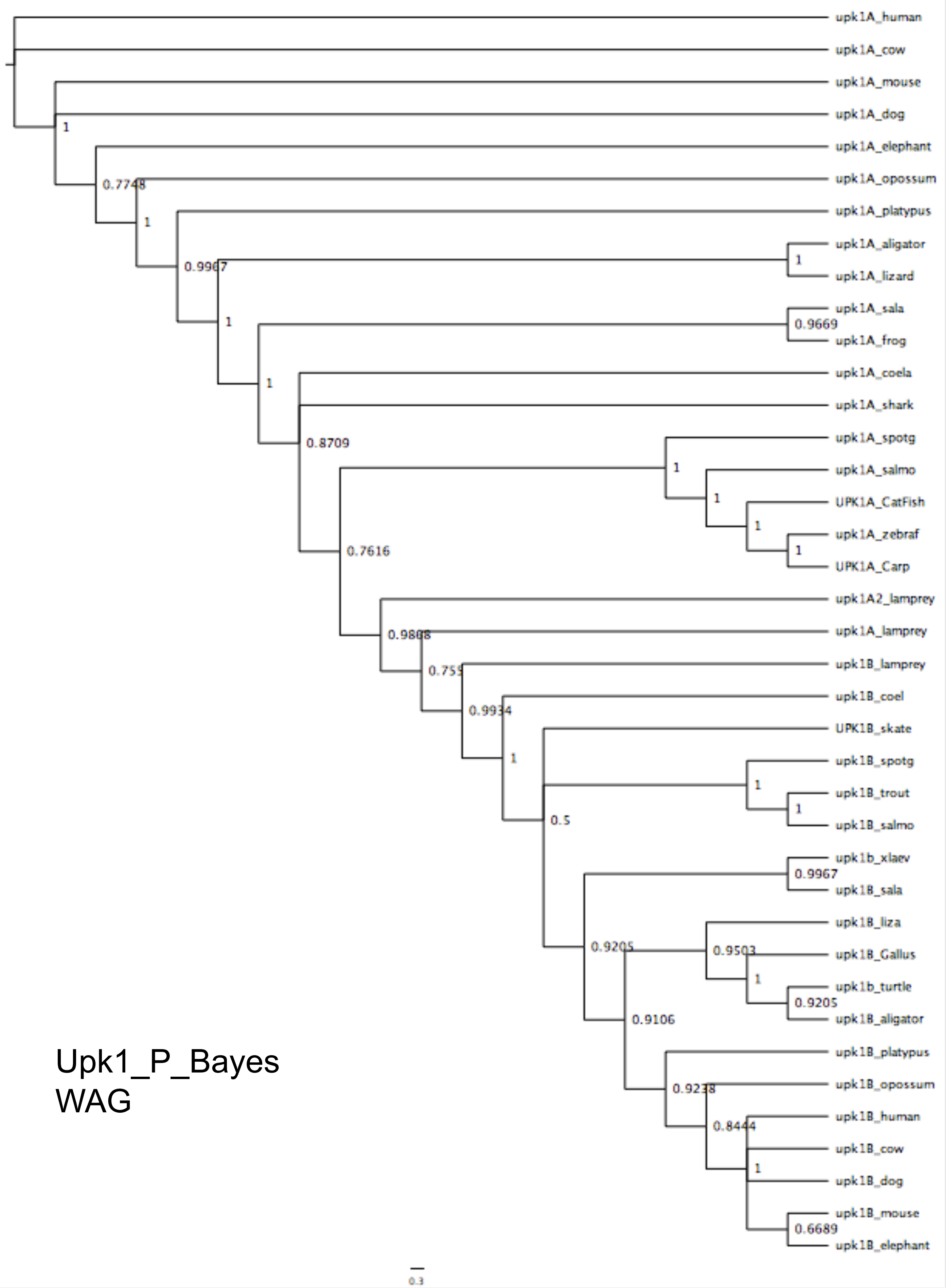

Upk1_P_Bayes
WAG

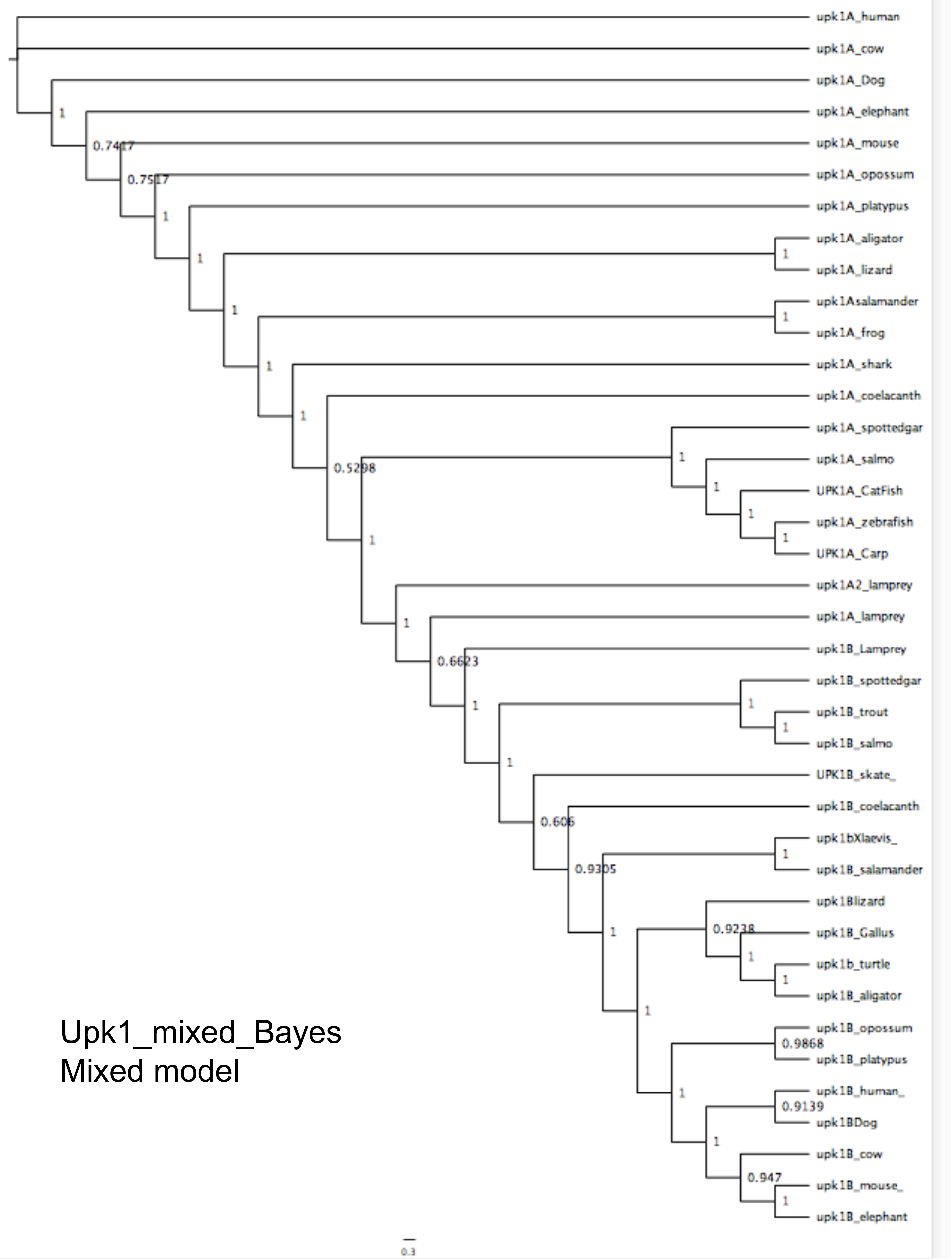

Upk1_mixed_Bayes
Mixed model
