# Supplementary material for: Generation of divergent uroplakin tetraspanins and their partners during vertebrate evolution: identification of novel uroplakins
Source: BMC Evol Biol. 2014 Jan 23;14:13. doi: 10.1186/1471-2148-14-13 (PMC3922775; doi:10.1186/1471-2148-14-13)
Supplement: Additional file 5: Figure S5. — Alignment of UPK3c and UPK3b full protein sequences. Exons are represented with alternate colors. Amino acids in red means they are split between two exons (intron phases 1 and 2). Intron phases are 1,1, 2, 1, 2. Asterisks indicate identical residues, dots indicate CLUSTALW conserved and semi-conserved substitutions. Highlighted in green transmembrane domains. Red box shared ~12 amino acid stretch between UPK3 uroplakins. Human UPK3c GenBank accession number KF150200. http://www.biomedcentral.com/imedia/1013243786103535/supp5.pdf. [file 1471-2148-14-13-S5.pdf]

```
UPK3c.Human        MDNSWRLGPAIGLSAGQSQLLVSLLLLLTRVQPGTDVAAPEHISYVPQLSNDTLAGRLTL 60
UPK3c.Cow          ----------MGLGRGQSPLLMALLLLLACLQMG---MSLERISYVPQLSSATLAGRLTQ 47
UPK3c.Opossum      ----------MGFPQGR--LLLLPLLLLTGIQTG---TSLEPINYTPAITREPLEGSITS 45
UPK3c.Gallus       -------------------MRPLLLLMLATAHG-----LDKLSYKPTLVGGNVEGRMTG 35

UPK3b.Opossum      ----------MGGAWGAP-FSLLLLLISYRTVTS--LGESDQIPYTPQISALALEGKVTA 47
UPK3b.Human.       ----------MGLPWGQPHLGLQMLLLALNCLRP--SLSLELVPYTPQITAWDLEGKVTA 48
                                           **:             : : * * :    : * :*

UPK3c.Human        STFTLEQPLGQFSSHNISDLDTIWLVVALSNATQSFTAPRT--NQDIPAPANFSQRGYYL 118
UPK3c.Cow          STFTLEQPRGQFSHPSISDSDAIWLVVAHSNATQKFTAPQK--VEDTPVPADFPQRGYYL 105
UPK3c.Opossum      STFTLDQPNDQFNGSGISDLDDIWLVVAFSNASQSFEPPQS--AQDIPYAATFLDKKYYL 103
UPK3c.Gallus       STFVLEQPRCVFDSYSTAN---IWLVVATRAGMNAFNDSAQPGMPEWSFQRFPTNTSAYL 92

UPK3b.Opossum      ATFSLEQPRCIFSELAAPA-DAVWLVVAFSNATEDFQNPKT--AAEIPSYTELSSSFYYM 104
UPK3b.Human.       TTFSLEQPRCVFDGLASAS-DTVWLVVAFSNASRGFQNPET--LADIPASPQLLTDGHYM 105
                   :** *:**   *.    .    :*****   . . *  .      : .          *:

UPK3c.Human        TLRANRVLY---QT---RGQLHVLRVGNDTHCQP--TKIGCNHPLPGPGPYRVKFLVMND 170
UPK3c.Cow          TLRASRALYPGGPP---SNQLRVLRVGNDTRCSP--RTRGCNRPLPGPGPYRVKFLVMSD 160
UPK3c.Opossum      TIRASRDLYSSKRG---SQGISVLRVGNETNCT----RSDCNKPLPGPGPYRVKFLVMNT 156
UPK3c.Gallus       TLGAMQYHYGCPKP---DRELTVLRVGSETGCADNISVPNCNGPLPGPGPYWVKFLALNG 149

UPK3b.Opossum      TLKLSPDLYPCEE-----EDIAVLRVGSDTNCLRNLSQEYCNAPLLAPGPYRVKFLVMDN 159
UPK3b.Human.       TLPLSPDQLPCGDPMAGSGGAPVLRVGHDHGCHQ---QPFCNAPLPGPGPYRVKFLLMDT 162
                   *:                    ***** :  *        ** ** .**** **** :.

UPK3c.Human        EG-PVAETKWSSDTRLQQ-AQALRAVP---GPQSPGTVVIIAILSILLAVLLTVLLAVLI 225
UPK3c.Cow          RG-PMAETEWSSETRLQQ-AEVLQAAP---GPQTAGTVVIIAILSVLLAVLLAALLALLI 215
UPK3c.Opossum      NG-PVAGTNWSEDITLRKPVEFSESRP---PSKSAGTIVIIAILSILLSLLFLALVALLV 212
UPK3c.Gallus       SE-PTATTEWSGPITLKT-AREPQSIPGMGGARSGAMIAITAILSVLLAILLAALLATLC 207

UPK3b.Opossum      NGQPKAETWWSDPITLNQ-GKDPRSIDTWPGRRSGCMIVITSILSTFAGLLVIAFLIAST 218
UPK3b.Human.       RGSPRAETKWSDPITLHQ-GKTPGSIDTWPGRRSGSMIVITSILSSLAGLLLLAFLAAST 221
                      * * * **    *.   .   :       ..   :.* :*** : .:*. .::

UPK3c.Human        YTCFNSCRSTSLSGPEEAGSV-----RRYTTHLAFSTPAEGAS----------------- 263
UPK3c.Cow          FTWYDTCGSTPISGPGELVFV-----RKYDTHHMSRPSTVGGS----------------- 253
UPK3c.Opossum      YTC---CGTEEISRPEESLRV-----RRYNTHHTYNSPDAGRN----------------- 247
UPK3c.Gallus       SSE--ACGVGSFR--PDAASI-----RRYNTHHVYDQPAARL------------------ 240

UPK3b.Opossum      VOFSSLWWPEDPS-PPEQLHIGSFIGKRYTTHHIPPSEADTLPVGRGAHLPPGPCPAPIV 277
UPK3b.Human.       MRFSSLWWPEEA---PEQLRIGSFMGKRYMTHHIPPSEAATLPVGCKPGLDPLPSLSP-- 276
                                  :   :     ::* **
```
